# CD4+ T cells from children with active juvenile idiopathic arthritis show altered chromatin features associated with transcriptional abnormalities

**DOI:** 10.1038/s41598-021-82989-5

**Published:** 2021-02-17

**Authors:** Evan Tarbell, Kaiyu Jiang, Teresa R. Hennon, Lucy Holmes, Sonja Williams, Yao Fu, Patrick M. Gaffney, Tao Liu, James N. Jarvis

**Affiliations:** 1 Department of Biochemistry, University at Buffalo Jacobs School of Medicine and Biomedical Sciences, Buffalo, NY USA; 2 Department of Pediatrics, University at Buffalo Jacobs School of Medicine and Biomedical Sciences, Buffalo, NY USA; 3 Genetics, Genomics, and Bioinformatics Program, University at Buffalo Jacobs School of Medicine and Biomedical Sciences, Buffalo, NY USA; 4 Genes and Human Disease Research Program, Oklahoma Medical Research Foundation, Oklahoma City, OK USA; 5 Enhanced Pharmacodynamics LLC, Buffalo, NY USA; 6 Department of Biostatistics and Bioinformatics, Roswell Park Comprehensive Cancer Center, Buffalo, NY USA

**Keywords:** Epigenetics, Epigenomics, Gene expression, Gene regulation, Genomics

## Abstract

Juvenile idiopathic arthritis (JIA) is one of the most common chronic diseases in children. While clinical outcomes for patients with juvenile JIA have improved, the underlying biology of the disease and mechanisms underlying therapeutic response/non-response are poorly understood. We have shown that active JIA is associated with distinct transcriptional abnormalities, and that the attainment of remission is associated with reorganization of transcriptional networks. In this study, we used a multi-omics approach to identify mechanisms driving the transcriptional abnormalities in peripheral blood CD4+ T cells of children with active JIA. We demonstrate that active JIA is associated with alterations in CD4+ T cell chromatin, as assessed by ATACseq studies. However, 3D chromatin architecture, assessed by HiChIP and simultaneous mapping of CTCF anchors of chromatin loops, reveals that normal 3D chromatin architecture is largely preserved. Overlapping CTCF binding, ATACseq, and RNAseq data with known JIA genetic risk loci demonstrated the presence of genetic influences on the observed transcriptional abnormalities and identified candidate target genes. These studies demonstrate the utility of multi-omics approaches for unraveling important questions regarding the pathobiology of autoimmune diseases.

## Introduction

Juvenile idiopathic arthritis (JIA) is a broad term that describes a clinically heterogeneous group of diseases characterized by chronic synovial hypertrophy and inflammation, with onset before 16 years of age^1^. JIA therefore represents an exclusion diagnosis that encompasses all forms of chronic childhood arthritis of an unknown cause. The reported prevalence of the disease is between 16 and 150 per 100,000 in the developed world, making it the most common rheumatic disease of childhood and one of the most common chronic illnesses in children^2^.

The causes of JIA are poorly understood, but both genetic and environmental factors are thought to play a role. A genetic component has been inferred from twin and affected sibling studies where concordance between monozygotic twins is between 25 and 40%^3,4^, and from numerous genome-wide association studies that demonstrate more than 40 regions associated with JIA ^5–9^. Like many complex diseases, many of the identified genetic variants that are associated with JIA are found outside of protein coding regions ^8,10^. It is possible that these variants alter the function of genomic regulatory elements, such as enhancers and promoters, which control gene expression and may contribute to disease initiation and progression. Our group has identified numerous transcriptional differences between JIA patients with active disease, those in clinical remission, and healthy controls in whole blood samples, peripheral blood mononuclear cells and neutrophils ^11–15^. In order to understand JIA pathogenesis, we believe that it is necessary to understand the events that lead to the transcriptomic alterations observed in children with JIA.

The control of transcription is a tightly choreographed process involving numerous cis- and trans- acting factors. Modifications to histone proteins may facilitate the recruitment of nucleosome remodeling complexes, which then evict nucleosomes and lead to remodeled chromatin^16^. Accessible chromatin can then be bound by sequence-specific transcription factors, which recruit co-factors, and mediate the three dimensional looping of DNA to bring distal regulatory elements into close physical contact with proximal promoters ^16–18^.

The CCCTC-binding factor (CTCF) is a sequence specific DNA binding factor, known for its role in establishing and maintaining long-range, three-dimensional chromatin interactions^19^. It was first categorized as an insulating factor, capable of preventing the interaction between enhancers and promoters in reporter constructs. More recently, CTCF has been shown to help establish topologically associated domains (TADs) and is involved in mediating promoter-enhancer interactions ^20^. Moreover, differential CTCF binding and the resulting differences in three-dimensional chromatin architecture have been implicated in disease pathogenesis for asthma and chemically induced cancers^21–23^.

Recent work has added an additional layer of complexity to transcriptional control. Transcriptional activation appears to occur within nuclear condensates, which contain high concentrations of transcription factors, co-factors and the general transcriptional machinery^24–28^. Moreover, single cell chromatin conformation capture studies have shown that one promoter or enhancer may interact simultaneously with multiple other promoters and enhancers^29,30^. These data suggest that multiple regulatory elements physically interact with each other in a hub-like complex, surrounded by high concentrations of transcription factors and transcriptional machinery ^31^.

## Results

### Widespread transcriptional changes in active disease and restoration with remission

**Figure 1:**
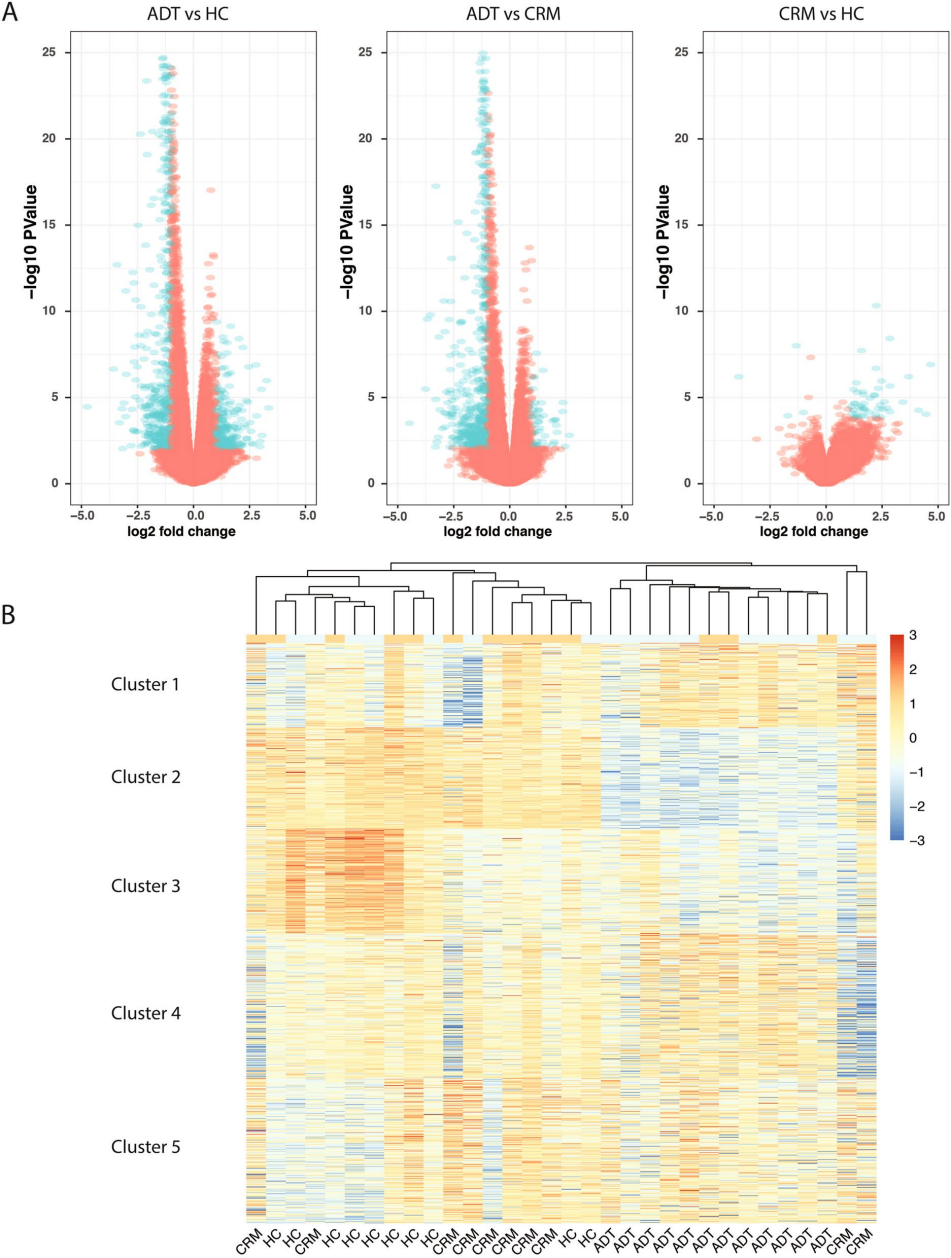
Attainment of clinical remission in JIA normalizes gene expression in CD4+ T-cells. (**A)** Volcano plots for each of the pairwise comparisons of gene expression between groups. (**B**) Heatmap showing the expression of all genes that were considered differentially expressed between any two groups. Kmeans clustering was applied to separate genes into one of 5 clusters and hierarchal clustering was applied to cluster the groups.

We performed RNA-seq on a cohort of 32 patients and healthy controls. This was a cross-sectional study that included 12 patients with active designated as having “active disease, treated” (ADT), 10 patients, who met the definition of clinical remission on medication (CRM) ^32–34^, and 10 healthy controls (HC) (See “Methods”). We identified 19,691 genes that were expressed in any group (See methods). We performed differential gene expression analysis on every pair of groups, identifying 693 genes that were differentially expressed in peripheral blood CD4+ T cells between ADT and HC (Supp. Table 1), 533 genes that demonstrated expression differences between ADT and CRM (Supp. Table 2), and 52 genes that were expressed differently between HC and CRM (Fig. 1A, Supp. Table 3). Of interest was the small number of genes that reached a threshold for differential expression between HC and CRM. We have previously noted that the attainment of clinical remission did not result in normalization of neutrophil transcriptomes ^11–15^. In the CD4+ T-cells examined here, there was considerable normalization between healthy controls and patients in remission. The pattern of differential expression between ADT and CRM or between ADT and HC was very similar, with most of the genes identified as up-regulated in the ADT group.

**Table 1:** Putative casual regulatory elements and target genes.

Feature chromosome	Feature start	Feature stop	Feature name	Target gene
chr1	154297035	154299035	ATP8B2	*TRIM46*
chr1	154322010	154323920	Peak_3422	LENEP
chr1	154322010	154323920	Peak_3422	PEAR1
chr1	154327990	154329950	Peak_3424	*CRTC2*
chr1	154352700	154353430	Peak_3426	*SLC50A1*
chr1	154355030	154363420	Peak_3427	EFNA1
chr1	154364920	154366710	Peak_3428	KIAA0907
chr3	46248832	46250832	CCR1	CCR2
chr3	46394234	46396234	CCR2	CCR1
chr3	46410632	46412632	CCR5	CXCR6
chr3	121813440	121820906	Peak_4095	*LRRC58*
chr3	46238451	46239841	Peak_4249	SMARCC1
chr3	46307161	46308651	Peak_4251	LARS2
chr3	46384411	46399021	Peak_4255	RTP3
chr3	46426891	46428451	Peak_4258	*PTPN23*
chr3	46442111	46444291	Peak_4261	NRADDP
chr3	46442111	46444291	Peak_4261	RTP3
chr3	46931478	46932061	Peak_4294	*PTPN23*
chr3	46931478	46932061	Peak_4294	RTP3
chr3	46967800	46974172	Peak_4299	*PTPN23*
chr3	119186784	119188784	POGLUT1	PARP9
chr3	119181529	119183529	TMEM39A	GOLGB1
chr3	119181529	119183529	TMEM39A	POLQ
chr5	131825465	131827465	IRF1	ACSL6
chr5	96220087	96239306	Peak_4706	CAST
chr5	96220087	96239306	Peak_4706	LNPEP
chr5	96244416	96249506	Peak_4707	ELL2
chr6	32406618	32408618	HLA-DRA	HIST1H4H
chr6	32406618	32408618	HLA-DRA	HLA-DMA
chr6	32406618	32408618	HLA-DRA	HLA-DMB
chr6	32406618	32408618	HLA-DRA	HLA-DOA
chr6	32406618	32408618	HLA-DRA	HLA-DPA1
chr6	32406618	32408618	HLA-DRA	HLA-DPB1
chr6	32406130	32408980	Peak_5195	PRRT1
chr6	32648860	32649480	Peak_5198	HLA-DMA
chr6	32648860	32649480	Peak_5198	HLA-DOA
chr7	28219075	28221075	JAZF1-AS1	CCDC126
chr11	36362500	36363860	Peak_298	ABTB2
chr11	36362500	36363860	Peak_298	*COMMD9*
chr11	36367840	36371740	Peak_299	CAPRIN1
chr11	36372980	36376021	Peak_300	*PRR5L*
chr11	36372980	36376021	Peak_300	*TRAF6*
chr12	6497390	6503150	Peak_808	POU5F1P3
chr14	69259631	69261472	ZFP36L1	KIAA0247
chr16	11376160	11381090	Peak_1171	RMI2
chr16	11400800	11410830	Peak_1173	RMI2
chr16	11424110	11425200	Peak_1175	RMI2
chr19	10449345	10451345	ICAM3	MIR199A1
chr19	10443314	10445314	RAVER1	ADAMTSL5
chr19	10443314	10445314	RAVER1	*ANO8*
chr19	10443314	10445314	RAVER1	*AP3D1*
chr19	10443314	10445314	RAVER1	*ATP13A1*
chr19	10443314	10445314	RAVER1	*C19orf53*
chr19	10443314	10445314	RAVER1	*CACTIN*
chr19	10443314	10445314	RAVER1	CARM1
chr19	10443314	10445314	RAVER1	*CHERP*
chr19	10443314	10445314	RAVER1	*COLGALT1*
chr19	10443314	10445314	RAVER1	*DNM2*
chr19	10443314	10445314	RAVER1	*DOT1L*
chr19	10443314	10445314	RAVER1	*FAM32A*
chr19	10443314	10445314	RAVER1	LPPR3
chr19	10443314	10445314	RAVER1	*MAP2K7*
chr19	10443314	10445314	RAVER1	MKNK2
chr19	10443314	10445314	RAVER1	*PIP5K1C*
chr19	10443314	10445314	RAVER1	*RASAL3*
chr19	10443314	10445314	RAVER1	RNF126
chr19	10443314	10445314	RAVER1	RPL36
chr19	10443314	10445314	RAVER1	RPS15
chr19	10443314	10445314	RAVER1	*STK11*
chr19	10443314	10445314	RAVER1	*SUGP2*
chr19	10443314	10445314	RAVER1	*TMEM259*
chr19	10443314	10445314	RAVER1	*TYK2*
chr19	10443314	10445314	RAVER1	*UBA52*
chr19	10443314	10445314	RAVER1	*UQCR11*
chr19	10443314	10445314	RAVER1	*ZBTB7A*
chr19	10490248	10492248	TYK2	*ABHD17A*
chr19	10490248	10492248	TYK2	ADAMTSL5
chr19	10490248	10492248	TYK2	*ANO8*
chr19	10490248	10492248	TYK2	*AP3D1*
chr19	10490248	10492248	TYK2	*C19orf53*
chr19	10490248	10492248	TYK2	*CACTIN*
chr19	10490248	10492248	TYK2	*CACTIN-AS1*
chr19	10490248	10492248	TYK2	*CC2D1A*
chr19	10490248	10492248	TYK2	*CCDC130*
chr19	10490248	10492248	TYK2	*DNM2*
chr19	10490248	10492248	TYK2	*FAM32A*
chr19	10490248	10492248	TYK2	*MAP2K7*
chr19	10490248	10492248	TYK2	*PIP5K1C*
chr19	10490248	10492248	TYK2	*RASAL3*
chr19	10490248	10492248	TYK2	*RAVER1*
chr19	10490248	10492248	TYK2	*REXO1*
chr19	10490248	10492248	TYK2	RFX1
chr19	10490248	10492248	TYK2	RNF126
chr19	10490248	10492248	TYK2	RPL36
chr19	10490248	10492248	TYK2	RPS15
chr19	10490248	10492248	TYK2	*SBNO2*
chr19	10490248	10492248	TYK2	*STK11*
chr19	10490248	10492248	TYK2	*SUGP2*
chr19	10490248	10492248	TYK2	*TMEM259*
chr19	10490248	10492248	TYK2	*UBA52*
chr19	10490248	10492248	TYK2	*UQCR11*
chr19	10490248	10492248	TYK2	*WIZ*
chr19	10490248	10492248	TYK2	ZNF414
chr22	30751626	30753626	CCDC157	*THOC5*
chr22	30751626	30753626	CCDC157	*UQCR10*
chr22	21914510	21916460	Peak_3818	*PPIL2*
chr22	21914510	21916460	Peak_3818	SDF2L1
chr22	30772430	30773260	Peak_3893	PISD
chr22	30782302	30784302	RNF215	EIF4ENIF1
chr22	30791929	30793929	SEC14L2	*DUSP18*
chr22	30791929	30793929	SEC14L2	FBXO7
chr22	30791929	30793929	SEC14L2	*SELM*
chr22	30791929	30793929	SEC14L2	*THOC5*
chr22	30791929	30793929	SEC14L2	*UQCR10*
chr22	30791929	30793929	SEC14L2	*ZMAT5*

The results of the integrative approach to identifying pathogenic targets. The chromosomal coordinates of the causal features are listed, along with their putative target genes. Targets shown in italic are considered differentially expressed between any of the conditions.

`We plotted all differentially expressed genes called between any pair of sample groups and used k-means clustering on the normalized expression values to group the genes into different gene clusters (Fig. 1B, Supp. Table 4). We also performed hierarchical clustering of the gene profiles of the individual samples. We find, as expected based on the differential analysis, that the CRM and HC groups clustered together and discretely from the ADT group. The gene clusters grouped into 5 distinct subgroups, representing different cellular functions (Supp. Table 5). Cluster 1, which was characterized by increased expression in ADT compared to HC, is involved in translation, indicating that in the active disease state, CD4+ T-cells are more active in their transcriptional and translational processes. Cluster 2, representing genes that were identified as showing lower expression in the ADT group compared the HC and CRM groups, were enriched for genes involved in signaling and response to stimuli. Cluster 3, which is characterized by higher expression in HC samples compared to either CRM or ADT samples, is enriched for genes involved in protein targeting and transport. Cluster 4, characterized by lower expression in HC compared to ADT and CRM, is enriched for genes involved in immune activation. Cluster 5 contained genes that showed a wide variety of expression patterns across the samples but show a general increase in expression in the ADT samples compared to the CRM and HC samples; this cluster is represented by genes involved in chromatin organization and silencing.

## Widespread differences in chromatin accessibility in active disease and remission

**Figure 2:**
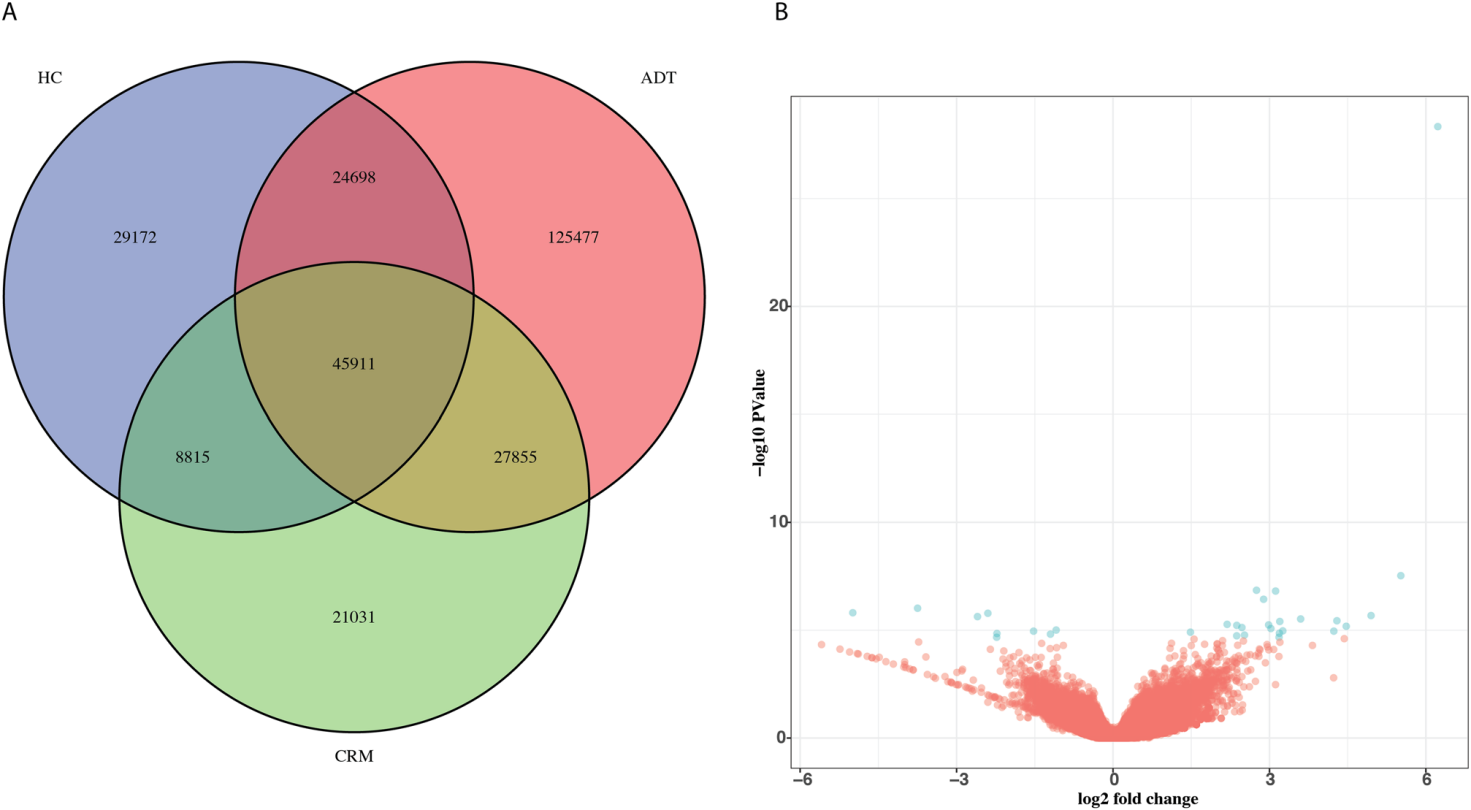
Chromatin re-arrangement occurs in the absence of three-dimensional changes. (**A**) Overlap between the group-consistent accessible peaks identified in HC, ADT and CRM samples. (**B**) Volcano plot showing the differences in CTCF binding affinity between 5 HC samples and 5 ADT samples. Blue dots represent sites whose log10(fold-change) was at least 1 and whose FDR was less than or equal to 0.05.

Having identified differentially expressed genes between the three conditions, we next sought to understand how the regulatory networks were perturbed and contributed to the differences in transcription. We performed the assay for transposase-accessible chromatin with sequencing ^35^ (ATAC-seq) on 6 HC, 5 ADT samples and 5 CRM samples, all of which were included in the RNAseq samples studied for gene expression. We identified 108,596 consistent peaks in our HC samples, 223941consistent peaks in our ADT samples and 103,612 consistent peaks in our CRM samples (Fig. 2A; see “Methods”). Of these, 45,911 peaks were shared by all three groups, indicating a significant level of chromatin reorganization associated with disease status. This data supports the transcriptomic data, in that there are significant differences in the expression and regulation of genes between JIA patients and healthy children. We also found considerable differences in the genomic accessibility of CRM patients versus healthy controls, indicating a high level of chromatin reorganization in remission despite modest changes in gene expression.

### Genetic variation in CTCF binding sites affects transcription of adjacent genes but does not seem crucial for the differences in expression data in active disease and healthy controls

CTCF is a critical genomic architectural protein that, with the aid of cofactors such as the cohesion complex, acts to mediate long-range chromatin interactions. These interactions are important for the establishment of regulatory domain boundaries, for mediating promoter-enhancer interactions, and the in the formation of insulator elements ^20^. There is also evidence that altered CTCF binding contributes to human disease ^21–23^. We hypothesized that differential CTCF binding, and the perturbed three-dimensional architecture that would result, may contribute to the transcriptional changes that we observed in the active disease. To test this hypothesis, we performed CTCF ChIP-seq on 10 patients not included in the first ATACseq and RNAseq studies. These included five ADT patients and 5 healthy controls. To directly investigate the three dimensional topology that is mediated by CTCF, we performed CTCF HiChIP on 6 participants, including 3 ADT patients and 3 healthy controls, of which one participant in each group was included in the CTCF ChIP-seq studies^36^. Using DiffBind^37^ to identify differential binding events, we identified 31 differentially bound CTCF sites using a threshold of FDR ≤ 0.05 and log10(fold-change) >  = 1 (Fig. 2B, Supp. Table 7). Moreover, none of these sites were located in the LD blocks around JIA associated variants ^9^.

We further characterized two of the differentially bound CTCF sites, whose log10(counts per million) > 5 and whose log10(fold-change) was > 4 (Supp. Table 7). Using HiChIP, we characterized a statistically significant gain of binding event, located in the intergenic region between the promoters of LCOR and PIK3AP1 on chromosome 10 (Supp. Figure 1A), where CTCF binding occurred in some of the ADT group and not in the HC group. The site showed binding of CTCF in three out of the five ADT samples and none of the healthy controls (Supp. Figure 1B). The loops that were anchored on this site in the ADT patients interacted with several differentially expressed genes (Supp. Figure 1C). To determine whether genetic variations may be driving the binding differences observed between the conditions, we performed Sanger sequencing^38,39^ on all samples across the identified locus. We found that all three ADT samples that had CTCF binding at the locus had a C allele at position chr10:98549577, whereas every sample that did not show CTCF binding, including all 5 HC samples and 2 of the 5 ADT samples, had the reference T allele at that location (Supp. Figure 1B). The gain of binding allele has been previously annotated as rs7477274. These data indicate that CTCF binding at this site is driven primarily by genetic variation.

We next identified a loss of binding event located in the promoter region of RNF135 on chromosome 17 (Supp. Figure 2A). The loops that are anchored on this site in healthy samples interact with the promoter regions of the differentially expressed genes (Supp. Figure 2C) and non-differentially expressed genes. The three HC samples that have CTCF binding at this site have a G allele at position chr17: 29297397, annotated as rs7221217 (Supp. Figure 2B), which was confirmed through Sanger sequencing. Of the three samples with CTCF binding, two were homozygous and one was heterozygous (Supp. Figure 2B). The two HC samples without binding at this site, as well as all five ADT samples that have no CTCF binding, were all homozygous with the reference A allele (Supp. Figure 2B). This data indicates this loss of binding is mediated by genetic variation.

Overall, this data shows that differential CTCF binding and altered three-dimensional interactions occur and are associated with genetic variants and with differentially expressed genes. However, the rarity of the events and the fact that none occur in known JIA risk loci indicate it is unlikely to be a driving mechanism of transcriptional aberrations in in CD4+ T-cells in JIA.

## Identifying interaction domains with CTCF ChIP-seq and HiChIP

Our profiling of the CD4+ T cell genome has yielded several features not previously recognized. We observed widespread transcriptional differences in CD4+ T cells in the comparison between active JIA and healthy controls, widespread changes in chromatin accessibility between all three conditions. We did not observe widespread changes in CTCF binding and, therefore, in the three dimensional structure of the genome. We thus hypothesize that due to the lack of three dimensional interaction differences, the variability in gene expression patterns must be a function of the variability in the activity of regulatory elements. However, due to the small expression changes observed between healthy controls and patients in remission and the widespread changes in accessibility between those groups, we further hypothesize that only a small fraction of the regulatory variability accounts for the differences in expression. We therefore focused next on previously identified JIA risk loci and employed a novel method based on emerging models of gene regulation to identify the regulatory elements and genes driving the observed genomic differences.

**Figure 3:**
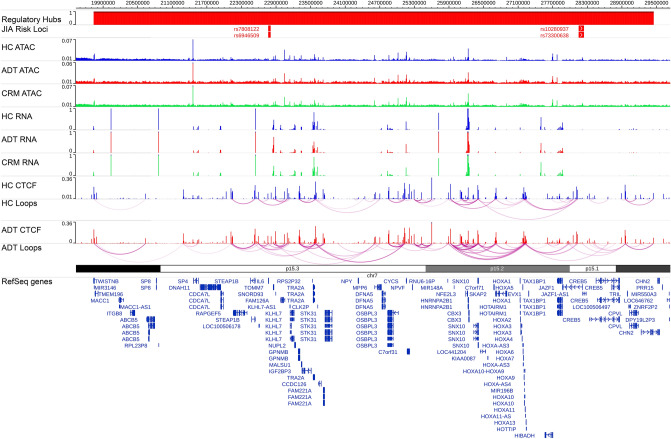
Identifying regulatory hubs. Snapshot showing the regulatory hub anchored on JIA risk variants rs6946509, rs7808122, rs10280937 and rs73300638. Top row shows LD blocks of the risk variants, followed by averaged ATAC-seq, RNA-seq, CTCF ChIP-seq signal across the three sample groups, as well as the HiChIP loops for the healthy control and active disease groups, followed by differentially expressed genes and all RefSeq genes.

Recent studies, as noted previously, have suggested that transcription occurs within “regulatory hubs”, containing high concentrations of transcriptional machinery, and multiple genomic regions that physically interact with each other^24–31^. Additional studies have shown that genomic regulatory regions could simultaneously interact with numerous other elements and gene regions^29–31^. . Our data identify multiple regions connected to one another through chains of CTCF-mediated loops (Fig. 3). These observations challenge the paradigm that transcriptomic regulation is mediated exclusively by differential three-dimensional contacts^31^. Instead, numerous regulatory elements and gene promoters interact together in regulatory hubs, with intra-hub regulation occurring through a mechanism other than simple physical proximity. With this concept in mind, we attempted to understand transcriptomic regulation by modeling the expression of a gene as a function of the accessibility of all regulatory elements that physically interacted with the gene promoter and used feature selection to identify elements contributing to variability in the gene’s expression (see methods).

Genome-wide association studies (GWAS) and genetic fine mapping studies have identified at least 46 genetic loci that are associated or suggestively associated with JIA^7,40^. Linkage disequilibrium blocks, genomic regions with highly correlated patterns of genetic heritability, are enriched for histone marks associated with regulatory activity ^9^. We found that 33 out of 46 of LD blocks around previously identified JIA genetic risk loci^7,9,40^ had regulatory elements and CTCF loop anchors located within them (Fig. 3). This indicates that some of the genetic risk for JIA may act by affecting CD4+ T-cell function.

Starting with the 33 JIA risk loci that contained CTCF loop anchors, we identified the regulatory hubs of which they were a part (Fig. 3) and predicted which regulatory elements controlled the gene’s expression (see “Methods”). We then considered any regulatory element that was within the JIA associated LD block as a putative casual element, and that any genes that were predicted to be controlled by that element as putative target genes for the JIA risk locus (Table 1). The total set of predicted target genes showed strong enrichment for gene ontology (GO) terms such as antigen presentation and T cell regulation and proliferation, while differentially expressed predicted target genes showed enrichment for cell signaling and gene regulation pathways (Supp.Tables 2–3). This enrichment for immune system pathways supports our predictions for JIA target genes. We believe that these putative targets and casual elements represent a starting point for understanding the genomic pathogenesis of JIA.

## Discussion

In this study, we observed widespread changes in gene expression profiles and chromatin accessibility in CD4+ T cells of patients with active JIA when we compared them to those of healthy controls. These differences reveal several important features of JIA. The first is that the transcriptional abnormalities that are observed in other cell types, such as neutrophils ^11,14^, PBMCs^41^ and whole blood samples ^15^ are present within the CD4+ T cell compartment. It is likely that the abnormalities reflect differences within similar cell populations between disease states, as opposed to differences in the proportions of naïve, memory or regulatory T cell subtypes, as evidenced by recent work showing little change in cell subtype ratios between patients with JIA and controls^42,43^. Second, the transcriptional differences reflect activation of peripheral blood immune components, which implicates a global immune dysfunction, as opposed to an isolated process involving the joints or resident lymphocytes within the synovium^44^.

We have shown that the CD4+ T cells of children with JIA display a three dimensional genomic architecture that is similar to that in healthy children. Alterations in CTCF binding, and the corresponding changes in loop structures, are observed and are associated with genetic variants, but are rare events that are not associated with any known JIA genetic risk locus. These findings, combined with the widespread changes in chromatin accessibility that we observed, point to a mechanism where disrupted regulatory elements may drive gene expression changes, rather than a rewiring of the three dimensional genome.

These features pose a critical question to our understanding of JIA: does this activation of peripheral immune cells simply reflect the brisk inflammatory milieu of the synovial tissues or is it an intrinsic immune dysfunction? Recently, researchers have observed an enhanced IFNγ signaling phenotype in the CD4+ T cells of active JIA patients ^43^, suggesting that an intrinsic dysfunction is occurring. We provide further evidence of an intrinsic dysfunction by showing that more than half of JIA associated genetic risk LD blocks contain T-cell specific regulatory elements that physically interact with other regulatory elements and gene promoters. Leveraging this feature allowed for the prediction of numerous putative target genes, which may have pathogenic roles in JIA.

One of the surprising findings from this work was the relative normalization in expression patterns that we observed in CD4+ T cells from patients who have achieved clinical remission while on MTX and etanercept. This stands in contrast to what we have observed in other cell populations, especially neutrophils ^41^. This leads us to an interesting hypothesis concerning CD4+ T-cells in JIA and their role in the disease. We hypothesize that CD4+ T-cells are “primed” for a hyper-inflammatory response, due to the effect of genetic variations on the expression of our predicted target genes. When CD4+ T-cells interact with another cell type or with molecules secreted by other cells, they initiate an inflammatory response, which progresses unchecked to an elevated level. We hypothesize that pharmacological intervention acts by disrupting the extracellular signaling, returning the CD4+ T-cells to a resting, but “primed” state, while other cell types remain altered.

We recognize that the present study has several limitations. The first is the fact that this is a cross-sectional study and therefore it is not known whether the differences in chromatin organization that we observed change through the course of disease progression or through treatment response. Another limitation of this study is the small number of patients and the fact that the same assays were not performed on every subject. These limitations largely reflect the constraints of undertaking translational studies in a pediatric population. Given the limited sample volumes that can be obtained from children, investigators are limited in the number of studies that can be performed on any individual patient sample. We are sanguine, however, that continued technologic advances will allow us to perform more complex studies on larger groups of patients in the near future. However, the validity of our approach is shown in the fact that that datasets from one group of participants corroborated the inferences made with an independent set of participants.

## Methods

### Patients and patient samples

Patients were recruited from the pediatric rheumatology clinic at the Women & Children’s Hospital of Buffalo. All children fit criteria for polyarticular-onset, rheumatoid factor-negative JIA as established by the International League Against Rheumatism (ILAR) ^45^. Patients included 18 girls and 4 boys who ranged in age from 4 to 16 years. Children with JIA were classified as having active disease on therapy (ADT) or clinical remission on medication (CRM) as determined by standard criteria ^32–34^. Children with JIA were all taking combinations of methotrexate and the TNF inhibitor, etanercept. Samples were obtained from children classified as ADT within 6 weeks after initiating therapy. All children classified as ADT had at least 4 warm, swollen joints. Samples obtained from children with CRM were obtained at the first clinic visit at which CRM status was confirmed, typically 12–15 months after the initial diagnosis.

Healthy control (HC) children (n = 10) were recruited from the Hodge General Pediatrics Clinic of the University at Buffalo Jacobs School of Medicine and Biomedical Sciences and ranged in age from 5 to 16 years and included two boys and eight girls. Children were excluded if they had fever ≥ 38 °C within the previous 48 h, had another autoimmune disease (e.g., type 1 diabetes), or were taking either systemic glucocorticoids or antibiotics for any reason.

University at Buffalo IRB approval was obtained for this study, and informed consent documents executed with the parents of all patients and controls. For children over the age of 7 years, age-appropriate assent documents were also executed. All research procedures were carried out in compliance with the IRB-approved protocol.

Blood was drawn into CPT tubes (Beckton-Dickinson # BD362761) and brought immediately to the pediatric rheumatology research laboratory. Sample processing was begun within an hour of obtaining the sample.

### CD4+ T cells

CD4+ T cells were purified from whole blood by negative selection using the StemSep™ Human CD4+ T Cell Enrichment Kit (STEMCELL Technologies Inc., Vancouver, Canada) as previously described ^46^.

### RNA purification and sequencing

Total RNA was extracted using TRIzol™ reagent and was further purified using RNeasy MiniElute Cleanup kit, including a DNase digest as previously described ^46^_._ RNA was quantified spectrophotometrically (Nanodrop, Thermo Scientific, Wilmington, DE) and assessed for quality by capillary gel electrophoresis (Agilent 2100 Bioanalyzer; Agilent Technologies, Inc., Palo Alto, CA). cDNA libraries were prepared for each sample using the Illumina TruSeq RNA Sample Preparation Kit following the manufacture’s recommended procedures. Libraries were sequenced using 100 base pair (bp) paired-end reads on the Illumina HiSeq 2500 platform. Library construction and RNASeq were performed at the University at Buffalo Genomics and Bioinformatics Core.

### ATAC-seq on CD4+ T cells

The assay for transposase-accessible chromatin sequencing (ATAC-seq) was carried out according to Buenrostro’s protocol ^47^_._ To prepare nuclei, 50,000 CD4+ T cells were spun at 500 × *g* for 5 min, followed by a wash using 50 μL of cold 1 × PBS and centrifugation at 500 × *g* for 5 min. Cells were lysed using cold lysis buffer (10 mM Tris–Cl, pH 7.4, 10 mM NaCl, 3 mM MgCl2 and 0.1% IGEPAL CA-630). Immediately after lysis, nuclei were spun at 500 × *g* for 10 min using a refrigerated centrifuge. Immediately following the nuclei prep, the pellet was resuspended in the transposase reaction mix (25 μL 2 × TD buffer, 2.5 μL Transposase (Illumina) and 22.5 μL of nuclease free water). The transposition reaction was carried out for 30 min at 37 °C. Directly following transposition the sample was purified using a Qiagen Minelute kit. Following purification, library fragments were amplified using 1 × NEBnext PCR master mix and 1.25 μM of PCR primer1 and Barcoded PCR Primer 2, using the following PCR conditions: 72 °C for 5 min, 98 °C for 30 s, followed by thermocycling at 98 °C for 10 s, 63 °C for 30 s and 72 °C for 1 min. To reduce GC and size bias in our PCR we monitored the PCR reaction using qPCR in order to stop amplification prior to saturation. To do this, the full libraries were amplified for 5 cycles, after 5 cycles , 5 μl of PCR reaction was added 10 μl of the PCR cocktail with Sybr Green at a final concentration of 0.6x. This reaction was run for 20 cycles, to determine the additional number of cycles needed for the remaining 45 μl reaction. To calculate the additional number of cycles needed, we plotted linear Rn versus cycle and to determine the cycle number that corresponded to one-third of the maximum fluorescent intensity. The libraries were purified using AMPure XP beads yielding a final library 17.5 μl. Sequencing was performed using 50 bp paired-end sequencing on the Illumina HiSeq 2500 platform at the University at Buffalo Genomics and Bioinformatics Core.

### ChIP-seq for CTCF

The ChIP assay was carried out using a kit from Diagenode (Denville, NJ). Briefly, Purified CD4+ T cells were fixed in 1% formaldehyde (J.T.Baker) in PBS for 10 min at room temperature, followed by 5 min blocking in 125 mM glycine. Cells were rinsed two times in ice-cold PBS and pelleted (500 g, 10 min, 4 °C). All fixed cell pellets were stored at − 80 °C. Approximately four million cells were resuspended in 0.5 ml of nuclei extraction buffer (5 mM PIPES pH 8; 85 mM KCl; 0.5% Igepal CA-630) supplemented with Protease Inhibitor Cocktail (Cell Signaling Technologies). Cell suspensions were sonicated using Bioruptor Plus (Diagenode) at low power with four cycles (15 s on and 30 s off). Samples were centrifuged (1000 *g*, 5 min, 4 °C) and washed once in 1 ml of the nuclei extraction buffer. Isolated nuclei were resuspended in the shearing buffer supplemented with Protease Inhibitor Cocktail and sonicated using Bioruptor Plus at high power with 25 cycles (30 s on and 30 s off; water temperature 4 °C) to reach a fragment size distribution of 100–700 bp. ChIP reactions were carried out in a final volume of 320 μl (70 ul washed Protein A-coated magnetic beads with antibody and 250 ul of sheared chromatin). 5 μl of sheared chromatin is stored at 4 °C as input sample. The antibody against CTCF was acquired from Diagenode (Cat No C15410210, Lot No A2359-00234p). After immunoprecipitation (IP) overnight at 4 °C, the beads were washed sequentially with different wash buffer, followed by incubation with elution buffer. Finally, the decross-linked DNA was resuspended in 25 ul buffer. Then DNA-sequencing was conducted using the Illumina HiSeq 2500 at the University at Buffalo Genomics and Bioinformatics Core.

### HiChIP for CTCF

CD4^+^ T cells were cross-linked using 1% formaldehyde for ten minutes at room temperature and then quenched by 125 mM glycine for five minutes. Four million cross-linked cells per sample were used in HiChIP assays as described in Mumbach et al., 2016 with some modifications ^36^. Briefly, nuclei were isolated and subjected to in situ digestion using MboI for 4 h at 37 °C. The cutting sites were then filled and labeled with dCTP, dGTP, dTTP and biotin-dATP. In situ proximity ligation was performed at room temperature overnight. After fragmentation of the DNA by sonication for two minutes, chromatin was immunoprecipitated using CTCF antibody (Cell Signaling, #3418S). DNA was eluted from the beads and purified by Zymo DNA Clean & Concentrator Column. Streptavidin M-280 Dynabeads were used to capture biotin-labeled DNA fragments. The sequencing libraries were generated on the streptavidin beads using the Nextera DNA Library Prep Kit.

### Sanger sequencing

DNA from whole blood was purified using DNeasy Blood & Tissue Kit (Qiagen, USA). DNA sequencing was performed in the Roswell Park Comprehension Cancer Institute.

### Data processing: aligning high-throughput sequencing reads to the reference genome

All RNA-seq, ATAC-seq and ChIP-seq read files, in FASTQ format, were aligned to the hg19 reference genome using bowtie2^48^. All of the files were run through bowtie2 using default parameters. RNA-seq and ATAC-seq data were run using paired-end mapping options (-1 and -2 options), while the CTCF ChIP-seq data was run using the unpaired options (-U option). HiChIP raw reads (fastq files) were aligned to hg19 human reference genome using HiC-Pro^49^, which was augmented with positions of Mbo1 restriction fragment cut sites for identifying alignment end sites. The resulting SAM files, outputted by HiC-Pro or bowtie2, were converted into BAM file formats using samtools ^50^.

### Data processing: RNA-seq analysis

After the data was aligned to the reference genome and converted into BAM format, the RNA-seq data was used to create a count table for differential analysis. For every gene, the number of RNA-seq reads for each sample mapping to that gene were counted using HT-Seq ^51^. HT-Seq was run using default parameters, using the GENCODE ^52^ version-19 gene annotations. This count table was then used as the input into the edgeR package for differential gene expression analysis ^53^.

Differential gene expression was identified using edgeR’s common dispersion protocol. A gene was considered expressed if at least five samples had a counts-per-million (CPM) value of at least 1. 14,357 genes were considered expressed under these conditions, in any of the three groups. edgeR was then run on the remaining list of expressed genes, first by calculating the normalization factors (calcNormFactors()), followed by calculating the common and then tag-wise dispersions (estimateCommonDisp(), estimateTagwiseDisp()), followed by an exact test and FDR correction (extactTest(), decideTestsDGE(,adjust.method = ”BH”,p.value = 0.05) to determine genes that were differentially expressed.

The volcano plots for differential gene expression were made by plotting the fold-change and p-value for every expressed gene, with genes whose p-value was ≤ 0.05 and whose log fold change was ≥  1 colored blue and those genes not matching these criteria colored red. The heat-map was created by identifying all genes that were considered differentially expressed in any of the three pairwise comparisons (4783 genes were considered), and clustering them based on their log CPM values into five clusters with k-means clustering. The individual samples were clustered with hierarchal clustering. Genes belonging to the five individual clusters were used for GO term analysis using GOrilla ^54^. GOrilla was run using the two gene set option, with the cluster genes as the input and the total list of expressed genes as the background set.

### Data processing: ChIP-seq and HiChIP analysis

After the ChIP-seq data was aligned to the reference genome and converted to BAM format, those BAM files were used as the input into MACS2 ^55^ for identifying CTCF binding sites. Each IP file had a matching input file that was used as inputs into MACS2. The q-value threshold for reporting peaks from MACS2 was set to 0.05 (option –q 0.05), which is the default value. Once peaks were called, those peaks were used as inputs into the DiffBind software^37^. For each sample, DiffBind takes the ChIP BAM files, the control input BAM files and the peaks from MACS2 as inputs. In total, DiffBind identified 61 CTCF binding sites that were considered differentially bound (FDR ≤ 0.1). The volcano plot showing the fold-change vs p-values (Fig. 2B) showed a possible artifact, evidenced by the diagonal line of points on the right side of the figure. Due to this, none of those sites were included in any further analysis and were not characterized with additional assays.

HiC-Pro alignments, in BAM format, were input into HiChipper^56^ using mostly default parameters. Mbo1 restriction fragment cut sites and CTCF peaks generated above, that were present in at least one sample, were input as anchor locating guides. Reads contributing to anchors were required to have a mapping quality of MAPQ ≥ 20.

Once the HiChIP loops had been identified, several post processing steps were conducted. We first sought to retain only those loops whose anchors were at valid, group-specific binding sites. To identify these we first took all ADT or HC CTCF peaks from MACS2 and determined consensus peaks for each group by finding sites that were considered peaks in at least 60% of samples in the group. Then every HiChIP loop, in each sample, was checked to see if both of its anchors were located at a consensus peak. If the loop was not anchored on both ends at a consensus peak, it was removed from further analysis.

### Data processing: ATAC-seq analysis

The aligned BAM files for the ATAC-seq data were first subjected to preprocessing steps to generate input files for peak calling. The unsorted BAM files were first sorted and then indexed using samtools (samtools sort followed by samtools index) ^50^. Bedtools ^57^ was used to convert the sorted and indexed BAM files into a bed file (bedtools bamtobed) and these bed files were then used to create a genome wide bigwig file of read coverage, first with bedtools and then with UCSC’s bedGraphToBigWig tools (bedtools genomecov –bga followed by bedGraphToBigWig). The sorted BAM files, the BAM index files and these BigWig files were used as inputs into the ATAC-seq peak calling algorithm, HMMRATAC ^58^. Additionally, a genome size file was downloaded from UCSC’s genome browser as was a list of blacklisted sites for the hg19 genome annotation (wgEncodeDacMapabilityConsensusExcludable.bed with the –e option in HMMRATAC) ^59,60^. HMMRATAC was run with the upper and lower range settings for finding candidate peaks to build the model, set to 20 and 10 respectively (-u 20 and –l 10 options). For each sample, HMMRATAC outputted a peak file, with the locations of every accessible region, the top 100,000 of which were kept for further analysis. Consistent peaks were considered to be genomic regions that were identified as a peak in at least 60% of the group’s samples. A total of 108,596 consistent HC peaks, 223,941 consistent ADT peaks and 103,612 consistent CRM peaks were identified. GO term enrichment for any group of peaks was determined by using GREAT^61^, with default settings.

### Data processing: identifying linkage disequilibrium blocks for JIA risk loci

Zhu et al. had previously identified the linkage disequilibrium blocks for 35 JIA risk associated SNPs ^9^. Additional risk loci have been identified since and we aimed to incorporate those into our analysis^7,40^. Because the method used to identify the LD blocks from Zhu et al. is no longer maintained, we processed all JIA risk SNPs using SNiPA, using the same settings for each SNP ^62^. Using the “Block Annotation” tool from the SNiPA suite, we ran each SNP using the GRCh37 genome assembly, the 1000 Genomes, Phase 3 v5 variant set, set the population to European and the genome annotation to Ensembl 87. Each block used an r^2^ of 0.8 to identify linkage disequilibrium.

### Data processing: identifying interaction domains around JIA risk loci

ATAC-seq, ChIP-seq and RNA-seq data was converted from raw BAM files into BigWig files and then merged together and averaged. HiChIP loops were converted into a paired end BED format and filtered to keep only those loops with at least 2 reads supporting it. All of this data, along with a BED file of JIA risk associated LD blocks from Zhu et al. ^9^, was then uploaded onto the WashU genome browser ^63^ for visualization. Starting with each LD block, we determined whether the LD region contained a CTCF loop anchor. If not, then the LD block was considered to be inactive in CD4+ T cells and was excluded from further analysis. If the LD blocks that contained one or more CTCF loop anchors, we then determined all genomic regions that physically interacted with the block. We then determined all regions that interacted with those anchors, and so forth, until we had identified all regions that could come into close physically proximity to the LD block. An example of a positive region is shown in Fig. 3.

### Data processing: determining enhancer-TSS links within regulatory hubs

Having defined all regulatory hubs that are anchored on a JIA risk associated locus, we sought to determine which enhancers within a hub controlled the expression of which genes. Using HT-seq ^51^, we counted the number of RNA-seq reads that mapped to each gene for each sample and the number of ATAC-seq reads that mapped to each identified peak for each matched sample, i.e. those samples where there was both RNA-seq and ATAC-seq data. We then converted those read counts into log(Counts per million) using edgeR. Then, for each regulatory hub, we constructed a matrix of the log(CPM) for RNA-seq reads for each gene in the hub and log(CPM) for ATAC-seq reads for each peak in the hub. We then perform feature selection with Boruta^64^, modeling the expression of every gene (Y) as a function of the expression level of all other genes in the hub (T) as well as the accessibility (A) of all ATAC-seq peaks (P) within the hub.

$${Y}_{i}=\sum_{1}^{T}{Y}_{t}+\sum_{1}^{P}{A}_{p}$$

Our modeling approach is similar to recent studies that have used the level of accessibility of regulatory elements to predict enhancer-promoter interactions^65,66^. Because of the stochastic nature of the Boruta algorithm, we ran it 50 times per gene and only retained features that were considered significant in at least 95% of the runs. Finally, we focused on those genes whose features were located within the JIA risk associated LD blocks.

## Supplementary Information

Supplementary Figure 1.

Supplementary Figure 2.

Supplementary Table 1.

Supplementary Table 2.

Supplementary Table 3.

Supplementary Table 4.

Supplementary Table 5.

Supplementary Table 6.

Supplementary Table 7.

Supplementary Legends.
