# Supplementary figures and images for: CD4+ T cells from children with active juvenile idiopathic arthritis show altered chromatin features associated with transcriptional abnormalities
Source: Sci Rep. 2021 Feb 17;11:4011. doi: 10.1038/s41598-021-82989-5 (PMC7889855; doi:10.1038/s41598-021-82989-5)

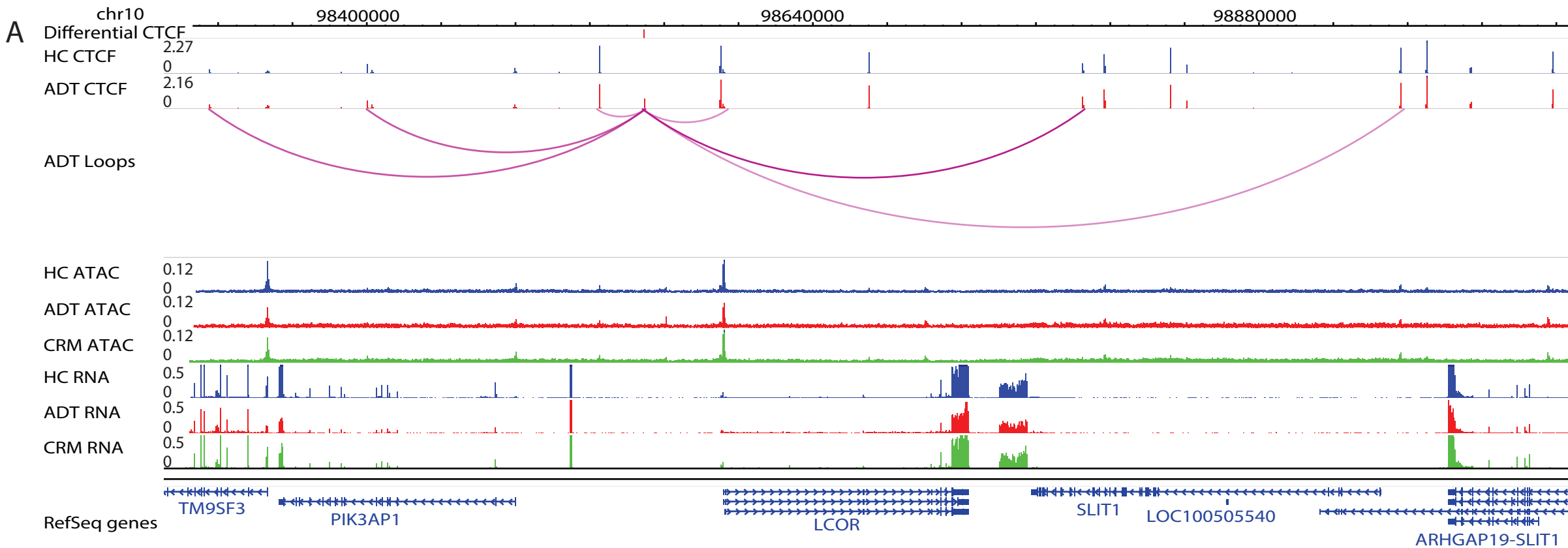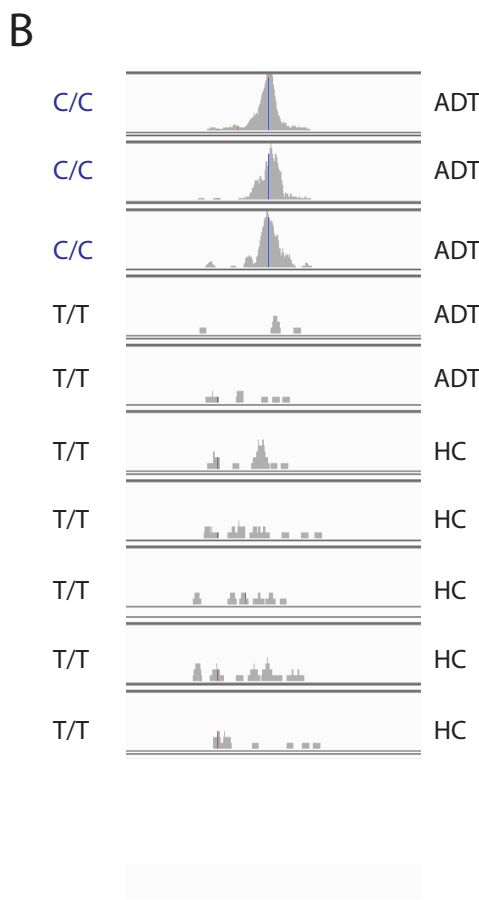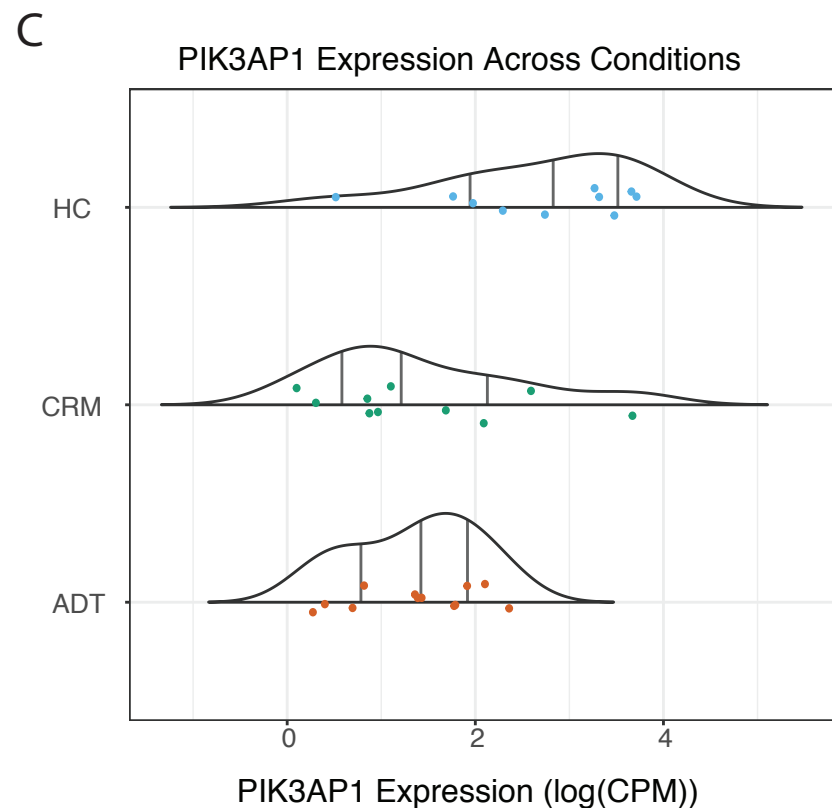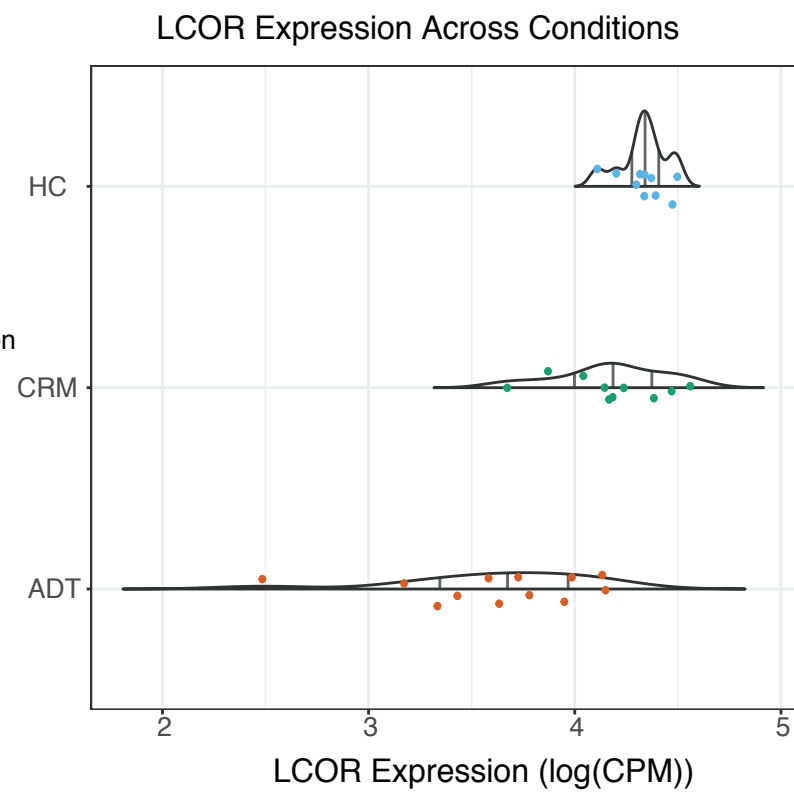

Supplement: Supplementary file 1 — Supplementary Figure 1. [file 41598_2021_82989_MOESM1_ESM.pdf]

A

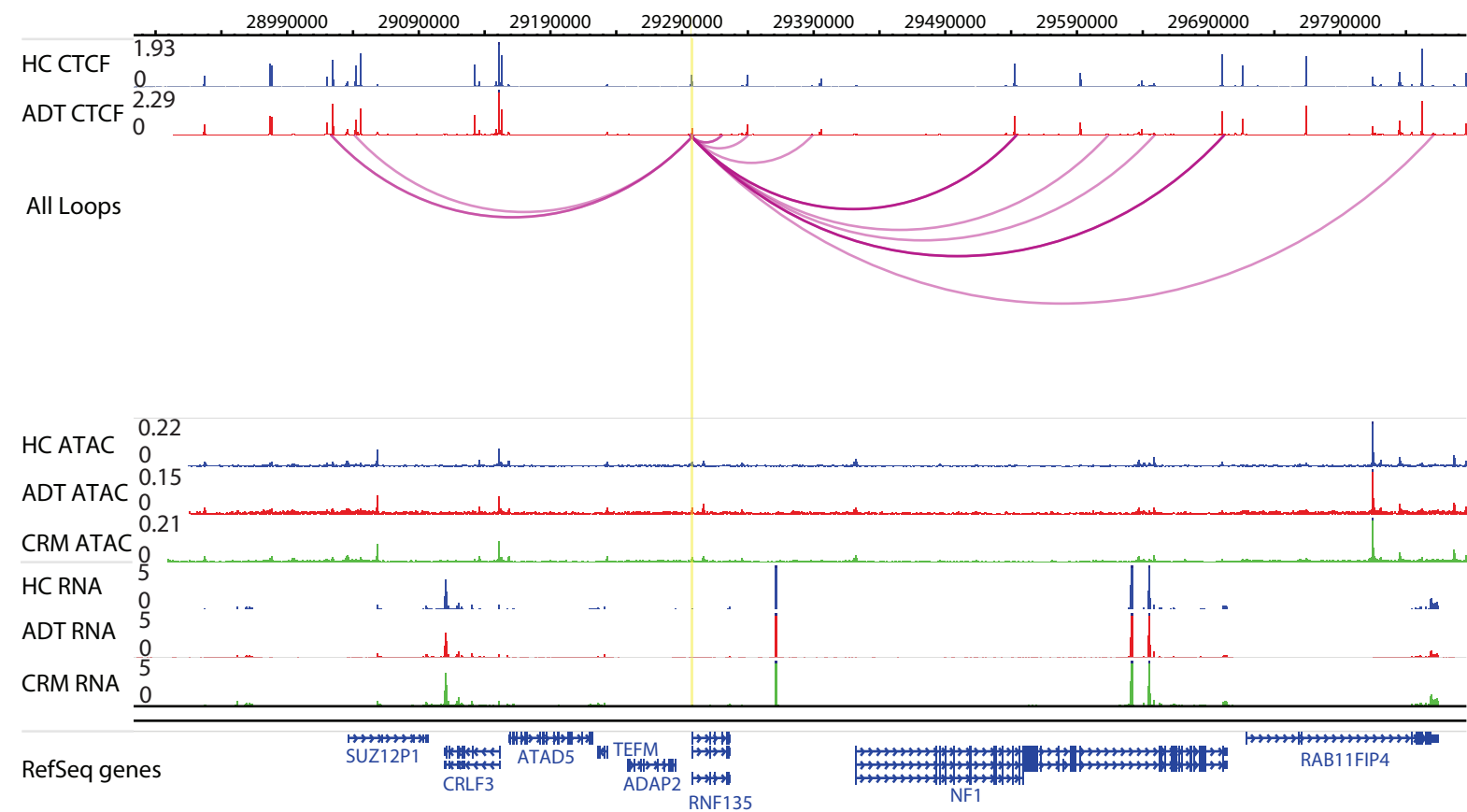

B

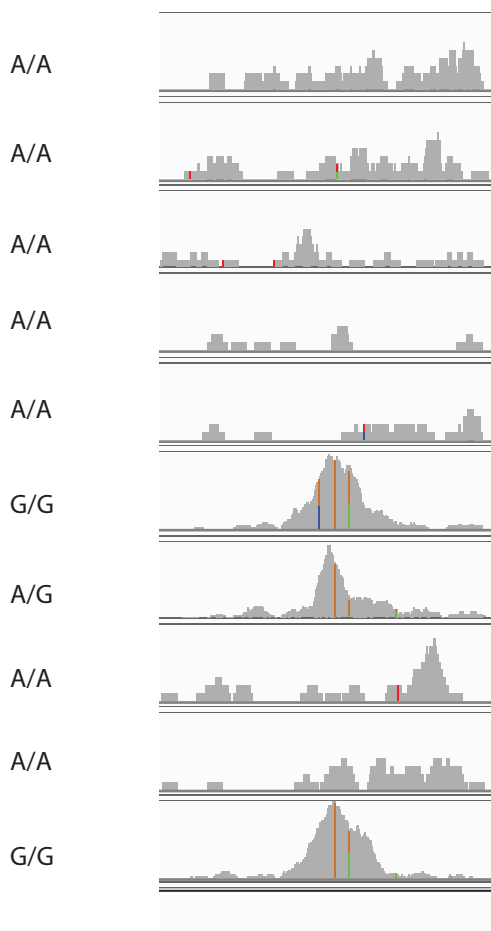

C

### RAB11FIP4 Expression Across Conditions

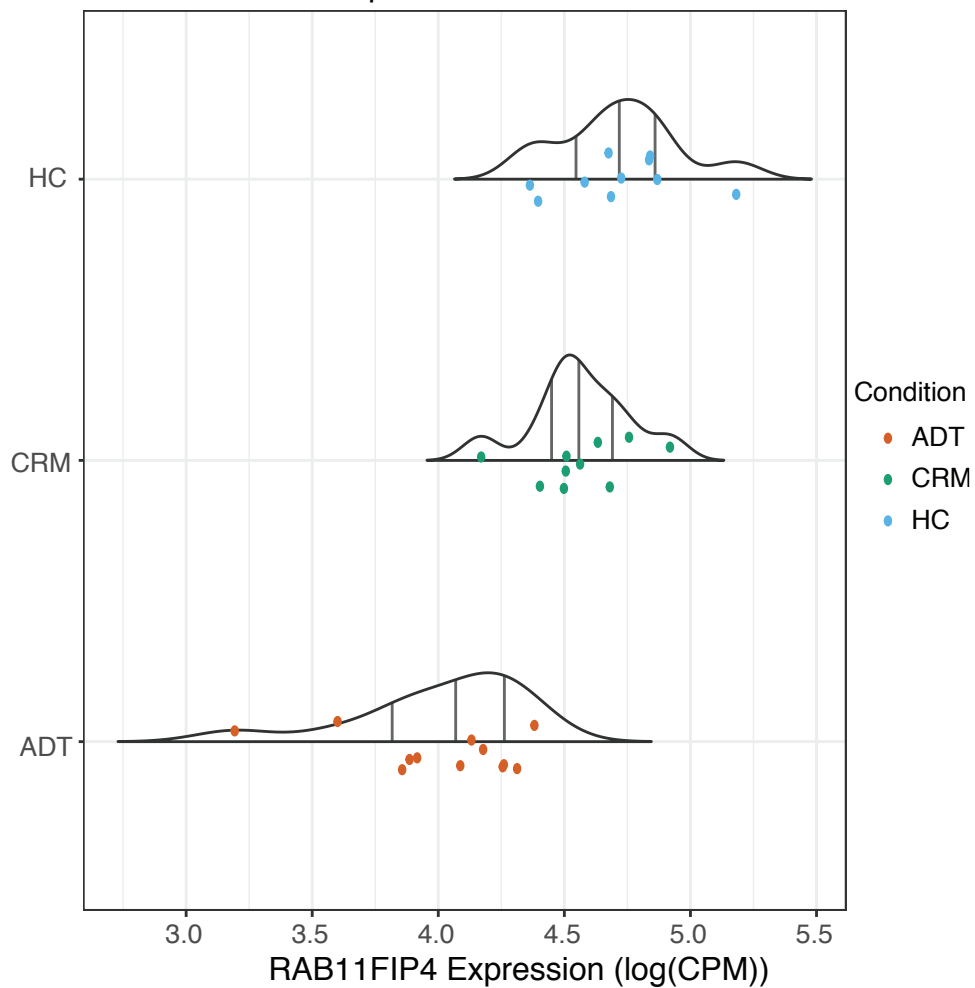

Supplement: Supplementary file 2 — Supplementary Figure 2. [file 41598_2021_82989_MOESM2_ESM.pdf]
