# Supplementary material for: CD4+ T cells from children with active juvenile idiopathic arthritis show altered chromatin features associated with transcriptional abnormalities
Source: Sci Rep. 2021 Feb 17;11:4011. doi: 10.1038/s41598-021-82989-5 (PMC7889855; doi:10.1038/s41598-021-82989-5)
Supplement: Supplementary file 3 — Supplementary Table 1. [file 41598_2021_82989_MOESM3_ESM.docx]

**Table S1**

Differentially expressed genes between ADT and HC, whose log10(Fold-change) was at least 1 and whose FDR was less than or equal to 0.05.

| **Gene Names** | **Log(Fold-change)** | **Log(CPM)** | **P-Value** |
| --- | --- | --- | --- |
| FGR | 1.280258 | 5.94614 | 0.00183 |
| CFH | 1.091301 | 2.124987 | 0.006197 |
| PLXND1 | 1.01822 | 4.826442 | 2.06E-06 |
| PDK4 | 2.160535 | 1.100545 | 0.006497 |
| GAS7 | 1.65792 | 4.644465 | 3.67E-07 |
| PLEKHG6 | -1.52295 | 0.939083 | 8.95E-05 |
| RPS20 | -1.2426 | 8.865639 | 1.45E-30 |
| ANK1 | 1.084676 | 4.097506 | 2.91E-08 |
| FAM65C | -1.12651 | 2.485922 | 1.10E-06 |
| PRSS8 | -1.97109 | 0.027264 | 3.04E-05 |
| ATP9A | 1.199726 | 1.6861 | 0.002949 |
| TMCC3 | 1.442767 | 1.713304 | 0.000913 |
| SEZ6 | -1.49197 | 0.176442 | 0.007972 |
| PDE4A | 1.257007 | 3.121314 | 4.16E-08 |
| KIF26A | 1.595051 | 0.737954 | 0.005487 |
| ST6GALNAC1 | -1.10234 | 3.881503 | 3.44E-05 |
| RPL31 | -1.08758 | 7.08541 | 9.32E-19 |
| VASH1 | 1.039595 | 4.750216 | 4.12E-10 |
| CYBRD1 | 1.320112 | 2.430308 | 0.003053 |
| RHOBTB1 | 2.288124 | 0.088338 | 0.000529 |
| FOSL2 | 1.296518 | 5.856492 | 1.06E-05 |
| GNAO1 | 1.0651 | 2.173375 | 0.000771 |
| OTUB2 | -1.31646 | 1.874111 | 2.60E-05 |
| FCGBP | 1.182856 | 4.561942 | 6.37E-06 |
| DFNB31 | 1.127774 | 2.757922 | 1.40E-05 |
| CDHR5 | -1.49382 | 1.339418 | 0.000128 |
| SERPIND1 | -2.07825 | 0.062097 | 0.000116 |
| SOX10 | -1.87578 | 0.201082 | 0.001178 |
| MLC1 | 1.122859 | 2.50561 | 0.00055 |
| SIRPB1 | 1.287378 | 3.844218 | 0.000246 |
| CACNA1F | 1.055863 | 1.308687 | 3.72E-05 |
| ESRP2 | -1.08889 | 2.029291 | 4.50E-05 |
| FAM189A1 | -1.61638 | -0.22834 | 0.008931 |
| DMXL2 | 1.289098 | 4.331566 | 0.000208 |
| PEX11G | -1.23278 | 2.150584 | 2.95E-05 |
| LILRB1 | 1.242263 | 3.368273 | 0.002925 |
| RPS16 | -1.02646 | 8.232539 | 2.53E-24 |
| PLA2G4C | 1.749874 | 0.849828 | 0.00953 |
| FSD1L | 1.162872 | 1.820568 | 2.29E-05 |
| RLN2 | -1.01036 | 1.409152 | 0.010156 |
| KCNT1 | 1.020202 | 0.722405 | 0.007923 |
| RPL19 | -1.07184 | 8.719396 | 7.70E-29 |
| RASD1 | 1.342115 | 1.685606 | 0.002857 |
| RPL34 | -1.26882 | 6.566094 | 1.29E-19 |
| B3GAT1 | 1.833816 | 1.311449 | 9.64E-05 |
| FOLR3 | 2.962982 | 0.805933 | 0.006994 |
| TENC1 | -1.21071 | 2.449721 | 1.89E-07 |
| GLI1 | -1.15587 | 2.282645 | 4.30E-05 |
| C12orf57 | -1.05538 | 7.084379 | 1.25E-19 |
| RPS12 | -1.27412 | 6.685736 | 8.06E-23 |
| GUCA1B | 1.106414 | 0.977221 | 0.002852 |
| COX7A2 | -1.2717 | 4.989111 | 1.41E-21 |
| ENPP5 | 1.264771 | 1.194561 | 0.009248 |
| HAVCR1 | 1.243835 | 0.528725 | 0.009292 |
| STEAP3 | 1.61815 | 0.768624 | 0.009608 |
| TFCP2L1 | 1.770738 | 0.651567 | 0.004853 |
| SF3B14 | -1.01777 | 4.597586 | 1.12E-14 |
| KCNJ13 | -1.86772 | -0.01759 | 0.002533 |
| GNLY | 1.363558 | 6.960295 | 0.000512 |
| IL18RAP | 1.26434 | 4.337525 | 3.60E-06 |
| C1orf21 | 1.274262 | 2.811845 | 8.32E-05 |
| TSPAN1 | -2.32798 | -0.45451 | 0.006624 |
| MUC5B | -1.69553 | -0.07521 | 0.001605 |
| B4GALT6 | 1.362158 | 1.11517 | 0.004277 |
| PPL | -1.3746 | 0.408155 | 0.00691 |
| NDUFB3 | -1.17113 | 4.145691 | 3.11E-14 |
| PIGZ | 1.170095 | 1.067701 | 0.005984 |
| CSF3R | 1.719961 | 5.235053 | 0.00539 |
| ARAP3 | 1.013162 | 2.329504 | 0.000474 |
| EGR1 | 1.951303 | 5.3184 | 0.007549 |
| CHRNA2 | -1.7213 | 0.20589 | 0.000111 |
| TNFRSF8 | 1.245064 | 0.920741 | 0.008343 |
| ABCC11 | 1.006403 | 2.273306 | 4.73E-07 |
| KIAA0087 | 2.350715 | 0.338925 | 0.002378 |
| NR4A1 | 2.171505 | 4.933675 | 0.004207 |
| LRP1 | 1.631925 | 5.138274 | 4.46E-05 |
| SDC4 | 1.035283 | 0.805409 | 0.000415 |
| RP11-663P9.2 | -1.02581 | 5.353392 | 8.01E-12 |
| HIST1H1D | -1.34624 | 2.272291 | 8.11E-05 |
| CDKN1A | 1.292912 | 3.43614 | 0.008352 |
| RPL23 | -1.30556 | 6.890703 | 5.74E-25 |
| SNRPD2 | -1.23016 | 6.819579 | 4.05E-27 |
| CD93 | 1.260416 | 5.080051 | 0.000434 |
| COX6B1 | -1.26969 | 4.706806 | 1.54E-16 |
| COX7C | -1.21462 | 7.150977 | 4.17E-32 |
| SIGLEC9 | 1.451929 | 2.392303 | 0.005971 |
| LPPR3 | -1.69841 | 0.678024 | 3.98E-06 |
| PVRL2 | 1.760529 | 0.102099 | 0.003286 |
| RPL36 | -1.16169 | 6.945962 | 3.39E-33 |
| KCNC3 | 1.120137 | 2.848526 | 0.000776 |
| RPL27 | -1.32832 | 8.333772 | 2.20E-29 |
| CA6 | -1.0074 | 4.091331 | 4.57E-05 |
| ITGB4 | -1.30993 | 0.580169 | 0.000802 |
| SLC52A1 | -1.21463 | 0.343609 | 0.004105 |
| LGR6 | 1.783309 | 0.534772 | 0.001672 |
| PRAM1 | 1.430639 | 3.690102 | 0.000325 |
| CTIF | 1.387247 | 2.107344 | 5.68E-06 |
| MRPS36 | -1.01681 | 2.638344 | 7.86E-06 |
| SPIRE1 | 1.521654 | 0.801294 | 0.004094 |
| FST | -3.10024 | 2.100517 | 2.09E-05 |
| EMP1 | 1.171062 | 1.183208 | 0.008435 |
| TIMM10 | -1.01432 | 3.541581 | 5.29E-07 |
| KLB | -1.83958 | 0.375769 | 0.00035 |
| CCNJL | 1.798029 | 0.035268 | 0.007883 |
| SPRY2 | 1.554339 | 0.072376 | 0.008266 |
| NACAD | -2.57118 | 0.621674 | 9.45E-07 |
| WDR38 | -1.54492 | 1.014078 | 0.000457 |
| RPL35 | -1.07888 | 7.575652 | 2.11E-27 |
| PGBD1 | 1.097653 | 1.818003 | 0.000207 |
| THBS1 | 2.167222 | 1.983153 | 5.64E-05 |
| RP11-122C9.1 | -1.0653 | 7.213964 | 1.86E-16 |
| CYP1B1 | 2.06018 | 2.581255 | 0.00734 |
| RPS24 | -1.20807 | 8.220464 | 2.02E-26 |
| FGD4 | 1.60229 | 1.869561 | 0.002398 |
| CELA1 | -1.58093 | 1.717168 | 7.11E-06 |
| SLC24A4 | 2.429124 | 2.329072 | 1.86E-05 |
| SRP14 | -1.00516 | 6.952358 | 1.88E-24 |
| ITGAX | 1.338918 | 5.176169 | 0.000575 |
| RNF165 | 1.357755 | 2.193498 | 2.34E-05 |
| TRPM2 | 1.561163 | 3.093508 | 7.25E-05 |
| SIGLEC10 | 1.277934 | 3.02046 | 0.00439 |
| ACPT | -1.07255 | 0.516042 | 0.010072 |
| RPS11 | -1.07865 | 8.543885 | 7.73E-26 |
| RPL11 | -1.08771 | 9.282055 | 2.64E-26 |
| RPS8 | -1.06655 | 8.843256 | 4.00E-28 |
| SOX13 | 1.6541 | 2.362861 | 7.25E-10 |
| RHOB | 1.356852 | 5.973819 | 0.006507 |
| RPS27A | -1.1769 | 8.024038 | 4.29E-29 |
| SLC4A10 | 1.110169 | 3.226736 | 0.003206 |
| ITGA9 | 1.796496 | -0.00472 | 0.009991 |
| RPL32 | -1.13561 | 9.033503 | 6.54E-28 |
| PHLDB2 | 1.161314 | 2.489717 | 5.29E-06 |
| MED12L | 1.200772 | 1.061712 | 0.007802 |
| SCD5 | 1.173533 | 0.890932 | 0.004828 |
| GFOD1 | 1.382177 | 3.552273 | 1.85E-07 |
| CREB5 | 1.564922 | 2.554042 | 0.007959 |
| AC004453.8 | -1.19852 | 10.38394 | 5.37E-25 |
| FRMPD3 | 2.524929 | 0.599428 | 0.000289 |
| DOCK5 | 1.374305 | 2.987531 | 0.001048 |
| ADIRF | 1.020691 | 0.965912 | 0.006346 |
| ADAM12 | 1.086223 | 2.680341 | 0.001689 |
| ADM | 2.166555 | 1.403531 | 0.005355 |
| NCAM1 | 1.504923 | 2.109087 | 0.005796 |
| FAU | -1.02913 | 7.398261 | 3.22E-26 |
| KLRF1 | 1.23848 | 2.801488 | 0.00493 |
| LATS2 | 1.476333 | 2.871146 | 5.03E-07 |
| TIMM8B | -1.05982 | 4.275043 | 3.36E-09 |
| C12orf45 | -1.03233 | 2.943742 | 1.20E-06 |
| DLG5 | 1.147253 | 2.465245 | 0.001126 |
| HHEX | 1.045352 | 3.117539 | 0.008063 |
| LRRK1 | 1.04451 | 2.841855 | 0.00344 |
| LY96 | -1.22952 | 1.865645 | 1.25E-05 |
| PPM1J | 1.911516 | 0.297909 | 0.001782 |
| PIK3AP1 | 1.052611 | 4.591622 | 0.000561 |
| C14orf2 | -1.00859 | 5.409666 | 8.00E-18 |
| RPL30 | -1.08528 | 9.198053 | 2.15E-26 |
| B3GNT7 | 1.474209 | 1.47647 | 0.00083 |
| AGAP1 | 1.307441 | 2.393615 | 0.000207 |
| CPA5 | -1.42233 | 2.013775 | 0.002806 |
| SPON2 | 1.043449 | 4.494937 | 2.58E-06 |
| TNFRSF13C | 1.166043 | 1.749668 | 1.17E-05 |
| PTGIR | 1.316293 | 3.537691 | 8.17E-05 |
| VAV2 | 1.192981 | 1.269391 | 0.007897 |
| TLCD1 | -1.35528 | 0.878461 | 0.000805 |
| CHRNB2 | -1.70499 | 1.570071 | 1.66E-05 |
| GBAP1 | 1.487184 | 3.193027 | 0.000459 |
| SCGB3A1 | -4.74628 | 0.606075 | 3.49E-05 |
| ZNF385A | 1.488472 | 3.866079 | 0.003144 |
| RPL26 | -1.22313 | 7.374054 | 4.58E-26 |
| RPL29 | -1.19815 | 9.665546 | 1.35E-20 |
| WNT4 | -2.03892 | 0.8625 | 1.49E-09 |
| KCNJ9 | -1.72011 | 0.029194 | 0.000206 |
| SLC22A15 | 1.477934 | 1.34871 | 0.004722 |
| PROK2 | 1.714488 | 0.645116 | 0.000797 |
| FCRL1 | 1.292368 | 4.345746 | 0.001687 |
| GUCY1A3 | 1.726112 | 0.783738 | 0.002196 |
| NDUFS4 | -1.10193 | 3.341785 | 1.10E-07 |
| KLKB1 | 1.516458 | 1.243574 | 0.00061 |
| SHROOM1 | 2.572811 | 0.297812 | 0.000907 |
| RPS14 | -1.17041 | 8.659428 | 1.18E-27 |
| COX6C | -1.25677 | 5.960127 | 3.99E-28 |
| RPL36AL | -1.06406 | 8.235393 | 3.61E-15 |
| RPL27A | -1.00361 | 8.051987 | 6.56E-17 |
| NGFRAP1 | -1.05831 | 5.006443 | 2.06E-08 |
| MS4A14 | 1.630168 | 1.429653 | 0.009279 |
| PIP5KL1 | -1.16017 | 0.778829 | 0.005918 |
| ATP5L | -1.04407 | 6.308571 | 7.00E-25 |
| FAM129C | 1.281704 | 3.779649 | 0.001816 |
| TTYH1 | -1.03042 | 1.097796 | 0.005191 |
| SLC43A2 | 1.003591 | 3.847533 | 0.003516 |
| RCOR2 | -2.17434 | 0.065462 | 0.000198 |
| RAB26 | -1.65523 | 0.121447 | 0.00012 |
| PTGDR | 1.498574 | 2.892886 | 6.55E-09 |
| HIST1H1E | -1.63481 | 1.224687 | 5.56E-05 |
| DEGS2 | -2.42818 | 1.684024 | 2.34E-09 |
| SCN11A | -1.71154 | -0.35483 | 0.003411 |
| NDUFS5 | -1.1628 | 5.453419 | 2.08E-19 |
| MN1 | 1.915655 | 0.267265 | 0.000642 |
| RP11-220D10.1 | -1.17922 | 7.216612 | 1.20E-34 |
| CD52 | -1.23663 | 9.718724 | 2.64E-27 |
| ZEB2 | 1.30326 | 5.148013 | 0.002702 |
| ITGAM | 1.103725 | 5.116135 | 0.00011 |
| SMAD1 | 1.514149 | 1.522934 | 0.004742 |
| CDH2 | -2.19282 | -0.39116 | 0.005443 |
| GPR27 | 2.354976 | 1.541444 | 1.49E-05 |
| PDGFD | 1.498703 | 1.4702 | 0.000137 |
| LRRC8E | -1.30685 | -0.10784 | 0.007304 |
| INSR | 1.000949 | 2.228704 | 0.004544 |
| RP11-1055B8.7 | 2.062685 | 1.612576 | 2.87E-05 |
| NMUR1 | 2.030436 | 2.4333 | 3.86E-09 |
| LGALS4 | 1.375946 | 1.759231 | 0.007288 |
| RPS21 | -1.01393 | 3.771179 | 3.57E-09 |
| GPR22 | -2.59679 | -0.75353 | 0.000993 |
| C1QTNF4 | 1.515309 | -0.09214 | 0.007412 |
| MYEOV2 | -1.02916 | 4.147522 | 2.68E-12 |
| PRSS30P | 1.936889 | 0.24453 | 0.000553 |
| ZBTB8OSP2 | -1.3061 | 3.76658 | 4.14E-09 |
| RPL38 | -1.10716 | 5.622509 | 1.63E-19 |
| EGFL7 | 1.920193 | -0.01838 | 0.001898 |
| HSPA6 | 3.358258 | 2.59639 | 3.98E-05 |
| XCR1 | 1.024956 | 2.543797 | 0.00649 |
| C9orf131 | -1.18808 | 0.983231 | 0.000219 |
| CD34 | 1.980565 | 1.781384 | 5.15E-05 |
| ADCY6 | 2.011593 | -0.2206 | 0.000863 |
| CMKLR1 | 1.556284 | 2.331399 | 0.000424 |
| LINC00116 | -1.15132 | 3.831225 | 2.59E-11 |
| GPX2 | -1.35485 | 1.174681 | 0.000574 |
| RPL37AP8 | -1.24387 | 1.952382 | 0.000221 |
| AC110781.3 | -1.22823 | 0.961331 | 0.003527 |
| AC024940.1 | -1.52552 | 0.970148 | 0.001012 |
| RPLP2 | -1.00206 | 7.132345 | 4.46E-21 |
| JUN | 1.47722 | 8.527926 | 0.009112 |
| RP11-474P12.3 | -1.08152 | 1.914346 | 9.14E-06 |
| ODF3 | -2.39901 | 0.160517 | 1.52E-05 |
| DDC8 | -1.69557 | -0.27671 | 0.003045 |
| RPS3AP5 | -1.40549 | 1.205445 | 2.01E-05 |
| COX14 | -1.01951 | 4.901993 | 1.02E-14 |
| MCMDC2 | -1.0964 | 4.299203 | 1.86E-16 |
| HLA-DQB1 | 1.308165 | 5.892435 | 0.007167 |
| C1orf200 | -1.67281 | 1.912126 | 3.75E-06 |
| TMEM150B | 2.402636 | -0.3458 | 0.002394 |
| MYLPF | -1.02682 | 0.801281 | 0.001661 |
| RP1-278E11.3 | -1.32072 | 9.898059 | 1.41E-31 |
| ATP5EP2 | -1.36971 | 8.438393 | 5.88E-25 |
| SHISA2 | -1.00519 | 0.280818 | 0.006924 |
| CXCR2 | 2.083366 | 0.497986 | 0.00487 |
| FCRL6 | 1.542327 | 2.832288 | 4.27E-07 |
| F2R | 1.26155 | 3.860553 | 2.01E-07 |
| RPL24P4 | -1.02437 | 9.303923 | 4.20E-18 |
| SNRPE | -1.08268 | 4.011411 | 6.03E-12 |
| CHST15 | 1.15276 | 3.933942 | 0.005207 |
| CRIP1P4 | -1.34174 | 4.813101 | 5.39E-15 |
| NXPH3 | -2.59123 | -0.0831 | 9.21E-06 |
| RPL35A | -1.10081 | 8.307497 | 4.61E-28 |
| SLC8A1 | 1.590794 | 2.341295 | 0.003571 |
| RP11-556K13.1 | -1.56979 | 0.52458 | 0.000603 |
| RPS7P1 | -1.00584 | 9.043883 | 1.16E-16 |
| ASCL2 | 1.319129 | 1.256826 | 0.006426 |
| UQCR10 | -1.06074 | 5.608157 | 6.76E-20 |
| RP11-44M6.3 | -1.37629 | 0.198666 | 0.001059 |
| BEX5 | -1.03746 | 2.132028 | 0.001011 |
| FAM110C | 1.276249 | 0.389042 | 0.005713 |
| NDUFA12 | -1.38544 | 4.89216 | 2.30E-25 |
| MUC6 | -2.34787 | 1.865878 | 5.47E-11 |
| TNFAIP2 | 1.582125 | 5.808044 | 0.007725 |
| WBP5 | -1.05469 | 0.805536 | 0.005015 |
| CTD-2287O16.1 | -1.35799 | 9.058125 | 3.25E-23 |
| NAT8L | 1.925105 | -0.04164 | 0.00328 |
| RPL12P4 | -1.26679 | 6.22683 | 2.81E-30 |
| KDM4D | 1.047792 | 0.020222 | 0.007421 |
| RXRA | 1.127993 | 4.803518 | 0.000129 |
| CD300E | 1.702515 | 4.565726 | 0.007945 |
| ISG15 | -1.17694 | 5.876478 | 0.000103 |
| COMMD6 | -1.2418 | 6.333021 | 1.96E-24 |
| TMEM198 | 1.186806 | 0.646691 | 0.003965 |
| BEND4 | 1.007741 | 1.78198 | 0.008736 |
| RPL10AP2 | -1.18917 | 3.115917 | 6.57E-09 |
| HIST1H4D | -1.6931 | -0.43576 | 0.009007 |
| PAX5 | 1.389038 | 3.563165 | 0.002946 |
| KIF19 | 1.173013 | 0.765892 | 0.001609 |
| PLXNB2 | 1.371501 | 4.666681 | 0.002621 |
| TOMM7 | -1.25266 | 7.501309 | 5.44E-22 |
| DAPK1 | 1.0732 | 3.386835 | 0.005776 |
| CHRNG | -1.30759 | 0.571739 | 0.001793 |
| DTHD1 | 1.346591 | 2.545518 | 0.002484 |
| HIST1H4C | -1.4432 | 0.012987 | 0.003819 |
| SRC | 1.21861 | 2.928655 | 0.000141 |
| MPEG1 | 1.18863 | 7.143546 | 0.009799 |
| ZNF460 | -1.34375 | 3.940331 | 3.07E-17 |
| RPL37A | -1.16985 | 6.856979 | 3.64E-19 |
| FAM49A | 1.27759 | 3.442372 | 0.001543 |
| RPS4X | -1.00461 | 9.636159 | 3.41E-22 |
| TOR4A | 1.316252 | 3.428381 | 3.08E-09 |
| HLA-DRB5 | 3.059923 | 2.427749 | 0.001582 |
| SH2D1B | 1.483773 | 3.178076 | 0.000546 |
| SMIM11P1 | -1.02472 | 1.366952 | 0.00117 |
| RP5-857K21.11 | -1.57637 | 1.15834 | 0.00116 |
| MT-ND3 | -1.09194 | 10.24573 | 3.75E-12 |
| CES1 | 2.661462 | -0.36105 | 0.005189 |
| MIR342 | -1.25504 | 1.714633 | 6.75E-05 |
| RNU5A-1 | -1.04071 | 3.091242 | 0.001861 |
| SNORA73B | -1.75938 | 2.906441 | 3.08E-12 |
| RNU6-181P | 1.508292 | -0.37458 | 0.002836 |
| U3 | -1.43656 | 0.055093 | 0.004803 |
| RNU4-1 | -1.64626 | 0.790078 | 0.000107 |
| RN7SK | -2.66603 | 5.79661 | 4.41E-47 |
| Y | 1.312556 | 0.715845 | 0.000292 |
| RNU4-2 | -2.13561 | 3.677145 | 1.47E-14 |
| AP001007.1 | 1.79298 | 1.128296 | 0.000491 |
| OR1F2P | -1.28823 | 0.737227 | 0.003578 |
| FCGR3A | 1.188656 | 4.066201 | 0.003507 |
| PFN1P2 | 1.698364 | -0.20951 | 0.001647 |
| HSPA1B | 3.26912 | 6.112174 | 1.05E-06 |
| HSPA1A | 1.340776 | 5.441886 | 0.000123 |
| C6orf25 | 1.708404 | 2.694876 | 0.009514 |
| MCCD1 | -1.4345 | -0.40096 | 0.005434 |
| AC016586.1 | -1.33096 | 1.23546 | 0.000131 |
| MUC12 | -1.51769 | 0.255925 | 0.000116 |
| GPR56 | 1.601694 | 4.535899 | 2.19E-06 |
| TMEM256 | -1.08778 | 3.134439 | 2.14E-07 |
| SNORA54 | -2.13547 | 0.059786 | 0.001053 |
| RNVU1-15 | -2.07796 | -0.00674 | 0.000314 |
| SNORD15B | -1.38769 | 1.269525 | 0.000589 |
| RNVU1-14 | -1.44381 | 2.832978 | 1.72E-06 |
| MIR762 | 2.461527 | 1.107424 | 1.67E-07 |
| IGKV4-1 | -2.08236 | 7.364954 | 0.004287 |
| IGLV1-51 | -2.12119 | 2.019306 | 0.004008 |
| IGLV1-47 | -2.32315 | 3.514978 | 3.32E-05 |
| IGLV7-43 | -2.01434 | 3.904306 | 0.001943 |
| IGLV2-8 | -1.39866 | 4.337963 | 0.007945 |
| TRBV11-1 | -1.43565 | 1.433075 | 0.001383 |
| TRBV25-1 | -1.29934 | 2.678732 | 1.45E-05 |
| TRAV39 | -1.00381 | 2.045461 | 0.00174 |
| TRAJ38 | -1.23193 | 0.620524 | 0.008316 |
| TRAJ35 | -1.3684 | 0.152253 | 0.00868 |
| TRAJ29 | -1.6965 | 0.486725 | 9.33E-08 |
| TRAJ26 | -1.24812 | 0.400665 | 0.009304 |
| TRAJ23 | -1.43032 | 0.407142 | 0.002945 |
| IGHV6-1 | -1.68865 | 3.325802 | 0.006166 |
| IGHV2-26 | 2.969185 | 2.526615 | 0.003493 |
| IGHV3-66 | -3.15801 | 2.917119 | 0.009121 |
| U3 | -1.82191 | 1.280932 | 9.56E-05 |
| SNORD17 | -1.6382 | 1.597946 | 2.18E-09 |
| SNORA12 | -1.52966 | 0.534871 | 0.00405 |
| RP11-592N21.1 | -1.52169 | -0.39847 | 0.007521 |
| RP4-765C7.2 | -1.75039 | 2.544176 | 2.40E-08 |
| RP11-613F7.1 | -1.11917 | 1.777513 | 5.33E-05 |
| RP11-641D5.1 | -1.17055 | 8.956216 | 2.47E-24 |
| AC012066.1 | -1.13795 | 0.672341 | 0.000693 |
| CTD-3035D6.1 | -2.10686 | 2.027577 | 1.43E-08 |
| RPS7P11 | -1.28341 | 1.854422 | 1.52E-08 |
| AKR1B1P2 | -1.00424 | 1.426824 | 0.004815 |
| RP11-112J1.1 | -1.3225 | 9.354491 | 1.11E-21 |
| RP11-380G5.3 | -1.47222 | 3.281834 | 6.21E-14 |
| KLRK1 | 1.662851 | 1.575983 | 1.78E-05 |
| CTD-2270N23.1 | -1.00809 | 4.328678 | 5.60E-12 |
| EEF1B2P2 | -1.16486 | 3.855528 | 4.42E-09 |
| RP11-829H16.2 | -1.7903 | 0.95853 | 1.86E-06 |
| NPM1P6 | -1.04157 | 3.260545 | 2.62E-07 |
| AC092610.12 | -1.41898 | 3.078988 | 1.41E-10 |
| RP11-314A20.1 | -1.65682 | 1.381722 | 8.09E-06 |
| RP1-102E24.1 | -1.42257 | 0.84316 | 3.26E-05 |
| AC012487.2 | 1.200505 | 0.923825 | 0.000876 |
| AC010336.1 | -1.7465 | 0.183365 | 0.000972 |
| RP11-108K14.4 | 1.262879 | 4.788261 | 4.00E-06 |
| VAC14-AS1 | -1.12855 | 0.723576 | 0.00686 |
| RPS3AP26 | -1.00825 | 8.054528 | 3.49E-20 |
| CTB-13H5.1 | -1.10298 | 4.494781 | 1.30E-11 |
| RPS15AP1 | -1.29627 | 9.44918 | 1.49E-19 |
| RPS19P1 | -1.18717 | 6.949939 | 1.55E-26 |
| AC010468.1 | -1.25074 | 3.548739 | 1.70E-08 |
| SHFM1P1 | -1.21255 | 3.682825 | 1.44E-11 |
| RP11-229M1.2 | -1.02641 | 1.819574 | 5.74E-05 |
| PRDX2P1 | -1.06076 | 0.053845 | 0.003099 |
| C9orf38 | -2.73764 | -0.43673 | 4.88E-05 |
| RP11-1023L17.2 | -1.50615 | 0.798507 | 0.002456 |
| PI4KAP1 | 1.907949 | 0.905751 | 0.000338 |
| RP3-375P9.2 | -1.16194 | 4.156089 | 1.90E-10 |
| RP1-182O16.1 | -1.43451 | -0.32016 | 0.006224 |
| RSL24D1P1 | -1.09399 | 1.174326 | 0.003541 |
| RP11-367G18.2 | -1.80801 | 0.732401 | 1.37E-05 |
| RP11-475C16.1 | -1.0227 | 11.21278 | 1.97E-15 |
| RP3-340B19.2 | -1.5408 | 4.702166 | 5.68E-21 |
| RPL23P8 | -1.20787 | 7.9783 | 1.76E-24 |
| RP1-292B18.1 | -1.13365 | 0.607872 | 0.001567 |
| RP11-40C6.2 | -1.50095 | 7.49964 | 2.51E-28 |
| HIST2H2BD | 1.443694 | -0.40449 | 0.006613 |
| RP11-572P18.1 | -1.27012 | 5.068625 | 3.71E-19 |
| OSTCP4 | -1.01436 | 1.11377 | 0.003666 |
| CEBPD | 1.392702 | 4.229177 | 0.000519 |
| MRPS21P3 | -1.65417 | 0.582082 | 0.001924 |
| TMEM14E | -1.90514 | 0.788461 | 1.86E-05 |
| RP11-545E17.3 | -1.07594 | 2.450229 | 1.66E-05 |
| AC093690.1 | -1.40933 | 1.464966 | 2.44E-06 |
| TMSB4XP4 | -1.05342 | 1.98178 | 0.000813 |
| RP3-476K8.4 | -1.80253 | 0.010238 | 0.001596 |
| RPS20P14 | -1.23352 | 8.545044 | 2.99E-24 |
| RP11-533O20.2 | -1.22804 | 1.18651 | 7.52E-06 |
| RP11-274B18.3 | 2.278466 | -0.36845 | 0.000377 |
| RPS24P8 | -1.10247 | 3.413837 | 3.55E-06 |
| RP11-343H5.4 | -1.34957 | 5.839303 | 1.04E-10 |
| HAUS1P2 | -1.85186 | 0.175092 | 3.13E-06 |
| RPL19P12 | -1.02148 | 3.870839 | 1.77E-06 |
| AC017035.2 | -2.15224 | 0.030187 | 3.21E-05 |
| RP11-270C12.3 | -1.17257 | 6.693939 | 5.69E-18 |
| RP11-51O6.1 | -1.12735 | 10.39695 | 3.97E-21 |
| RP11-54O7.11 | -2.04185 | -0.53898 | 0.004856 |
| AB019441.29 | -1.32402 | 6.457055 | 4.97E-32 |
| HSPA7 | 1.746485 | 2.505844 | 0.005785 |
| AP001055.6 | 1.079583 | 1.855128 | 3.82E-05 |
| RP11-262D11.2 | -1.21561 | 8.208002 | 5.41E-24 |
| RPL35P5 | -1.166 | 7.598148 | 3.39E-26 |
| RP4-604A21.1 | -1.26936 | 4.905776 | 5.96E-17 |
| AC092933.3 | -1.21336 | 3.842719 | 6.26E-13 |
| RP13-258O15.1 | -1.19156 | 2.985317 | 3.81E-08 |
| RP3-340N1.5 | -1.72605 | 0.716994 | 0.000117 |
| RP11-731I19.1 | -1.56259 | -0.19891 | 0.010079 |
| RP4-706A16.3 | -1.41089 | 4.671915 | 3.12E-21 |
| AC022431.1 | -1.26413 | 9.663209 | 7.77E-26 |
| RPL37AP1 | -1.31966 | 9.248962 | 1.91E-33 |
| MIR29B1 | -1.03407 | 2.662724 | 0.000191 |
| RPS7P10 | -1.07054 | 5.929246 | 3.66E-15 |
| RP11-166N17.3 | -1.55618 | 0.252742 | 0.001417 |
| RP3-417G15.1 | -1.40167 | 7.455455 | 1.07E-32 |
| RP11-543P15.1 | -1.36967 | 10.14997 | 3.02E-26 |
| MYL6P3 | -1.96153 | -0.13388 | 2.61E-05 |
| SUB1P1 | -1.05967 | 5.062168 | 1.33E-07 |
| RP11-791G16.2 | -1.00689 | 4.677372 | 7.15E-13 |
| RP11-864N7.2 | -1.19984 | 11.53123 | 1.01E-22 |
| PIN4P1 | -1.06784 | 3.613503 | 1.98E-09 |
| RP4-575N6.2 | -1.09407 | 0.388514 | 0.009586 |
| OST4 | -1.03642 | 6.674712 | 2.03E-18 |
| AC096558.1 | -1.59878 | 0.641323 | 1.46E-05 |
| RP13-39P12.3 | 1.213513 | 0.964671 | 0.003465 |
| RP11-57H12.5 | 1.59281 | -0.07001 | 0.003384 |
| RP11-159C21.4 | -1.08992 | 10.31923 | 1.24E-20 |
| SNRPD2P1 | -1.13007 | 6.212285 | 2.29E-17 |
| RP11-378J18.6 | -1.02197 | 4.374974 | 5.57E-11 |
| RLIMP1 | -1.28182 | 1.105132 | 0.001374 |
| RPL35AP21 | -1.39189 | 2.257785 | 1.00E-07 |
| ARSD-AS1 | 1.122371 | 0.847382 | 0.003743 |
| RP5-1049G16.4 | -2.00627 | -0.00921 | 0.001907 |
| AC092431.2 | -1.75041 | 0.058069 | 0.000526 |
| TMSB4XP6 | -1.24929 | 2.525803 | 1.25E-07 |
| RP11-244J10.1 | -1.88274 | 3.779662 | 7.06E-17 |
| RP3-323N1.2 | -2.50252 | 0.621116 | 1.68E-07 |
| RP4-635E18.6 | 1.365801 | 0.240463 | 0.000797 |
| U73166.2 | -1.3429 | 1.369917 | 1.64E-05 |
| AC021016.7 | -1.54932 | 2.203451 | 0.000185 |
| RPS23P8 | -1.2702 | 10.24143 | 3.10E-24 |
| RP11-409K20.6 | -1.96674 | -0.35808 | 0.00082 |
| RP11-318C24.1 | -1.87705 | 4.207147 | 1.51E-10 |
| RPL31P2 | -2.13911 | -0.00772 | 0.001701 |
| RPS18P12 | -1.43174 | 4.757726 | 9.98E-18 |
| BTF3P10 | -1.02823 | 0.720784 | 0.002081 |
| AC125232.1 | 1.022557 | 0.843718 | 0.003 |
| RP3-426I6.2 | -1.3544 | 1.395963 | 9.75E-05 |
| RP11-422P24.9 | -1.33165 | 7.171862 | 1.03E-21 |
| HINT1P1 | -1.17293 | 4.798172 | 9.33E-19 |
| USMG5P1 | -1.52347 | 5.388398 | 2.60E-32 |
| AC013264.2 | -1.36798 | 3.877314 | 9.96E-08 |
| RFESDP1 | -1.20682 | 0.94198 | 0.00128 |
| AC079922.2 | -1.29162 | 0.841977 | 0.000592 |
| MIR4426 | -1.42279 | 5.271381 | 6.71E-19 |
| RP11-558F24.4 | -1.45608 | 1.146155 | 1.17E-05 |
| RP13-93L13.2 | -1.76235 | 1.243699 | 1.02E-05 |
| SNRPFP1 | -1.36913 | 3.946711 | 7.99E-21 |
| NDUFB1P1 | -1.09792 | 4.979366 | 2.63E-12 |
| RP11-146N23.1 | -1.03707 | 3.769059 | 8.03E-11 |
| ATP5LP2 | -1.16612 | 5.161236 | 1.67E-17 |
| C9orf172 | 1.092661 | 1.62734 | 0.001237 |
| RP4-591N18.2 | -1.9907 | 0.081392 | 0.000109 |
| EEF1A1P3 | -2.64727 | 0.344699 | 8.61E-08 |
| RPL34P27 | -1.29338 | 2.30957 | 4.36E-06 |
| TRBV29-1 | -1.07065 | 4.791993 | 1.07E-09 |
| RPS11P5 | -1.15576 | 10.75999 | 1.84E-26 |
| RP11-558F24.2 | -2.99602 | -0.04797 | 4.51E-07 |
| SNRPEP4 | -1.1298 | 4.253939 | 4.36E-11 |
| BTBD18 | -1.95817 | 0.648625 | 2.11E-06 |
| RP1-187B23.1 | -1.36944 | 0.796594 | 0.005516 |
| AC016831.7 | -2.32996 | 0.470638 | 2.08E-06 |
| RP11-571F15.3 | -1.2441 | 0.269653 | 0.008354 |
| RP11-389O22.4 | -2.15267 | 0.567501 | 2.25E-06 |
| RP11-169K16.7 | -1.07068 | 6.704484 | 1.76E-15 |
| RPS20P10 | -1.52484 | 1.089739 | 0.0015 |
| RP11-123J14.2 | -1.50496 | -0.02116 | 0.001166 |
| RP11-254B13.4 | -1.99239 | -0.37645 | 0.00131 |
| RP11-761N21.2 | -1.43524 | 6.794813 | 6.68E-28 |
| AC007278.3 | 2.205557 | 2.457727 | 1.25E-08 |
| AC096664.1 | -1.94881 | 0.115975 | 9.55E-06 |
| RP11-442A13.1 | -1.04245 | 10.1666 | 5.74E-22 |
| RPS3AP6 | -1.14369 | 9.810561 | 3.67E-21 |
| AC013474.4 | -1.09853 | 0.638994 | 0.001732 |
| RPL39P3 | -1.27424 | 10.67112 | 2.64E-23 |
| AC010240.2 | -3.02756 | -0.69101 | 0.000129 |
| RP11-626A1.1 | -1.45398 | 1.112171 | 8.79E-05 |
| CTA-276O3.4 | -1.49595 | 4.257148 | 4.85E-16 |
| SNRPGP10 | -1.5009 | 1.806805 | 5.74E-07 |
| RP11-480I12.2 | -1.155 | 4.994795 | 1.73E-13 |
| RP1-159A19.3 | -1.50105 | 2.221724 | 8.06E-10 |
| RP13-444K19.1 | -1.33535 | 3.130856 | 6.99E-12 |
| COX7CP1 | -1.42597 | 3.451736 | 1.19E-13 |
| RP1-3J17.3 | -2.03932 | -0.185 | 0.000122 |
| GUSBP5 | -1.05056 | 1.609935 | 0.002626 |
| RPL34P34 | -1.19241 | 2.238593 | 2.12E-05 |
| AC007278.2 | 2.143652 | 1.125017 | 0.000183 |
| RPL31P63 | -1.71699 | 1.440327 | 1.99E-09 |
| COX7A2P2 | -1.14462 | 5.629057 | 3.29E-14 |
| LINC00299 | 1.155194 | 0.794701 | 0.007306 |
| RPL24P8 | -1.09345 | 8.146416 | 3.39E-23 |
| RP11-535C21.3 | -1.28554 | 0.290917 | 0.003832 |
| ANKRD44-IT1 | -1.24895 | 1.803653 | 1.80E-05 |
| AP000361.2 | -1.52515 | 1.985446 | 7.08E-09 |
| RPS15AP38 | -1.62706 | 1.387591 | 2.99E-07 |
| RPL30P4 | -1.19466 | 8.007476 | 1.00E-21 |
| TRBV3-1 | -1.14791 | 4.101877 | 3.28E-13 |
| UQCRBP1 | -1.25068 | 5.29904 | 5.33E-17 |
| hsa-mir-6723 | -3.19451 | 0.684967 | 4.44E-05 |
| AC116366.5 | -1.463 | 0.678653 | 0.000158 |
| SCARNA7 | -3.0216 | 0.497684 | 6.53E-12 |
| SCARNA10 | -2.65912 | 2.301651 | 2.25E-12 |
| SNORD13 | -1.94032 | 0.195582 | 0.000847 |
| RPL34P31 | -1.51386 | 2.697475 | 1.40E-11 |
| RP11-464D20.2 | -1.2855 | 3.492523 | 7.78E-11 |
| RP11-771F20.1 | -1.36161 | 0.317839 | 0.000117 |
| RPL37P2 | -1.34148 | 5.217582 | 8.54E-25 |
| NME1 | -1.02534 | 3.248456 | 1.53E-05 |
| RP4-800G7.1 | -1.48149 | 1.25143 | 6.71E-05 |
| IGKV1-6 | -1.79688 | 2.077181 | 0.003427 |
| RP11-587D21.1 | -1.77819 | 0.24412 | 9.33E-05 |
| RP11-36C20.1 | -1.05533 | 10.53814 | 1.27E-21 |
| RPL34P18 | -1.30456 | 9.076111 | 2.18E-20 |
| RP11-3P17.4 | -1.14135 | 3.013971 | 2.05E-09 |
| RP11-102M11.1 | -1.11038 | 1.787828 | 4.84E-06 |
| RP11-488C13.1 | -1.12628 | 8.192265 | 3.61E-28 |
| RP1-89D4.1 | -1.75063 | 1.005462 | 6.05E-06 |
| IGKV3-11 | -1.50643 | 4.179581 | 0.007686 |
| PDXP | 1.038566 | 0.827617 | 0.003952 |
| RP11-796G6.1 | -1.07621 | 4.343512 | 2.02E-11 |
| RPS15AP24 | -1.03801 | 2.010296 | 6.89E-05 |
| RP11-80H8.4 | 1.065474 | 0.72109 | 0.006589 |
| RPL31P49 | -1.30129 | 1.604213 | 9.57E-06 |
| ZNF90P1 | -1.8407 | -0.16388 | 0.001551 |
| RP11-234A1.1 | -1.10928 | 10.74227 | 7.68E-19 |
| RPS21P4 | -1.25815 | 9.362461 | 2.88E-26 |
| CTD-2161E19.1 | -1.77537 | -0.27348 | 6.19E-05 |
| AC007620.3 | 1.352611 | 2.224678 | 6.96E-05 |
| RP11-436H11.1 | -1.09689 | 5.064511 | 1.79E-17 |
| RP11-507E23.1 | -1.30505 | 1.382344 | 0.000154 |
| MRPL33 | -1.04796 | 4.260318 | 6.00E-11 |
| UBA52P5 | -1.02691 | 5.260457 | 3.08E-15 |
| RPS23P1 | -1.23797 | 6.526808 | 7.39E-22 |
| RP11-293G6 | -1.36772 | -0.12428 | 0.007308 |
| ANTXRLP1 | -1.02104 | 2.321817 | 0.001568 |
| RPL37P23 | -1.37349 | 6.82076 | 3.21E-24 |
| RP11-390K5.1 | -2.62772 | 0.111036 | 1.18E-08 |
| RPL5P17 | -1.4874 | 1.282982 | 2.20E-05 |
| RP11-16F15.1 | -1.78434 | 0.543773 | 3.10E-05 |
| RPS27P27 | -1.19174 | 3.584916 | 5.50E-13 |
| RPS12P20 | -2.0128 | -0.16501 | 0.001272 |
| RP11-114H7.1 | -1.15463 | 8.992248 | 5.52E-29 |
| C7orf13 | -1.28986 | 1.477138 | 0.002434 |
| RP11-425L10.1 | -1.06133 | 10.10132 | 6.69E-20 |
| CTB-47B8.1 | -1.02794 | 1.299673 | 0.001883 |
| RP11-466H18.1 | -1.35695 | 9.852326 | 1.94E-25 |
| LILRA6 | 1.834513 | 1.979892 | 0.006397 |
| RP1-181J22.1 | -2.14737 | 0.847698 | 4.67E-05 |
| RP11-277P12.20 | 1.424739 | 2.161966 | 1.02E-05 |
| KB-1208A12.3 | -1.1289 | 7.996868 | 7.02E-26 |
| EXTL3-AS1 | 1.813237 | 1.491804 | 7.22E-05 |
| CTD-2024I7.13 | -2.03156 | -0.0645 | 4.89E-05 |
| LINC00926 | 1.580536 | 4.421629 | 2.34E-05 |
| POU5F2 | -1.96181 | -0.09546 | 0.000465 |
| RP11-227H4.5 | 1.119844 | 1.412633 | 0.009775 |
| CTNNAP1 | 1.089724 | 1.738309 | 0.001316 |
| CTD-2206G10.2 | -1.18289 | 8.046474 | 1.03E-17 |
| TRGV7 | 1.281523 | 0.179139 | 0.004274 |
| RP11-616K22.1 | -2.17856 | -0.07789 | 3.00E-06 |
| RP11-478C6.4 | -1.55948 | 6.110369 | 1.80E-46 |
| RP11-47F1.1 | -1.07169 | 0.424917 | 0.00321 |
| RP11-54F2.1 | 1.679113 | -0.39673 | 0.007298 |
| GOLGA5P1 | -1.04242 | 1.436424 | 0.004158 |
| RP11-503N18.5 | -1.43073 | 0.843667 | 0.000265 |
| CTB-109A12.1 | -2.16474 | 1.657985 | 2.59E-09 |
| FKBP4P1 | -1.47044 | 0.08869 | 0.004444 |
| MALAT1 | -2.05943 | 12.52746 | 1.06E-46 |
| RP11-539L10.3 | -1.38913 | 1.573825 | 0.000202 |
| SCARNA6 | -1.26879 | 1.575102 | 4.09E-05 |
| SCARNA17 | -2.36482 | 2.497862 | 5.22E-21 |
| SCARNA5 | -2.70587 | 2.220973 | 5.48E-13 |
| SCARNA18 | -2.57746 | 1.368598 | 2.20E-11 |
| SCARNA21 | -1.4364 | 1.993143 | 2.17E-06 |
| RP11-44N11.1 | -1.42986 | -0.10375 | 0.004986 |
| RP11-930P14.1 | 1.561809 | 0.186326 | 0.000493 |
| RP11-39H3.1 | -1.31944 | 0.823802 | 0.002856 |
| CTC-248O19.1 | 1.108936 | 2.271392 | 0.00017 |
| CTB-79E8.3 | -1.08813 | 6.47913 | 8.49E-21 |
| RP11-473O4.3 | -1.79297 | -0.15783 | 0.000452 |
| IGHGP | -2.78614 | 4.977834 | 0.006222 |
| RP11-10N23.2 | 1.020557 | 1.509282 | 0.001968 |
| LYN | 1.027787 | 4.562726 | 0.007184 |
| RP11-34P1.2 | -1.25192 | 3.941236 | 7.01E-12 |
| RP11-261P9.4 | 1.113493 | 1.763651 | 0.00065 |
| SCARNA9 | -1.76332 | 3.738164 | 2.86E-17 |
| RP11-685M7.5 | -1.10284 | 1.556544 | 0.000544 |
| CTD-2616J11.2 | 1.670444 | 1.099522 | 0.002751 |
| KLRC4-KLRK1 | 1.655661 | 0.510745 | 0.002554 |
| RP11-841C19.3 | -1.11268 | 2.775354 | 4.64E-09 |
| TAS2R30 | -2.47967 | -0.29782 | 0.000931 |
| RP11-664D1.1 | -1.26223 | 4.287717 | 2.73E-14 |
| SNRPEP2 | -1.16382 | 2.940933 | 3.89E-10 |
| TAS2R18 | -1.74698 | -0.01103 | 0.008457 |
| RP11-609L23.2 | -1.01168 | 1.172715 | 0.001237 |
| RP11-570L15.2 | -2.67942 | -0.53943 | 0.000748 |
| RN7SL1 | -1.83283 | 5.099576 | 3.75E-21 |
| RP11-638I2.10 | -1.3135 | 3.760931 | 5.31E-11 |
| DNAJC19P9 | -1.01644 | 4.649188 | 1.25E-14 |
| RP11-644F5.15 | -1.11251 | 1.066253 | 0.000728 |
| RPPH1 | -1.95362 | 3.560216 | 3.94E-13 |
| RP11-488C13.6 | 1.354344 | 2.013853 | 5.57E-09 |
| CTD-2017C7.2 | -1.16677 | 0.401852 | 0.001665 |
| CAP2P1 | -3.57351 | -0.47645 | 2.23E-07 |
| RP11-244F12.3 | 1.068028 | 0.370622 | 0.006439 |
| RP11-707P17.1 | 1.272374 | 1.354428 | 0.006939 |
| RP11-1100L3.8 | 2.581589 | 2.859425 | 0.004498 |
| RP11-44F14.5 | -1.68978 | -0.19978 | 8.04E-05 |
| RP11-304L19.1 | 1.009273 | 2.293199 | 3.98E-06 |
| RP11-66N11.8 | -1.16424 | 1.171282 | 1.85E-06 |
| COX6CP1 | -1.17804 | 1.832086 | 2.87E-05 |
| RP11-106M3.2 | 1.085839 | 0.644136 | 0.007456 |
| RP11-432I5.1 | -1.63097 | -0.20843 | 0.003576 |
| RP1-90J20.11 | 1.021845 | 1.716265 | 0.001058 |
| RP11-474B12.1 | -2.66721 | -0.40149 | 0.000845 |
| RP11-553K8.5 | -1.44977 | 0.918436 | 0.001823 |
| SUB1P3 | -1.13381 | 2.924223 | 2.48E-06 |
| CTD-2561B21.5 | -1.42842 | 1.405373 | 2.92E-07 |
| RP11-96D1.9 | -1.00628 | 0.804612 | 0.006938 |
| AC123768.4 | 1.287371 | 0.38026 | 0.005039 |
| CTD-2561B21.11 | -1.41284 | 1.144696 | 9.22E-05 |
| KARSP3 | -1.74992 | -0.3007 | 0.002374 |
| TAPT1-AS1 | -1.24117 | 9.334876 | 2.18E-26 |
| MIR4665 | 1.055702 | 1.210856 | 0.001206 |
| RP11-498C9.15 | -1.03508 | 2.111295 | 2.19E-05 |
| RP11-321A17.3 | -1.26727 | 4.709911 | 1.86E-16 |
| SNRPGP2 | -1.19264 | 2.792512 | 3.78E-07 |
| RP4-777O23.2 | 1.379235 | 0.250525 | 0.000586 |
| RP11-4F22.2 | -2.04764 | -0.24826 | 0.00047 |
| hsa-mir-4537 | 1.502423 | 0.479697 | 0.004964 |
| MIR4523 | 1.290386 | 1.17934 | 4.68E-05 |
| RP11-16C1.2 | -2.12254 | 0.08571 | 2.75E-05 |
| RP11-6N17.4 | -1.37937 | 1.149225 | 0.000101 |
| RN7SL2 | -2.0711 | 5.230216 | 8.16E-20 |
| MIR151B | -2.20936 | 0.458225 | 5.82E-08 |
| AL122127.2 | 2.785223 | -0.1076 | 1.29E-07 |
| RP11-927P21.4 | 1.006764 | 1.983533 | 5.94E-05 |
| MIR3609 | -3.41485 | 1.011679 | 1.99E-13 |
| UBL5P2 | -1.30114 | 5.831427 | 3.79E-31 |
| AL122127.4 | 1.761317 | 0.242686 | 0.00391 |
| CTC-268N12.2 | -1.48428 | 0.108657 | 0.001275 |
| CTC-507E2.1 | -1.69176 | 0.169134 | 0.002945 |
| RP11-75C10.9 | -1.02963 | 2.092264 | 0.000488 |
| RP11-126O1.5 | 1.496093 | 2.446693 | 2.59E-08 |
| AC007229.3 | -1.05359 | 2.671166 | 3.71E-07 |
| RP11-28F1.2 | -1.11383 | 1.000148 | 0.007078 |
| RP11-358B23.5 | -1.07306 | 1.662328 | 0.003989 |
| RP11-171I2.1 | -1.75516 | 0.109558 | 0.008005 |
| RP11-91G21.1 | -1.70612 | 0.499501 | 3.54E-06 |
| AL022328.1 | 3.030614 | 0.409678 | 3.34E-06 |
| C20ORF135 | 1.107746 | 1.365308 | 0.001567 |
| CTD-3187F8.14 | 2.820689 | 0.271786 | 0.000117 |
| RP11-145M9.4 | 1.018639 | 2.639175 | 0.000737 |
| FLJ20306 | 1.066018 | 1.319574 | 0.005091 |
| KCNQ1OT1 | -1.29892 | 3.983239 | 2.53E-13 |
| RP11-3P17.5 | -1.00034 | 7.267655 | 1.54E-16 |
| RMRP | -2.09578 | 4.539358 | 4.30E-24 |
| SCARNA2 | -1.28352 | 2.032527 | 5.45E-05 |
| GAS5-AS1 | -1.06715 | 4.117509 | 4.18E-12 |
| RNU11 | -1.97005 | 1.166189 | 0.000136 |
| RP5-1139B12.4 | -1.4374 | 1.107226 | 0.00353 |
| RP11-15E18.5 | -1.18399 | 2.653673 | 8.20E-07 |
| RP11-138A9.1 | -2.34747 | 0.567043 | 9.84E-09 |
| RP11-532F12.6 | -3.301 | -0.86148 | 0.000288 |
| CTD-2006H14.2 | 1.058683 | 1.275486 | 0.001876 |
| RP11-215G15.5 | 1.176214 | 1.338423 | 0.001139 |
| AC005618.6 | -1.31006 | 0.340487 | 0.005713 |
| RP11-755B10.4 | -1.35989 | 1.107845 | 0.000779 |
| U47924.31 | -1.01148 | 6.618726 | 4.22E-19 |
| CTD-2154B17.4 | 1.422405 | 0.337607 | 0.003632 |
| RP11-563N4.1 | -1.15878 | 0.166701 | 0.007372 |
| RP4-673M15.1 | 1.273109 | 2.061084 | 4.87E-05 |
| RP11-20I20.4 | 1.937674 | 0.425843 | 5.07E-05 |
| RP11-138A9.2 | -2.47561 | 2.24447 | 1.02E-15 |
| RP11-386I14.4 | -1.31373 | 3.258115 | 0.002086 |
