# Supplementary material for: CD4+ T cells from children with active juvenile idiopathic arthritis show altered chromatin features associated with transcriptional abnormalities
Source: Sci Rep. 2021 Feb 17;11:4011. doi: 10.1038/s41598-021-82989-5 (PMC7889855; doi:10.1038/s41598-021-82989-5)
Supplement: Supplementary file 4 — Supplementary Table 2. [file 41598_2021_82989_MOESM4_ESM.docx]

**Table S2**

Differentially expressed genes between ADT and CRM, whose log10(Fold-change) was at least 1 and whose FDR was less than or equal to 0.05.

| **Gene Name** | **Log(fold-change)** | **Log(CPM)** | **P-Value** |
| --- | --- | --- | --- |
| TMEM132A | -1.51302 | 0.181642 | 0.004378 |
| PLEKHG6 | -2.08259 | 0.983904 | 1.11E-06 |
| RPS20 | -1.20668 | 8.882329 | 2.97E-32 |
| FAM65C | -1.73794 | 2.424137 | 1.05E-10 |
| CC2D2A | -1.11947 | 1.256385 | 0.00602 |
| PRSS8 | -2.30189 | 0.163833 | 6.45E-05 |
| SEZ6 | -1.77157 | 0.291142 | 0.002234 |
| RPL31 | -1.06953 | 7.098693 | 1.06E-12 |
| MGST2 | -1.32198 | 2.040659 | 0.003994 |
| CHGB | -2.55258 | 0.62162 | 0.002056 |
| OTUB2 | -1.45472 | 1.932201 | 2.55E-05 |
| ANKRD24 | 1.344355 | 1.457685 | 0.000598 |
| RGS1 | -1.47188 | 4.390751 | 0.000697 |
| ANGPT2 | -1.84228 | 0.740372 | 0.000447 |
| CDHR5 | -1.79608 | 1.404347 | 6.76E-05 |
| SUSD2 | -1.16854 | 1.710587 | 0.002651 |
| LGALS1 | -1.00051 | 4.55737 | 2.83E-05 |
| ERH | -1.04301 | 5.102625 | 3.94E-16 |
| SLC10A1 | -1.88554 | 0.793786 | 0.000561 |
| TCL1A | -1.76227 | 2.570026 | 0.001377 |
| ESRP2 | -1.33562 | 2.04909 | 2.91E-05 |
| FAM189A1 | -2.34391 | -0.13849 | 0.000905 |
| PDGFRL | -1.07347 | 1.247564 | 0.002486 |
| PEX11G | -1.65826 | 2.142394 | 2.59E-07 |
| CLC | -2.38526 | 3.911297 | 0.002693 |
| ICAM4 | -1.53794 | 0.644031 | 0.001672 |
| TSPAN13 | -1.66534 | 1.540786 | 0.001326 |
| SEC61B | -1.02843 | 4.869127 | 7.25E-08 |
| RPL19 | -1.0214 | 8.739943 | 3.36E-18 |
| HSD17B1P1 | 1.198786 | 2.13667 | 0.000112 |
| RPL34 | -1.21961 | 6.592941 | 1.54E-12 |
| TENC1 | -1.67169 | 2.426935 | 2.72E-08 |
| GLI1 | -1.37862 | 2.300584 | 3.19E-06 |
| C12orf57 | -1.00335 | 7.111171 | 3.08E-17 |
| CLEC4A | -1.6627 | 2.013254 | 7.96E-05 |
| TMEM14C | -1.06788 | 4.827842 | 1.00E-11 |
| RPS12 | -1.1734 | 6.723759 | 3.27E-12 |
| COX7A2 | -1.28421 | 5.011059 | 5.63E-20 |
| SELK | -1.01382 | 4.402176 | 1.64E-07 |
| SF3B14 | -1.0313 | 4.614762 | 3.60E-11 |
| CHD5 | -2.03664 | 0.131106 | 0.00092 |
| MUC5B | -2.14915 | 0.041318 | 0.000439 |
| NDUFB3 | -1.19397 | 4.173273 | 1.34E-11 |
| CYSTM1 | -1.06467 | 2.099219 | 0.000163 |
| CHRNA2 | -2.37185 | 0.287264 | 4.49E-06 |
| NACA3P | -1.05033 | 5.742379 | 7.13E-12 |
| ABCC11 | 1.05775 | 2.261302 | 7.02E-07 |
| NR4A1 | -2.57564 | 2.929744 | 1.42E-05 |
| KCNS1 | -1.81552 | 0.967105 | 0.003768 |
| RP11-663P9.2 | -1.04739 | 5.361019 | 3.90E-11 |
| HIST1H1D | -1.92115 | 2.257685 | 3.63E-06 |
| MT2A | -1.16404 | 2.993589 | 0.000209 |
| RPL23 | -1.32232 | 6.898069 | 4.04E-20 |
| FOSB | -1.10235 | 3.793442 | 0.00226 |
| SNRPD2 | -1.21506 | 6.834396 | 9.54E-24 |
| COX6B1 | -1.30509 | 4.725003 | 2.01E-14 |
| COX7C | -1.26913 | 7.145965 | 3.77E-29 |
| SPINK2 | -1.31809 | 1.069601 | 0.001189 |
| NEDD8 | -1.09158 | 5.155254 | 6.79E-20 |
| LPPR3 | -2.15503 | 0.754211 | 3.59E-08 |
| RPL36 | -1.06463 | 6.984157 | 1.59E-18 |
| RPL27 | -1.24369 | 8.36268 | 1.92E-27 |
| MAP1B | -2.13786 | 0.04471 | 0.002002 |
| ITGB4 | -1.60933 | 0.651055 | 0.000523 |
| FST | -3.11773 | 2.226043 | 5.17E-05 |
| TIMM10 | -1.09938 | 3.550012 | 5.46E-08 |
| KLB | -2.1463 | 0.488868 | 0.004974 |
| SDS | -1.86853 | -0.03228 | 0.004817 |
| PKIB | -1.52752 | 0.584306 | 0.002171 |
| NACAD | -2.64785 | 0.785682 | 2.36E-07 |
| TXN | -1.10173 | 3.620067 | 1.66E-07 |
| WDR38 | -1.44824 | 1.148442 | 0.003148 |
| RPL35 | -1.21052 | 7.548304 | 2.24E-23 |
| TMEM14B | -1.01904 | 5.050541 | 1.92E-12 |
| C11orf1 | -1.04949 | 3.6247 | 5.77E-08 |
| THBS1 | 1.627529 | 1.537632 | 0.00623 |
| RPS24 | -1.22972 | 8.221546 | 6.30E-30 |
| CELA1 | -1.04787 | 1.92503 | 0.005047 |
| GREB1L | -1.77196 | 0.773083 | 0.000729 |
| PMAIP1 | -1.26171 | 3.525668 | 0.000266 |
| ACPT | -1.23861 | 0.599649 | 0.005279 |
| RPS11 | -1.05246 | 8.557249 | 8.32E-18 |
| RPL11 | -1.06562 | 9.294357 | 7.14E-18 |
| RPS8 | -1.00426 | 8.86687 | 9.14E-20 |
| RPS27A | -1.22114 | 8.020824 | 1.72E-22 |
| RPL32 | -1.16305 | 9.031874 | 2.87E-22 |
| EAF2 | -1.22117 | 3.258587 | 0.004457 |
| AC004453.8 | -1.1065 | 10.41451 | 1.17E-22 |
| FAU | -1.06105 | 7.39762 | 2.56E-14 |
| TIMM8B | -1.19751 | 4.271714 | 9.72E-10 |
| IL18 | -1.36335 | 1.260513 | 0.004095 |
| FARP1 | -1.06179 | 2.375197 | 0.00268 |
| LY96 | -1.27222 | 1.941549 | 0.000231 |
| DBI | -1.10853 | 5.077327 | 4.39E-14 |
| C14orf2 | -1.02214 | 5.422966 | 2.19E-17 |
| RPL30 | -1.04022 | 9.217037 | 1.23E-21 |
| CD1C | -1.71477 | 3.118001 | 0.000814 |
| AC007383.6 | -1.04122 | 4.08841 | 1.65E-09 |
| PTGIR | 1.279235 | 3.492004 | 0.000241 |
| AIRE | 1.009731 | 2.115982 | 0.005135 |
| TLCD1 | -2.16688 | 0.885411 | 3.32E-06 |
| CHRNB2 | -1.90271 | 1.653329 | 6.55E-06 |
| RPL26 | -1.20619 | 7.388305 | 2.13E-18 |
| WNT4 | -2.17725 | 0.987833 | 5.69E-09 |
| KCNJ9 | -2.42576 | 0.115841 | 9.89E-06 |
| BOLA3 | -1.01924 | 2.224108 | 0.000196 |
| RPL22L1 | -1.06053 | 4.223501 | 3.65E-11 |
| DNASE1L3 | -1.82417 | 0.981463 | 0.004738 |
| RPL39L | -1.06138 | 1.822682 | 0.00061 |
| NDUFS4 | -1.36542 | 3.326319 | 2.72E-10 |
| RPS14 | -1.16107 | 8.668409 | 5.44E-24 |
| COX6C | -1.1814 | 5.99898 | 5.60E-23 |
| RPL36AL | -1.11024 | 8.229691 | 1.10E-17 |
| RPL27A | -1.00459 | 8.058904 | 1.34E-13 |
| TTYH1 | -1.4472 | 1.110733 | 0.001489 |
| LAIR2 | -2.42762 | 1.765094 | 3.93E-05 |
| HIST1H1E | -2.07473 | 1.286787 | 6.58E-06 |
| DEGS2 | -2.70535 | 1.786239 | 3.00E-10 |
| NDUFS5 | -1.21725 | 5.454812 | 3.34E-19 |
| ENHO | -1.82097 | 1.66992 | 0.001314 |
| RP11-220D10.1 | -1.13365 | 7.240343 | 1.16E-23 |
| RNASE6 | -1.36397 | 4.143889 | 0.000221 |
| CD52 | -1.07183 | 9.769504 | 1.40E-19 |
| CDH2 | -3.00347 | -0.25827 | 0.000629 |
| LILRA3 | -4.47105 | 0.933875 | 0.000312 |
| LRRC8E | -1.60468 | -0.00284 | 0.001445 |
| COL8A2 | -1.80493 | 0.835072 | 0.002861 |
| RPS21 | -1.02266 | 3.799746 | 1.41E-09 |
| MRPL13 | -1.01881 | 2.898755 | 4.42E-06 |
| ALG14 | -1.01736 | 2.456112 | 0.000253 |
| MYEOV2 | -1.01046 | 4.183743 | 9.75E-10 |
| ZBTB8OSP2 | -1.45805 | 3.776326 | 1.77E-09 |
| RPL38 | -1.0543 | 5.655587 | 2.29E-14 |
| KRT2 | -1.04226 | 1.463778 | 0.001495 |
| UQCRH | -1.07557 | 5.466427 | 3.52E-16 |
| C9orf131 | -1.76611 | 0.99472 | 4.75E-05 |
| ADCY6 | 1.95011 | -0.45914 | 0.00498 |
| CNTNAP2 | -2.55763 | 0.403479 | 0.004147 |
| LINC00116 | -1.01118 | 3.903247 | 3.12E-07 |
| GAPT | -1.5334 | 2.96878 | 0.002634 |
| A2M | -1.09196 | 2.638699 | 0.001498 |
| GPX2 | -1.47381 | 1.261317 | 0.000984 |
| CYCSP34 | 1.129567 | 0.394178 | 0.007459 |
| AC110781.3 | -1.23282 | 1.078589 | 0.00364 |
| CASKIN2 | -1.25207 | 1.386344 | 0.00053 |
| AC024940.1 | -2.04446 | 1.022104 | 0.000165 |
| ZFAS1 | -1.15612 | 5.188985 | 4.24E-18 |
| CDKN2AIPNLP1 | -1.06413 | 1.345111 | 0.003317 |
| ODF3 | -2.69143 | 0.31928 | 5.93E-06 |
| RPS27 | -1.02439 | 6.29016 | 1.91E-12 |
| DDC8 | -2.0272 | -0.1436 | 0.00022 |
| COX14 | -1.10165 | 4.895757 | 2.03E-14 |
| MCMDC2 | -1.06896 | 4.339868 | 1.14E-11 |
| FCER1A | -1.82787 | 4.879404 | 0.000749 |
| C1orf200 | -1.7748 | 1.998067 | 1.59E-05 |
| RP1-278E11.3 | -1.37394 | 9.890544 | 1.75E-34 |
| ATP5EP2 | -1.39016 | 8.440694 | 7.94E-28 |
| HIST1H2BC | -1.2957 | 0.946947 | 0.003328 |
| RPL24P4 | -1.0409 | 9.304836 | 1.74E-20 |
| SNRPE | -1.05969 | 4.049882 | 1.22E-09 |
| CRIP1P4 | -1.09546 | 4.90448 | 4.06E-11 |
| NXPH3 | -3.76127 | 0.055996 | 3.24E-06 |
| RPL35A | -1.16582 | 8.295786 | 3.75E-24 |
| PNMA3 | 1.085532 | 4.567283 | 6.02E-07 |
| UQCR10 | -1.04058 | 5.624555 | 1.33E-16 |
| RP11-44M6.3 | -1.49555 | 0.317166 | 0.000945 |
| BEX5 | -1.10359 | 2.185706 | 0.001472 |
| FAM110C | 1.43787 | 0.428953 | 0.003454 |
| NDUFA12 | -1.34656 | 4.922236 | 7.56E-22 |
| APOO | -1.25386 | 0.749945 | 0.005904 |
| MUC6 | -2.45797 | 1.975728 | 1.14E-08 |
| WBP5 | -1.06964 | 0.908336 | 0.00319 |
| STAC3 | -1.10909 | 1.375456 | 0.001642 |
| CTD-2287O16.1 | -1.05755 | 9.144175 | 1.35E-15 |
| ADARB2 | 2.47095 | 1.453553 | 0.006865 |
| RPL12P4 | -1.25255 | 6.245447 | 2.36E-20 |
| FOXD2 | 1.094335 | 1.480129 | 0.001009 |
| HIST1H2BG | -1.1702 | 1.948998 | 0.003425 |
| COMMD6 | -1.31605 | 6.331594 | 1.28E-24 |
| C19orf35 | -1.57118 | 1.844997 | 4.29E-05 |
| SUMO2 | -1.086 | 4.900689 | 3.18E-12 |
| FAM26F | -1.41635 | 2.79789 | 0.000589 |
| HIST1H4D | -2.83401 | -0.3608 | 8.44E-05 |
| HLA-DRB1 | -1.23257 | 5.314716 | 0.000647 |
| TOMM7 | -1.3278 | 7.491088 | 3.89E-25 |
| CHRNG | -1.53372 | 0.655914 | 0.002413 |
| HIST1H4C | -2.14424 | 0.077871 | 0.000321 |
| HIST1H3D | -1.62715 | 0.628964 | 0.006744 |
| PRAMENP | -2.24625 | 0.280049 | 0.001037 |
| ZNF460 | -1.57427 | 3.926034 | 7.12E-15 |
| RPL37A | -1.1158 | 6.882362 | 4.77E-15 |
| HMGN5 | -1.1427 | 1.281077 | 0.005133 |
| RPL23A | -1.03825 | 6.075609 | 5.76E-21 |
| HLA-DRB5 | 2.659275 | 2.01067 | 0.001551 |
| MIR342 | -1.48929 | 1.759962 | 3.51E-05 |
| RNU5E-1 | -1.84435 | 0.037548 | 0.005613 |
| RNU5F-1 | -2.16625 | 0.402379 | 0.001589 |
| RNU5A-1 | -1.09013 | 3.119926 | 0.005623 |
| SNORA73B | -2.01086 | 2.9421 | 1.15E-12 |
| SNORD14E | -1.04239 | 1.951638 | 0.00611 |
| RNVU1-6 | -1.47452 | 0.511609 | 0.007006 |
| RNU1-59P | -2.24156 | 0.455702 | 0.002175 |
| RNU1-60P | -1.77495 | 2.962575 | 7.76E-06 |
| RN7SK | -3.25353 | 5.762512 | 2.82E-31 |
| Y | 1.50661 | 0.767231 | 0.000111 |
| RNU4-2 | -2.27271 | 3.716872 | 1.79E-06 |
| OR1F2P | -1.57291 | 0.80849 | 0.001792 |
| ADAMTSL4-AS1 | -1.23512 | 0.357027 | 0.005051 |
| PFN1P2 | 1.913516 | -0.1322 | 0.000224 |
| HLA-DRA | -1.38822 | 7.828769 | 0.000254 |
| CARD16 | -1.08742 | 3.847013 | 1.14E-07 |
| AIF1 | -1.16427 | 4.098965 | 2.55E-06 |
| FAM196B | -1.58229 | 0.491127 | 0.004764 |
| COX17P1 | -1.02063 | 3.986372 | 3.90E-09 |
| AC016586.1 | -1.39999 | 1.332537 | 0.000259 |
| MUC12 | -1.79875 | 0.36058 | 9.14E-05 |
| TMEM256 | -1.21667 | 3.150242 | 8.23E-07 |
| SNORD15B | -1.95891 | 1.293287 | 4.70E-05 |
| RNVU1-14 | -1.29314 | 2.931796 | 0.000433 |
| MT-RNR2 | -1.01751 | 12.66285 | 0.000309 |
| MT-RNR1 | -1.36473 | 10.84945 | 0.000147 |
| AC118278.1 | 1.194258 | 0.842586 | 0.005389 |
| MIR762 | 2.044954 | 0.683997 | 0.00019 |
| TRBV25-1 | -1.21521 | 2.761888 | 5.94E-05 |
| TRAJ29 | -1.89444 | 0.59961 | 6.48E-07 |
| IGHV3-66 | -3.30819 | 3.005892 | 0.007379 |
| U3 | -1.91053 | 1.377735 | 0.005015 |
| SNORD17 | -2.20245 | 1.630907 | 4.95E-10 |
| SNORA53 | -1.9429 | 0.439874 | 0.001428 |
| RP4-765C7.2 | -1.45231 | 2.682034 | 3.43E-06 |
| RP11-613F7.1 | -1.10983 | 1.860579 | 0.00108 |
| RP11-641D5.1 | -1.05933 | 8.993749 | 5.05E-23 |
| RPS7P11 | -1.07294 | 1.989938 | 8.69E-05 |
| AKR1B1P2 | -1.19977 | 1.47023 | 0.004048 |
| RP11-112J1.1 | -1.24528 | 9.38034 | 1.58E-21 |
| RP11-380G5.3 | -1.20727 | 3.393551 | 5.16E-09 |
| ANAPC10P1 | -1.11233 | 2.554831 | 8.87E-05 |
| KLRK1 | 1.07784 | 1.122607 | 0.007007 |
| RP11-829H16.2 | -1.25764 | 1.179201 | 0.001014 |
| NPM1P33 | -1.29664 | 0.156546 | 0.007662 |
| AC092610.12 | -1.10961 | 3.210758 | 1.10E-05 |
| RP1-102E24.1 | -1.28392 | 0.989835 | 0.00131 |
| RP11-247I13.3 | -1.72471 | 0.197224 | 0.002343 |
| AC010336.1 | -1.63648 | 0.359529 | 0.003572 |
| CTB-13H5.1 | -1.07347 | 4.527152 | 1.46E-12 |
| RPS15AP1 | -1.16974 | 9.488568 | 1.53E-18 |
| RPS19P1 | -1.15791 | 6.969546 | 1.52E-23 |
| AC010468.1 | -1.13478 | 3.620014 | 1.17E-05 |
| SHFM1P1 | -1.09732 | 3.748226 | 1.67E-09 |
| RP11-1023L17.2 | -1.8396 | 0.88544 | 0.00046 |
| FAM66B | 2.11958 | -0.29448 | 0.001042 |
| BCRP3 | 2.080619 | -0.39528 | 0.000403 |
| TTC34 | -1.08011 | 2.27645 | 0.000192 |
| RP1-28C20.1 | -1.28451 | 0.351082 | 0.00363 |
| RP11-204C16.4 | 1.093958 | 0.897133 | 0.000396 |
| RP3-340B19.2 | -1.50501 | 4.74281 | 6.56E-20 |
| RPL23P8 | -1.27542 | 7.969061 | 1.22E-27 |
| RP11-40C6.2 | -1.51397 | 7.505835 | 6.20E-20 |
| RP11-572P18.1 | -1.54905 | 5.031474 | 1.70E-16 |
| MRPS21P3 | -1.88627 | 0.692842 | 0.005041 |
| TMEM14E | -1.906 | 0.93222 | 0.000117 |
| AL662800.1 | -1.71058 | 0.703418 | 0.001624 |
| RP11-545E17.3 | -1.07183 | 2.509513 | 6.19E-05 |
| ATP6V0E1P2 | -1.29741 | 2.090856 | 4.58E-05 |
| AC093690.1 | -1.32014 | 1.582335 | 0.000127 |
| TMSB4XP4 | -1.32091 | 1.995817 | 0.000121 |
| RP3-476K8.4 | -2.60065 | 0.101506 | 8.53E-05 |
| RPS20P14 | -1.19023 | 8.563267 | 4.80E-24 |
| AC013733.5 | -1.01648 | 4.492121 | 3.91E-11 |
| RP11-343H5.4 | -1.19103 | 5.895321 | 1.18E-06 |
| HAUS1P2 | -1.47463 | 0.387581 | 0.00044 |
| THAP5P1 | 1.148058 | 0.894533 | 0.005704 |
| AC017035.2 | -1.76326 | 0.250703 | 0.001789 |
| RP11-439L18.2 | 1.034515 | 1.204811 | 0.005889 |
| RP11-270C12.3 | -1.21723 | 6.694206 | 1.53E-18 |
| RP11-51O6.1 | -1.1115 | 10.40685 | 1.15E-23 |
| RP4-796I17.5 | -1.13844 | 3.220713 | 5.48E-08 |
| AB019441.29 | -1.22852 | 6.493497 | 4.51E-28 |
| AP001055.6 | 1.275299 | 1.92605 | 5.72E-05 |
| RP11-262D11.2 | -1.0919 | 8.24964 | 3.50E-18 |
| RPL35P5 | -1.26005 | 7.582171 | 1.01E-27 |
| EDNRB-AS1 | -1.69109 | -0.41115 | 0.002171 |
| RP4-604A21.1 | -1.34509 | 4.912832 | 3.00E-12 |
| HNRNPA3P15 | -1.66055 | -0.15059 | 0.007275 |
| AC092933.3 | -1.2556 | 3.874448 | 1.01E-09 |
| RP13-258O15.1 | -1.20224 | 3.035127 | 2.02E-06 |
| RP3-340N1.5 | -1.38148 | 0.916912 | 0.003569 |
| RP4-706A16.3 | -1.15548 | 4.772093 | 3.85E-13 |
| AC022431.1 | -1.22946 | 9.678083 | 1.65E-27 |
| RPL37AP1 | -1.28139 | 9.264543 | 1.16E-34 |
| ARPC3P1 | -1.06921 | 3.515807 | 1.34E-08 |
| MIR29B1 | -1.47951 | 2.625697 | 3.26E-07 |
| RPS7P10 | -1.03061 | 5.959737 | 4.40E-12 |
| RP11-115D7.3 | -1.11257 | 1.711967 | 0.001185 |
| TBCAP1 | -1.04177 | 6.173747 | 5.75E-14 |
| RP11-166N17.3 | -1.53954 | 0.40227 | 0.006437 |
| RP3-417G15.1 | -1.33312 | 7.483068 | 5.12E-28 |
| RP11-543P15.1 | -1.42329 | 10.14262 | 1.62E-32 |
| MYL6P3 | -1.64414 | 0.079258 | 0.00152 |
| RP11-864N7.2 | -1.10755 | 11.56141 | 9.85E-21 |
| RP11-958N24.2 | -2.32306 | 0.42792 | 0.00255 |
| PIN4P1 | -1.27431 | 3.614216 | 6.13E-10 |
| RP1-197O17.2 | -1.02644 | 2.88704 | 5.43E-05 |
| LOC124685 | -1.11925 | 5.722796 | 1.31E-14 |
| OST4 | -1.04984 | 6.679748 | 1.83E-17 |
| AC096558.1 | -1.69994 | 0.759818 | 0.000394 |
| RP11-249L21.4 | -1.15771 | 5.043629 | 3.15E-15 |
| ATP5G2P4 | -1.05668 | 5.550306 | 3.15E-21 |
| RP11-57H12.5 | 2.010168 | 0.120207 | 0.000199 |
| RP11-159C21.4 | -1.06675 | 10.33102 | 2.13E-25 |
| SNRPD2P1 | -1.08233 | 6.235883 | 5.19E-17 |
| RP11-378J18.6 | -1.17496 | 4.362499 | 6.96E-11 |
| RPL35AP21 | -1.02096 | 2.418325 | 0.002027 |
| RP5-1049G16.4 | -2.56982 | 0.119785 | 7.08E-05 |
| AC092431.2 | -1.73215 | 0.224741 | 0.002261 |
| MIR181A1HG | -2.08403 | 0.336422 | 0.001596 |
| TMSB4XP6 | -1.37314 | 2.568877 | 8.44E-07 |
| RP11-244J10.1 | -1.30863 | 3.946534 | 1.68E-06 |
| RP3-323N1.2 | -1.78508 | 0.86996 | 0.000975 |
| U73166.2 | -1.26646 | 1.486284 | 0.000199 |
| RPS23P8 | -1.30109 | 10.23932 | 9.44E-32 |
| RP11-409K20.6 | -1.9732 | -0.17403 | 0.001401 |
| RP11-296L22.8 | -2.13286 | 0.897825 | 0.00083 |
| AC127904.2 | -1.20036 | 0.497657 | 0.002339 |
| RP11-318C24.1 | -1.31887 | 4.359228 | 9.44E-05 |
| RPS18P12 | -1.06513 | 4.875451 | 2.96E-05 |
| RP3-426I6.2 | -1.37242 | 1.49239 | 0.000554 |
| AC006028.11 | -1.1899 | 2.212556 | 0.000204 |
| RP1-40G4P.1 | -1.00246 | 0.426467 | 0.007054 |
| HLA-DPA1 | -1.07357 | 7.170784 | 0.000307 |
| RP11-422P24.9 | -1.28692 | 7.192299 | 2.56E-13 |
| HINT1P1 | -1.2737 | 4.79475 | 1.95E-17 |
| USMG5P1 | -1.48328 | 5.424659 | 1.61E-26 |
| DLEU2 | -1.14613 | 2.180693 | 7.35E-05 |
| AC013264.2 | -1.46905 | 3.897006 | 2.03E-07 |
| MIR4426 | -1.16377 | 5.36215 | 4.11E-11 |
| RP11-558F24.4 | -1.21031 | 1.306467 | 0.000303 |
| RP13-93L13.2 | -1.23568 | 1.468876 | 0.002567 |
| SIK3-IT1 | -1.07982 | 0.983024 | 0.005968 |
| SNRPFP1 | -1.3648 | 3.985587 | 4.60E-17 |
| NDUFB1P1 | -1.25843 | 4.958835 | 1.31E-13 |
| TPRKBP1 | -1.15408 | 0.886257 | 0.001341 |
| TMA7 | -1.07515 | 4.556958 | 8.83E-14 |
| ATP5LP2 | -1.17957 | 5.181371 | 2.84E-13 |
| LINC00493 | -1.03389 | 4.911643 | 3.50E-12 |
| RP4-591N18.2 | -1.83136 | 0.271014 | 0.001753 |
| EEF1A1P3 | -2.75751 | 0.521621 | 1.99E-05 |
| GSTO3P | -1.04392 | 3.072041 | 1.58E-06 |
| RPL34P27 | -1.01866 | 2.444868 | 0.000962 |
| RPS11P5 | -1.00222 | 10.80844 | 5.88E-22 |
| RP11-558F24.2 | -2.5635 | 0.184838 | 9.68E-05 |
| SNRPEP4 | -1.15537 | 4.281494 | 8.88E-10 |
| BTBD18 | -1.20722 | 0.921301 | 0.001649 |
| RP1-187B23.1 | -1.5297 | 0.892455 | 0.007365 |
| RP11-570P14.1 | -1.0494 | 4.785805 | 4.16E-13 |
| AC017104.2 | -2.02992 | 0.475933 | 0.000953 |
| AC016831.7 | -2.81749 | 0.604452 | 4.80E-06 |
| BTF3P5 | -1.07533 | 1.804627 | 0.000159 |
| CYP2T2P | -1.09905 | 1.464776 | 0.000672 |
| RP11-389O22.4 | -1.54179 | 0.802743 | 0.007649 |
| RP11-169K16.7 | -1.22711 | 6.671674 | 2.23E-20 |
| RP5-887A10.1 | -3.19646 | 0.359234 | 0.002003 |
| RP11-454L1.2 | -1.68303 | -0.31035 | 0.005665 |
| RP11-761N21.2 | -1.47985 | 6.797338 | 2.53E-26 |
| AC096664.1 | -2.67186 | 0.216837 | 3.69E-06 |
| AC114730.7 | 1.003716 | 3.113506 | 0.000139 |
| RPL39P3 | -1.18734 | 10.69953 | 3.85E-22 |
| CTA-276O3.4 | -1.27806 | 4.338407 | 1.08E-09 |
| SNRPGP10 | -1.26432 | 1.949976 | 0.000278 |
| RP11-480I12.2 | -1.11641 | 5.025171 | 5.49E-12 |
| AC092580.4 | -1.07342 | 2.186007 | 0.002268 |
| AL109767.1 | -1.02921 | 1.529498 | 0.00601 |
| RP1-159A19.3 | -1.33273 | 2.33812 | 3.05E-06 |
| RP13-444K19.1 | -1.58136 | 3.137845 | 2.59E-13 |
| COX7CP1 | -1.4259 | 3.504774 | 1.06E-12 |
| RP1-3J17.3 | -2.92058 | -0.06936 | 7.65E-05 |
| MRPS36P1 | -1.08215 | 3.736519 | 3.80E-09 |
| AC104651.1 | -1.23938 | 1.118206 | 0.005936 |
| RPL31P63 | -1.67532 | 1.557704 | 4.38E-07 |
| COX7A2P2 | -1.19313 | 5.63583 | 6.94E-14 |
| RPL24P8 | -1.07348 | 8.160277 | 3.25E-21 |
| ANKRD44-IT1 | -1.404 | 1.860733 | 5.27E-05 |
| AP000361.2 | -1.42174 | 2.094778 | 9.44E-07 |
| RPS15AP38 | -1.61905 | 1.502689 | 1.25E-06 |
| RPL30P4 | -1.15333 | 8.026221 | 3.76E-19 |
| RP11-459A10.1 | -1.3549 | 0.801614 | 0.003723 |
| UQCRBP1 | -1.3588 | 5.295248 | 2.43E-19 |
| AC116366.5 | -1.81663 | 0.754635 | 0.000174 |
| SCARNA7 | -3.15658 | 0.678873 | 3.11E-09 |
| SCARNA10 | -2.93933 | 2.388417 | 2.54E-11 |
| SNORD13 | -2.52472 | 0.305437 | 0.001958 |
| RPL34P31 | -1.25926 | 2.828252 | 5.29E-06 |
| RP11-464D20.2 | -1.02007 | 3.603339 | 4.17E-07 |
| RP11-771F20.1 | -1.25069 | 0.470564 | 0.004798 |
| RPL37P2 | -1.15553 | 5.287086 | 1.37E-17 |
| NME1 | -1.04402 | 3.287082 | 2.21E-05 |
| RP4-800G7.1 | -1.71471 | 1.329322 | 4.07E-05 |
| IGKV1-6 | -2.15493 | 2.119477 | 0.002145 |
| RPL34P18 | -1.2797 | 9.088614 | 3.08E-20 |
| RP11-3P17.4 | -1.51578 | 2.987543 | 7.48E-12 |
| RP11-488C13.1 | -1.22719 | 8.17266 | 5.56E-31 |
| RP5-1051J4.4 | 2.362715 | -0.41991 | 6.52E-05 |
| RP1-89D4.1 | -1.44859 | 1.18344 | 0.000314 |
| PDXP | 1.300569 | 0.903884 | 0.000887 |
| RP11-796G6.1 | -1.18912 | 4.34266 | 1.86E-12 |
| RPL31P49 | -1.03463 | 1.753124 | 0.000964 |
| ZNF90P1 | -1.63668 | 0.036568 | 0.003439 |
| RPS21P4 | -1.19705 | 9.384489 | 1.08E-25 |
| RP11-601I15.1 | -1.25066 | 2.671146 | 2.53E-05 |
| RP11-436H11.1 | -1.17219 | 5.066779 | 3.60E-11 |
| MRPL33 | -1.14087 | 4.261146 | 1.31E-11 |
| UBA52P5 | -1.00228 | 5.28374 | 3.95E-11 |
| RPS23P1 | -1.27857 | 6.528964 | 5.70E-23 |
| RPL37P23 | -1.26097 | 6.861061 | 2.93E-22 |
| RP11-390K5.1 | -1.7729 | 0.385021 | 0.000349 |
| RPS27P27 | -1.11643 | 3.644525 | 4.89E-10 |
| RP1-93H18.6 | -2.16017 | 1.343706 | 0.000247 |
| RP11-114H7.1 | -1.09843 | 9.014549 | 1.22E-24 |
| RP11-466H18.1 | -1.31071 | 9.869869 | 4.62E-26 |
| RP1-181J22.1 | -1.59277 | 1.073247 | 0.001107 |
| RP11-20O24.4 | -1.006 | 5.065668 | 5.13E-12 |
| KB-1208A12.3 | -1.06715 | 8.021876 | 1.52E-23 |
| CTD-2024I7.13 | -2.0428 | 0.114139 | 0.00032 |
| CICP16 | 1.316666 | 0.908052 | 0.000142 |
| POU5F2 | -1.69012 | 0.118604 | 0.000992 |
| CTD-2206G10.2 | -1.15938 | 8.061016 | 1.20E-21 |
| RP11-616K22.1 | -1.72036 | 0.154922 | 0.000415 |
| RP11-478C6.4 | -1.58277 | 6.124308 | 5.36E-35 |
| RP11-47I22.2 | -2.07145 | -0.25612 | 0.006522 |
| RP11-503N18.5 | -1.67552 | 0.927586 | 0.000157 |
| CTB-109A12.1 | -1.95209 | 1.807272 | 3.24E-07 |
| FKBP4P1 | -1.37518 | 0.26257 | 0.005493 |
| MALAT1 | -2.24387 | 12.50249 | 3.33E-84 |
| RP11-539L10.3 | -1.07711 | 1.732333 | 0.007573 |
| SCARNA6 | -1.31963 | 1.657006 | 0.000173 |
| SCARNA17 | -2.59497 | 2.577048 | 8.57E-14 |
| SCARNA5 | -2.89548 | 2.323649 | 1.52E-09 |
| SCARNA18 | -2.64812 | 1.511919 | 3.65E-08 |
| SCARNA21 | -2.1909 | 1.964008 | 4.07E-10 |
| Y | -1.53812 | -0.07317 | 0.007317 |
| IGLVI-70 | -3.30256 | 1.107411 | 1.31E-05 |
| CTC-248O19.1 | 1.293304 | 2.357097 | 5.05E-05 |
| CTB-79E8.3 | -1.03162 | 6.507251 | 3.47E-19 |
| RP11-473O4.3 | -2.26204 | -0.03753 | 0.000407 |
| RP11-172E10.1 | -2.56586 | 0.035383 | 0.000359 |
| RP11-34P1.2 | -1.2206 | 3.988658 | 1.91E-08 |
| SCARNA9 | -1.91706 | 3.77345 | 3.01E-15 |
| RP11-58K22.4 | -2.67137 | -0.3936 | 0.007551 |
| RP11-685M7.5 | -1.17846 | 1.627533 | 0.000997 |
| RP11-841C19.3 | -1.12463 | 2.840001 | 2.98E-06 |
| TAS2R30 | -2.89934 | -0.12375 | 0.000516 |
| RP11-664D1.1 | -1.143 | 4.356419 | 6.58E-11 |
| RP1-102E24.6 | -1.5107 | 0.847613 | 0.006336 |
| SNRPEP2 | -1.32156 | 2.968847 | 3.60E-09 |
| RP11-609L23.2 | -1.11187 | 1.240833 | 0.001451 |
| RP11-570L15.2 | -2.34113 | -0.30397 | 0.005025 |
| RP11-530C5.1 | 2.371644 | -0.4752 | 1.92E-05 |
| RN7SL1 | -2.13138 | 5.076577 | 3.92E-10 |
| RP11-638I2.10 | -1.23517 | 3.822154 | 3.35E-08 |
| AE000660.4 | 1.980662 | -0.82331 | 0.003799 |
| RP11-644F5.15 | -1.37777 | 1.115439 | 0.000846 |
| RPPH1 | -2.42145 | 3.555485 | 2.58E-08 |
| CAP2P1 | -2.69526 | -0.2053 | 0.000123 |
| PRKXP1 | 1.200074 | 4.265587 | 2.69E-08 |
| RP11-526I2.1 | 1.122414 | 2.648585 | 1.98E-05 |
| CTD-3065B20.1 | -1.26894 | 1.187944 | 5.35E-05 |
| RP11-429P3.3 | 1.029331 | 1.320303 | 9.04E-05 |
| RP11-44F14.5 | -1.91235 | -0.06144 | 2.56E-05 |
| RP11-304L19.1 | 1.030615 | 2.269128 | 3.05E-05 |
| RP11-266L9.2 | -1.14512 | 1.41673 | 0.001982 |
| COX6CP1 | -1.1496 | 1.919482 | 0.000848 |
| RP5-1085F17.4 | -1.69469 | -0.16181 | 0.002657 |
| RP11-474B12.1 | -3.25314 | -0.22206 | 0.003765 |
| SUB1P3 | -1.13873 | 2.975259 | 2.56E-06 |
| RP11-328J14.2 | -1.48634 | 0.405138 | 0.002224 |
| RP11-104O19.2 | -1.302 | 0.979013 | 0.002325 |
| CTD-2561B21.5 | -1.822 | 1.446117 | 1.61E-08 |
| CTD-2561B21.11 | -1.26544 | 1.282299 | 0.00185 |
| TAPT1-AS1 | -1.21221 | 9.348224 | 3.37E-28 |
| MIR4665 | 1.121674 | 1.217046 | 0.001152 |
| RP11-498C9.15 | -1.14883 | 2.157699 | 8.51E-05 |
| RP11-321A17.3 | -1.24811 | 4.735884 | 1.01E-15 |
| RNU4ATAC | -2.21086 | 0.683871 | 0.003399 |
| SNRPGP2 | -1.42006 | 2.812575 | 4.50E-06 |
| RP4-777O23.2 | 1.56472 | 0.376362 | 0.001671 |
| RP11-4F22.2 | -2.55005 | -0.10641 | 0.000147 |
| MIR4523 | 1.207314 | 1.119428 | 0.003469 |
| RP11-16C1.2 | -2.58553 | 0.218023 | 3.82E-05 |
| RP11-6N17.4 | -1.14442 | 1.305877 | 0.001629 |
| RN7SL2 | -2.29851 | 5.221759 | 6.68E-16 |
| MIR151B | -2.12124 | 0.640169 | 0.000119 |
| MIR3609 | -3.54438 | 1.180089 | 1.62E-10 |
| UBL5P2 | -1.2415 | 5.863148 | 2.15E-25 |
| CTC-507E2.1 | -2.05023 | 0.288301 | 0.000941 |
| CTC-448F2.4 | -1.41682 | 0.072801 | 0.002132 |
| RP11-126O1.5 | 1.489976 | 2.395442 | 2.65E-07 |
| CTD-2528L19.3 | -1.20242 | 2.120129 | 7.35E-05 |
| CTC-260E6.6 | -1.08977 | 7.120632 | 6.28E-14 |
| AC007229.3 | -1.02198 | 2.740231 | 1.54E-05 |
| HMGB2P1 | -1.24165 | 1.729058 | 0.000908 |
| RP11-358B23.5 | -1.18801 | 1.720983 | 0.003891 |
| RP11-171I2.1 | -2.1408 | 0.231591 | 0.00118 |
| RP11-91G21.1 | -1.59193 | 0.660991 | 0.000444 |
| AC026202.1 | 1.023721 | 3.766937 | 3.01E-06 |
| CTC-246B18.10 | -1.593 | 0.401006 | 0.003642 |
| RP11-345P4.7 | 1.412879 | 3.450448 | 2.00E-05 |
| FLJ20306 | 1.231433 | 1.348007 | 0.005368 |
| KCNQ1OT1 | -1.11961 | 4.062272 | 1.84E-06 |
| AL627171.2 | -1.11259 | 0.571754 | 0.007404 |
| RP11-420K14.6 | -1.14163 | 0.970095 | 0.003408 |
| SNHG8 | -1.02004 | 5.745597 | 4.52E-12 |
| RMRP | -2.63174 | 4.507259 | 2.66E-11 |
| RNU12 | -1.67038 | 1.415428 | 0.001561 |
| SCARNA2 | -1.50887 | 2.068688 | 0.000128 |
| RNU11 | -2.06221 | 1.276133 | 0.003153 |
| AP000769.7 | -1.54711 | 0.803798 | 0.001993 |
| RP11-526I2.5 | 1.045261 | 4.902054 | 5.50E-05 |
| NDUFB8P2 | -1.0709 | 3.19772 | 4.44E-06 |
| RP11-15E18.5 | -1.18362 | 2.717983 | 3.97E-06 |
| RP11-138A9.1 | -3.67672 | 0.65083 | 2.81E-10 |
| RP11-392O18.2 | -1.00948 | 1.954162 | 0.007402 |
| RP11-51J9.5 | -1.34765 | 2.067188 | 0.000168 |
| RP11-215G15.5 | 1.089016 | 1.196917 | 0.005447 |
| RP5-1126H10.2 | -1.12977 | 1.068626 | 0.003798 |
| RP11-755B10.4 | -1.52872 | 1.185531 | 0.002652 |
| U47924.31 | -1.01386 | 6.633373 | 6.57E-17 |
| RP11-347P5.1 | -1.39048 | 2.90303 | 2.73E-07 |
| SNORA28 | -1.56769 | 0.528428 | 0.003794 |
| RP4-673M15.1 | 1.273606 | 2.012407 | 0.000272 |
| RP11-127B20.2 | -1.96466 | 0.882591 | 0.000141 |
| DGCR11 | -1.10974 | 2.068247 | 0.001032 |
| RP11-138A9.2 | -3.29979 | 2.292793 | 5.47E-18 |
| RP11-386I14.4 | -2.20641 | 3.148054 | 1.31E-07 |
