# Supplementary material for: CD4+ T cells from children with active juvenile idiopathic arthritis show altered chromatin features associated with transcriptional abnormalities
Source: Sci Rep. 2021 Feb 17;11:4011. doi: 10.1038/s41598-021-82989-5 (PMC7889855; doi:10.1038/s41598-021-82989-5)
Supplement: Supplementary file 5 — Supplementary Table 3. [file 41598_2021_82989_MOESM5_ESM.docx]

**Table S3**

Differentially expressed genes between CRM and HC, whose log10(Fold-change) was at least 1 and whose FDR was less than or equal to 0.05.

| **Gene Names** | **Log(fold-change)** | **Log(CPM)** | **P-Value** |
| --- | --- | --- | --- |
| ABCB4 | 2.451692 | -0.13466 | 0.000141 |
| CD22 | 2.330256 | 3.597483 | 4.51E-05 |
| CDH1 | 1.704096 | 0.758357 | 4.23E-05 |
| ADAM28 | 1.677257 | 3.527185 | 6.77E-06 |
| COL9A2 | 1.302484 | 2.264879 | 2.27E-05 |
| ST6GALNAC1 | -1.32875 | 3.989274 | 9.94E-09 |
| CYBRD1 | 1.923861 | 2.490536 | 7.00E-05 |
| NME8 | 2.856622 | -0.04595 | 2.16E-06 |
| TCL1A | 2.413211 | 3.113289 | 2.68E-05 |
| DRP2 | -1.70625 | 0.048901 | 0.000115 |
| TSPAN13 | 2.154653 | 1.911002 | 7.04E-06 |
| P2RX1 | 1.282889 | 3.323389 | 0.000156 |
| DTX4 | 1.232687 | 3.206801 | 5.35E-05 |
| C1orf21 | 1.628879 | 2.882737 | 4.46E-05 |
| BCL11A | 1.391018 | 2.923643 | 0.000127 |
| EGR1 | 3.434838 | 5.283352 | 1.83E-05 |
| FLT3 | 1.725544 | 2.238237 | 5.32E-05 |
| NR4A1 | 4.67159 | 4.892409 | 1.22E-07 |
| WDFY4 | 1.239197 | 4.834289 | 0.000134 |
| PMAIP1 | 1.597903 | 3.805862 | 0.00011 |
| SCD5 | 2.842991 | 0.850973 | 3.73E-09 |
| MS4A1 | 2.33372 | 4.514863 | 2.93E-06 |
| FCRL1 | 1.698479 | 4.367163 | 0.000122 |
| FAM129C | 2.229608 | 3.726873 | 2.02E-07 |
| LILRA3 | 4.463572 | 0.93577 | 9.24E-05 |
| MCC | 1.257684 | 3.945354 | 2.14E-05 |
| SLCO4C1 | 1.563132 | 1.392203 | 3.07E-05 |
| TP53I11 | 1.581517 | 3.47011 | 1.91E-08 |
| CIITA | 1.171653 | 6.356105 | 7.81E-05 |
| FCRL6 | 1.491382 | 3.014982 | 3.69E-05 |
| KCNH8 | 2.847168 | 0.062729 | 6.71E-05 |
| RBM11 | -1.01297 | 2.377362 | 7.30E-05 |
| AHNAK2 | 2.226249 | 0.909474 | 0.000137 |
| NAT8L | 2.479456 | 0.163218 | 1.11E-06 |
| H1FNT | 1.126545 | 0.355657 | 3.34E-05 |
| TCL6 | 2.167175 | 1.185078 | 7.57E-06 |
| PAX5 | 2.223014 | 3.53242 | 9.32E-05 |
| HLA-DOA | 1.390194 | 4.695347 | 4.99E-06 |
| HLA-DMA | 1.232283 | 5.500462 | 3.43E-06 |
| HSPA1B | 3.687803 | 6.25489 | 6.22E-07 |
| RP11-693J15.5 | 2.600038 | 1.110064 | 1.79E-05 |
| GPR56 | 1.688388 | 4.653427 | 2.18E-05 |
| KLRC2 | 2.725068 | 0.012881 | 4.10E-05 |
| TRGC1 | 1.353693 | 4.027442 | 1.39E-06 |
| TRGV9 | 1.971141 | 2.382609 | 1.09E-05 |
| HLA-DPB1 | 1.344249 | 6.666131 | 8.57E-05 |
| HLA-DPA1 | 1.333689 | 7.368036 | 0.00014 |
| hsa-mir-6723 | -3.89617 | 1.048022 | 6.27E-07 |
| HLA-DMB | 1.64139 | 5.238181 | 4.61E-06 |
| LINC00926 | 2.244886 | 4.442018 | 4.70E-11 |
| RP11-1100L3.8 | 4.134908 | 2.994697 | 5.47E-05 |
| MILR1 | 2.529004 | 0.030231 | 4.70E-05 |
