# Supplementary material for: CD4+ T cells from children with active juvenile idiopathic arthritis show altered chromatin features associated with transcriptional abnormalities
Source: Sci Rep. 2021 Feb 17;11:4011. doi: 10.1038/s41598-021-82989-5 (PMC7889855; doi:10.1038/s41598-021-82989-5)
Supplement: Supplementary file 6 — Supplementary Table 4. [file 41598_2021_82989_MOESM6_ESM.docx]

**Table S4**

Differentially Expressed Genes and their cluster assignments from Figure 1B.

| **Gene Name** | **Cluster** |
| --- | --- |
| RP11-293G6 | 1 |
| RP11-293G6 | 1 |
| SCARNA17 | 1 |
| SCARNA18 | 1 |
| SCARNA17 | 1 |
| SCARNA17 | 1 |
| SCARNA18 | 1 |
| SCARNA17 | 1 |
| SCARNA18 | 1 |
| SCARNA18 | 1 |
| SCARNA6 | 1 |
| MIR4523 | 1 |
| RP11-293G6 | 1 |
| SCARNA18 | 1 |
| ADARB2 | 1 |
| RP11-478C6.4 | 1 |
| hsa-mir-6723 | 1 |
| NDUFS4 | 1 |
| RP11-122C9.1 | 1 |
| RPS7P1 | 1 |
| DNAJC19P9 | 1 |
| SNRPD2P1 | 1 |
| CA6 | 1 |
| RPS8 | 1 |
| RP11-442A13.1 | 1 |
| CTC-268N12.2 | 1 |
| RP11-343H5.4 | 1 |
| KCNJ13 | 1 |
| RLN2 | 1 |
| MIR151B | 1 |
| AC010240.2 | 1 |
| RPS3AP26 | 1 |
| AC092610.12 | 1 |
| RPL32 | 1 |
| RPS15AP24 | 1 |
| SCARNA21 | 1 |
| AC079922.2 | 1 |
| RPL24P4 | 1 |
| RNVU1-14 | 1 |
| COX7A2 | 1 |
| NDUFS5 | 1 |
| NGFRAP1 | 1 |
| CTD-2270N23.1 | 1 |
| RP11-234A1.1 | 1 |
| C12orf57 | 1 |
| RP11-6N17.4 | 1 |
| RPS4X | 1 |
| RP11-112J1.1 | 1 |
| RPS16 | 1 |
| RP11-36C20.1 | 1 |
| RP11-425L10.1 | 1 |
| RPS7P10 | 1 |
| U47924.31 | 1 |
| RPL34P34 | 1 |
| TSPAN1 | 1 |
| RPL29 | 1 |
| RNU5A-1 | 1 |
| USMG5P1 | 1 |
| AC013474.4 | 1 |
| RPS19P1 | 1 |
| SCARNA9 | 1 |
| RPS7P11 | 1 |
| SNHG8 | 1 |
| RP11-791G16.2 | 1 |
| RP11-380G5.3 | 1 |
| COMMD6 | 1 |
| LINC00493 | 1 |
| RPL35P5 | 1 |
| CTD-2287O16.1 | 1 |
| RP11-664D1.1 | 1 |
| RP11-841C19.3 | 1 |
| SMIM11P1 | 1 |
| CTB-79E8.3 | 1 |
| RP11-507E23.1 | 1 |
| RP11-262D11.2 | 1 |
| RPS3AP6 | 1 |
| RP3-340B19.2 | 1 |
| RP4-800G7.1 | 1 |
| MYLPF | 1 |
| RP11-533O20.2 | 1 |
| AC092933.3 | 1 |
| RP11-436H11.1 | 1 |
| RPL38 | 1 |
| RP11-47F1.1 | 1 |
| RPL39P3 | 1 |
| C11orf1 | 1 |
| RPL37A | 1 |
| LINC00116 | 1 |
| RPLP2 | 1 |
| CD52 | 1 |
| RP11-378J18.6 | 1 |
| TRBV25-1 | 1 |
| DRP2 | 1 |
| RP11-159C21.4 | 1 |
| CTD-2161E19.1 | 1 |
| ST6GALNAC1 | 1 |
| ATP5L | 1 |
| AB019441.29 | 1 |
| RP11-16F15.1 | 1 |
| RP11-464D20.2 | 1 |
| AC007229.3 | 1 |
| RPL35 | 1 |
| RP11-51O6.1 | 1 |
| RPL34P18 | 1 |
| RP11-864N7.2 | 1 |
| RP4-706A16.3 | 1 |
| RPL23A | 1 |
| RPL34P31 | 1 |
| RP11-543P15.1 | 1 |
| RP11-563N4.1 | 1 |
| RPS20P10 | 1 |
| RP11-3P17.5 | 1 |
| SNORD14E | 1 |
| UQCRBP1 | 1 |
| RPL30P4 | 1 |
| RPL31P49 | 1 |
| RPL11 | 1 |
| RP11-102M11.1 | 1 |
| SNORD13 | 1 |
| RPL37AP8 | 1 |
| RP11-39H3.1 | 1 |
| CTB-13H5.1 | 1 |
| NEDD8 | 1 |
| SNRPEP2 | 1 |
| KCNS1 | 1 |
| RPS27A | 1 |
| RPS24 | 1 |
| CPA5 | 1 |
| AC013264.2 | 1 |
| PIP5KL1 | 1 |
| C14orf2 | 1 |
| RPS15AP1 | 1 |
| GUSBP5 | 1 |
| AC004453.8 | 1 |
| RP11-20O24.4 | 1 |
| ATP5LP2 | 1 |
| RP11-488C13.1 | 1 |
| HIST1H1D | 1 |
| RPL10AP2 | 1 |
| NACA3P | 1 |
| CELA1 | 1 |
| SRP14 | 1 |
| FAU | 1 |
| RP11-474P12.3 | 1 |
| RPS11 | 1 |
| RP11-66N11.8 | 1 |
| GOLGA5P1 | 1 |
| RPS21 | 1 |
| RP11-3P17.4 | 1 |
| RPS3AP5 | 1 |
| UBA52P5 | 1 |
| RP4-796I17.5 | 1 |
| RP11-114H7.1 | 1 |
| ATP5G2P4 | 1 |
| RPL23 | 1 |
| RP3-375P9.2 | 1 |
| RP11-392O18.2 | 1 |
| GAS5-AS1 | 1 |
| TRBV3-1 | 1 |
| RP11-539L10.3 | 1 |
| RP13-258O15.1 | 1 |
| AC092580.4 | 1 |
| AC010468.1 | 1 |
| RPL12P4 | 1 |
| RPL30 | 1 |
| MCMDC2 | 1 |
| CTD-2017C7.2 | 1 |
| CTC-260E6.6 | 1 |
| RPL34 | 1 |
| RPL23P8 | 1 |
| RP1-292B18.1 | 1 |
| COX7C | 1 |
| AC104651.1 | 1 |
| RPL37P23 | 1 |
| TAS2R18 | 1 |
| RP11-34P1.2 | 1 |
| COX6C | 1 |
| SNRPD2 | 1 |
| RNVU1-15 | 1 |
| COX6CP1 | 1 |
| CTD-2206G10.2 | 1 |
| RPS20 | 1 |
| RPL35A | 1 |
| RPL24P8 | 1 |
| MIR4426 | 1 |
| RPS14 | 1 |
| SNRPGP2 | 1 |
| RP11-475C16.1 | 1 |
| MT-ND3 | 1 |
| RP11-641D5.1 | 1 |
| SNRPGP10 | 1 |
| AC022431.1 | 1 |
| RP4-765C7.2 | 1 |
| RPL36 | 1 |
| RPS18P12 | 1 |
| KB-1208A12.3 | 1 |
| RPL31 | 1 |
| RPS21P4 | 1 |
| RFESDP1 | 1 |
| RPL19 | 1 |
| RPS20P14 | 1 |
| EEF1B2P2 | 1 |
| RPL27 | 1 |
| RP11-146N23.1 | 1 |
| RP11-553K8.5 | 1 |
| RP11-761N21.2 | 1 |
| KRT2 | 1 |
| TAPT1-AS1 | 1 |
| AC005618.6 | 1 |
| PRDX2P1 | 1 |
| RP11-556K13.1 | 1 |
| RP11-40C6.2 | 1 |
| RP3-417G15.1 | 1 |
| RPL27A | 1 |
| RPS11P5 | 1 |
| RPS12 | 1 |
| SHISA2 | 1 |
| RP11-314A20.1 | 1 |
| RCOR2 | 1 |
| TRBV11-1 | 1 |
| RP11-466H18.1 | 1 |
| RPL26 | 1 |
| RP11-422P24.9 | 1 |
| TOMM7 | 1 |
| RP11-244J10.1 | 1 |
| RPL19P12 | 1 |
| TRAV39 | 1 |
| RPS23P1 | 1 |
| RPL37AP1 | 1 |
| RP11-220D10.1 | 1 |
| RP1-278E11.3 | 1 |
| TRBV29-1 | 1 |
| RPS23P8 | 1 |
| MYEOV2 | 1 |
| RPL37P2 | 1 |
| RPL31P2 | 1 |
| RP11-318C24.1 | 1 |
| RP11-293G6 | 2 |
| AL122127.2 | 2 |
| ARAP3 | 2 |
| IL18RAP | 2 |
| AL122127.4 | 2 |
| FRMPD3 | 2 |
| KIF19 | 2 |
| RP11-526I2.5 | 2 |
| SIRPB1 | 2 |
| MLC1 | 2 |
| SPON2 | 2 |
| RBM11 | 2 |
| GUCY1A3 | 2 |
| NMUR1 | 2 |
| TMEM198 | 2 |
| RNF165 | 2 |
| KIAA0087 | 2 |
| HAVCR1 | 2 |
| RP11-261P9.4 | 2 |
| SLC4A10 | 2 |
| ARSD-AS1 | 2 |
| RP5-1051J4.4 | 2 |
| RP11-345P4.7 | 2 |
| RP11-277P12.20 | 2 |
| BEND4 | 2 |
| PRSS30P | 2 |
| AC007278.3 | 2 |
| BCRP3 | 2 |
| SH2D1B | 2 |
| KLRF1 | 2 |
| RP11-20I20.4 | 2 |
| CTNNAP1 | 2 |
| HLA-DRB5 | 2 |
| MCC | 2 |
| PI4KAP1 | 2 |
| ADAM12 | 2 |
| MN1 | 2 |
| GPR27 | 2 |
| AC118278.1 | 2 |
| ATP9A | 2 |
| RP11-530C5.1 | 2 |
| THBS1 | 2 |
| FSD1L | 2 |
| hsa-mir-4537 | 2 |
| FCGBP | 2 |
| DTHD1 | 2 |
| CFH | 2 |
| INSR | 2 |
| TTC34 | 2 |
| RHOBTB1 | 2 |
| KIF26A | 2 |
| KCNC3 | 2 |
| DLG5 | 2 |
| VAV2 | 2 |
| RP11-930P14.1 | 2 |
| CD34 | 2 |
| HSPA1A | 2 |
| B3GAT1 | 2 |
| RNU6-181P | 2 |
| ANKRD24 | 2 |
| RPS27 | 2 |
| EXTL3-AS1 | 2 |
| PNMA3 | 2 |
| SRC | 2 |
| SMAD1 | 2 |
| TRGV7 | 2 |
| AIRE | 2 |
| TOR4A | 2 |
| PTGDR | 2 |
| FOXD2 | 2 |
| LGALS4 | 2 |
| TRPM2 | 2 |
| RP11-108K14.4 | 2 |
| AGAP1 | 2 |
| GFOD1 | 2 |
| F2R | 2 |
| RP11-274B18.3 | 2 |
| KLRK1 | 2 |
| C1QTNF4 | 2 |
| RP4-777O23.2 | 2 |
| SLCO4C1 | 2 |
| PTGIR | 2 |
| CTIF | 2 |
| RP11-526I2.1 | 2 |
| AC123768.4 | 2 |
| HSD17B1P1 | 2 |
| ADCY6 | 2 |
| TNFRSF13C | 2 |
| C9orf172 | 2 |
| GBAP1 | 2 |
| KCNT1 | 2 |
| CACNA1F | 2 |
| GNAO1 | 2 |
| PRKXP1 | 2 |
| SOX13 | 2 |
| PDE4A | 2 |
| XCR1 | 2 |
| SNORA12 | 2 |
| PHLDB2 | 2 |
| RP4-635E18.6 | 2 |
| RP11-10N23.2 | 2 |
| AC007278.2 | 2 |
| CCNJL | 2 |
| RP11-215G15.5 | 2 |
| EGFL7 | 2 |
| KLKB1 | 2 |
| RP11-80H8.4 | 2 |
| RP11-54F2.1 | 2 |
| AC114730.7 | 2 |
| ADIRF | 2 |
| LGR6 | 2 |
| CYCSP34 | 2 |
| PIGZ | 2 |
| AC125232.1 | 2 |
| AE000660.4 | 2 |
| CICP16 | 2 |
| AP001055.6 | 2 |
| SDC4 | 2 |
| RP11-106M3.2 | 2 |
| KDM4D | 2 |
| LINC00299 | 2 |
| PLXND1 | 2 |
| H1FNT | 2 |
| MIR4665 | 2 |
| RP11-488C13.6 | 2 |
| RP11-244F12.3 | 2 |
| VASH1 | 2 |
| RP11-439L18.2 | 2 |
| MED12L | 2 |
| GUCA1B | 2 |
| SPIRE1 | 2 |
| FAM110C | 2 |
| PFN1P2 | 2 |
| RP11-304L19.1 | 2 |
| MIR762 | 2 |
| CTD-2154B17.4 | 2 |
| AC026202.1 | 2 |
| RP11-204C16.4 | 2 |
| THAP5P1 | 2 |
| ANK1 | 2 |
| RP11-227H4.5 | 2 |
| CTD-2006H14.2 | 2 |
| ABCC11 | 2 |
| RP1-90J20.11 | 2 |
| C20ORF135 | 2 |
| PGBD1 | 2 |
| RP11-57H12.5 | 2 |
| ENPP5 | 2 |
| FLJ20306 | 2 |
| PDXP | 2 |
| FAM66B | 2 |
| RP11-927P21.4 | 2 |
| RP11-126O1.5 | 2 |
| B4GALT6 | 2 |
| RP11-429P3.3 | 2 |
| CTC-248O19.1 | 2 |
| MIR4523 | 2 |
| RP4-673M15.1 | 2 |
| AC012487.2 | 2 |
| DFNB31 | 2 |
| CES1 | 3 |
| KCNH8 | 3 |
| FOLR3 | 3 |
| HLA-DMB | 3 |
| TNFAIP2 | 3 |
| CD300E | 3 |
| CSF3R | 3 |
| MPEG1 | 3 |
| TMEM150B | 3 |
| PDGFD | 3 |
| PIK3AP1 | 3 |
| TP53I11 | 3 |
| ADM | 3 |
| RHOB | 3 |
| HSPA6 | 3 |
| SCARNA21 | 3 |
| RP11-1100L3.8 | 3 |
| DOCK5 | 3 |
| CARD16 | 3 |
| CIITA | 3 |
| HLA-DQB1 | 3 |
| FGR | 3 |
| FAM129C | 3 |
| NME8 | 3 |
| SHROOM1 | 3 |
| GAS7 | 3 |
| RASD1 | 3 |
| PLXNB2 | 3 |
| ITGAX | 3 |
| RGS1 | 3 |
| CMKLR1 | 3 |
| HLA-DRA | 3 |
| CLEC4A | 3 |
| ZEB2 | 3 |
| TMEM14C | 3 |
| FCGR3A | 3 |
| AL022328.1 | 3 |
| DTX4 | 3 |
| FOSL2 | 3 |
| SLC8A1 | 3 |
| CEBPD | 3 |
| MT-RNR2 | 3 |
| LILRA3 | 3 |
| PDK4 | 3 |
| LYN | 3 |
| LILRA6 | 3 |
| EGR1 | 3 |
| CXCR2 | 3 |
| SLC24A4 | 3 |
| LILRB1 | 3 |
| HSPA1B | 3 |
| TSPAN13 | 3 |
| ZNF385A | 3 |
| PRAM1 | 3 |
| HHEX | 3 |
| LATS2 | 3 |
| MS4A1 | 3 |
| RNU12 | 3 |
| HLA-DOA | 3 |
| CD93 | 3 |
| DMXL2 | 3 |
| CYP1B1 | 3 |
| FAM49A | 3 |
| SLC43A2 | 3 |
| NAT8L | 3 |
| AP001007.1 | 3 |
| CHST15 | 3 |
| FCRL6 | 3 |
| NR4A1 | 3 |
| B3GNT7 | 3 |
| JUN | 3 |
| SLC22A15 | 3 |
| HIST1H2BG | 3 |
| CREB5 | 3 |
| BCL11A | 3 |
| HSPA7 | 3 |
| SIGLEC9 | 3 |
| CYBRD1 | 3 |
| HLA-DMA | 3 |
| MILR1 | 3 |
| SPINK2 | 3 |
| HLA-DPB1 | 3 |
| MT-RNR1 | 3 |
| IL18 | 3 |
| CD22 | 3 |
| PAX5 | 3 |
| DNASE1L3 | 3 |
| LGALS1 | 3 |
| CTD-3187F8.14 | 3 |
| MS4A14 | 3 |
| HLA-DPA1 | 3 |
| KLRC2 | 3 |
| GPR56 | 3 |
| PPM1J | 3 |
| FCRL1 | 3 |
| HIST1H2BC | 3 |
| LINC00926 | 3 |
| GAPT | 3 |
| ENHO | 3 |
| LRP1 | 3 |
| WDFY4 | 3 |
| PLA2G4C | 3 |
| CNTNAP2 | 3 |
| SIGLEC10 | 3 |
| TMEM132A | 3 |
| TMCC3 | 3 |
| RP11-51J9.5 | 3 |
| C1orf21 | 3 |
| EMP1 | 3 |
| PMAIP1 | 3 |
| PROK2 | 3 |
| TNFRSF8 | 3 |
| TRGC1 | 3 |
| CTD-2616J11.2 | 3 |
| C6orf25 | 3 |
| HLA-DRB1 | 3 |
| FGD4 | 3 |
| STEAP3 | 3 |
| RP11-145M9.4 | 3 |
| RP11-707P17.1 | 3 |
| CD1C | 3 |
| ITGAM | 3 |
| FOSB | 3 |
| GNLY | 3 |
| RP11-693J15.5 | 3 |
| COL9A2 | 3 |
| TFCP2L1 | 3 |
| SNORA28 | 3 |
| KLRC4-KLRK1 | 3 |
| NCAM1 | 3 |
| AIF1 | 3 |
| CDKN1A | 3 |
| TRGV9 | 3 |
| AHNAK2 | 3 |
| CDH1 | 3 |
| SPRY2 | 3 |
| ASCL2 | 3 |
| DAPK1 | 3 |
| PVRL2 | 3 |
| FLT3 | 3 |
| RP1-93H18.6 | 3 |
| RP13-39P12.3 | 3 |
| SCD5 | 3 |
| RP11-1055B8.7 | 3 |
| ABCB4 | 3 |
| RP11-296L22.8 | 3 |
| TCL1A | 3 |
| AC007620.3 | 3 |
| FCER1A | 3 |
| FAM26F | 3 |
| ADAM28 | 3 |
| TCL6 | 3 |
| P2RX1 | 3 |
| RXRA | 3 |
| COL8A2 | 3 |
| RNASE6 | 3 |
| HIST2H2BD | 3 |
| FARP1 | 3 |
| RNU1-60P | 3 |
| MGST2 | 3 |
| LRRK1 | 3 |
| ITGA9 | 3 |
| SCGB3A1 | 4 |
| SEC61B | 4 |
| RPS24P8 | 4 |
| COX7A2P2 | 4 |
| OSTCP4 | 4 |
| UQCRH | 4 |
| SUB1P1 | 4 |
| IGHV2-26 | 4 |
| RP3-426I6.2 | 4 |
| RP11-169K16.7 | 4 |
| RPL39L | 4 |
| SNRPE | 4 |
| RP11-601I15.1 | 4 |
| SHFM1P1 | 4 |
| RP11-638I2.10 | 4 |
| PIN4P1 | 4 |
| NPM1P6 | 4 |
| RP5-887A10.1 | 4 |
| ERH | 4 |
| IGHGP | 4 |
| AC007383.6 | 4 |
| COX6B1 | 4 |
| CYSTM1 | 4 |
| LAIR2 | 4 |
| SUB1P3 | 4 |
| RP11-270C12.3 | 4 |
| OST4 | 4 |
| IGLVI-70 | 4 |
| SUSD2 | 4 |
| TBCAP1 | 4 |
| UQCR10 | 4 |
| ALG14 | 4 |
| IGLV2-8 | 4 |
| NME1 | 4 |
| IGLV7-43 | 4 |
| COX7CP1 | 4 |
| RNU11 | 4 |
| RP11-91G21.1 | 4 |
| COX17P1 | 4 |
| TMEM14B | 4 |
| ZBTB8OSP2 | 4 |
| RNU4ATAC | 4 |
| AP000361.2 | 4 |
| RP11-570P14.1 | 4 |
| RPL22L1 | 4 |
| AC013733.5 | 4 |
| MRPS36 | 4 |
| RP11-249L21.4 | 4 |
| IGLV1-51 | 4 |
| LOC124685 | 4 |
| IGKV4-1 | 4 |
| RP11-15E18.5 | 4 |
| IGKV1-6 | 4 |
| ZFAS1 | 4 |
| UBL5P2 | 4 |
| NDUFB3 | 4 |
| MRPS36P1 | 4 |
| RP11-454L1.2 | 4 |
| HMGB2P1 | 4 |
| DGCR11 | 4 |
| MIR29B1 | 4 |
| SNRPEP4 | 4 |
| TMA7 | 4 |
| RLIMP1 | 4 |
| RPS27P27 | 4 |
| RP11-321A17.3 | 4 |
| SCN11A | 4 |
| ATP6V0E1P2 | 4 |
| DLEU2 | 4 |
| RP11-498C9.15 | 4 |
| NDUFB8P2 | 4 |
| EAF2 | 4 |
| RP11-420K14.6 | 4 |
| ISG15 | 4 |
| SELK | 4 |
| IGHV6-1 | 4 |
| OTUB2 | 4 |
| ATP5EP2 | 4 |
| RP11-432I5.1 | 4 |
| RP11-958N24.2 | 4 |
| IGKV3-11 | 4 |
| RP11-609L23.2 | 4 |
| RP11-663P9.2 | 4 |
| C9orf131 | 4 |
| ARPC3P1 | 4 |
| TIMM10 | 4 |
| RPL36AL | 4 |
| STAC3 | 4 |
| RP11-626A1.1 | 4 |
| AC017035.2 | 4 |
| IGLV1-47 | 4 |
| MIR342 | 4 |
| RP11-572P18.1 | 4 |
| COX14 | 4 |
| HAUS1P2 | 4 |
| RP11-386I14.4 | 4 |
| RP11-796G6.1 | 4 |
| C7orf13 | 4 |
| RP1-159A19.3 | 4 |
| HIST1H3D | 4 |
| DBI | 4 |
| CRIP1P4 | 4 |
| BOLA3 | 4 |
| TPRKBP1 | 4 |
| RP1-187B23.1 | 4 |
| CTD-2528L19.3 | 4 |
| IGHV3-66 | 4 |
| RP11-685M7.5 | 4 |
| GSTO3P | 4 |
| RP11-535C21.3 | 4 |
| RP11-28F1.2 | 4 |
| ICAM4 | 4 |
| SIK3-IT1 | 4 |
| MRPL13 | 4 |
| RP13-444K19.1 | 4 |
| RP11-558F24.4 | 4 |
| C12orf45 | 4 |
| NDUFA12 | 4 |
| TIMM8B | 4 |
| AC010336.1 | 4 |
| CLC | 4 |
| TXN | 4 |
| CTD-2561B21.11 | 4 |
| BTBD18 | 4 |
| MT2A | 4 |
| TRAJ38 | 4 |
| NDUFB1P1 | 4 |
| RP11-480I12.2 | 4 |
| APOO | 4 |
| TMSB4XP4 | 4 |
| SLC52A1 | 4 |
| MIR181A1HG | 4 |
| RNU5F-1 | 4 |
| RP1-197O17.2 | 4 |
| VAC14-AS1 | 4 |
| RP11-755B10.4 | 4 |
| RNVU1-6 | 4 |
| BTF3P5 | 4 |
| LY96 | 4 |
| MRPS21P3 | 4 |
| WDR38 | 4 |
| ANAPC10P1 | 4 |
| AL662800.1 | 4 |
| TRAJ35 | 4 |
| AC017104.2 | 4 |
| HMGN5 | 4 |
| SCARNA21 | 5 |
| SNORA12 | 5 |
| CDH2 | 5 |
| RP11-58K22.4 | 5 |
| RP11-474B12.1 | 5 |
| GPR22 | 5 |
| CAP2P1 | 5 |
| RP11-570L15.2 | 5 |
| RP1-3J17.3 | 5 |
| KCNQ1OT1 | 5 |
| RP11-558F24.2 | 5 |
| CHGB | 5 |
| RP11-532F12.6 | 5 |
| MALAT1 | 5 |
| NXPH3 | 5 |
| SCARNA18 | 5 |
| CHD5 | 5 |
| C9orf38 | 5 |
| RP3-340N1.5 | 5 |
| RP11-54O7.11 | 5 |
| ODF3 | 5 |
| RP11-503N18.5 | 5 |
| RN7SK | 5 |
| C1orf200 | 5 |
| ANGPT2 | 5 |
| SUMO2 | 5 |
| MCCD1 | 5 |
| AC093690.1 | 5 |
| MUC6 | 5 |
| RP11-545E17.3 | 5 |
| RP3-323N1.2 | 5 |
| RP13-93L13.2 | 5 |
| SNRPFP1 | 5 |
| RP11-4F22.2 | 5 |
| RP11-613F7.1 | 5 |
| AC024940.1 | 5 |
| RP3-476K8.4 | 5 |
| SNORA54 | 5 |
| MIR3609 | 5 |
| RPS15AP38 | 5 |
| SF3B14 | 5 |
| RP11-47I22.2 | 5 |
| CTD-2024I7.13 | 5 |
| RNU5E-1 | 5 |
| PRAMENP | 5 |
| MAP1B | 5 |
| FST | 5 |
| KARSP3 | 5 |
| TAS2R30 | 5 |
| RPL34P27 | 5 |
| RP11-587D21.1 | 5 |
| SOX10 | 5 |
| RP11-367G18.2 | 5 |
| EEF1A1P3 | 5 |
| NACAD | 5 |
| RP4-604A21.1 | 5 |
| SNORA12 | 5 |
| SCARNA5 | 5 |
| AC096664.1 | 5 |
| AC110781.3 | 5 |
| AKR1B1P2 | 5 |
| HIST1H4D | 5 |
| RP11-254B13.4 | 5 |
| CTC-507E2.1 | 5 |
| PPL | 5 |
| RP11-771F20.1 | 5 |
| AP000769.7 | 5 |
| CASKIN2 | 5 |
| SDS | 5 |
| AC092431.2 | 5 |
| AC006028.11 | 5 |
| WNT4 | 5 |
| RP11-390K5.1 | 5 |
| SCARNA6 | 5 |
| HIST1H4C | 5 |
| RP11-473O4.3 | 5 |
| CHRNA2 | 5 |
| RP5-1049G16.4 | 5 |
| CTD-2561B21.5 | 5 |
| RP11-592N21.1 | 5 |
| AL109767.1 | 5 |
| RP11-44F14.5 | 5 |
| SERPIND1 | 5 |
| RP1-182O16.1 | 5 |
| RP11-172E10.1 | 5 |
| RP11-16C1.2 | 5 |
| SEZ6 | 5 |
| SCARNA21 | 5 |
| MRPL33 | 5 |
| POU5F2 | 5 |
| KLB | 5 |
| PKIB | 5 |
| LPPR3 | 5 |
| RP5-1126H10.2 | 5 |
| DDC8 | 5 |
| RAB26 | 5 |
| DEGS2 | 5 |
| GREB1L | 5 |
| RP11-115D7.3 | 5 |
| ANTXRLP1 | 5 |
| WBP5 | 5 |
| PRSS8 | 5 |
| RP11-731I19.1 | 5 |
| AC016831.7 | 5 |
| SCARNA10 | 5 |
| ZNF90P1 | 5 |
| EDNRB-AS1 | 5 |
| SCARNA17 | 5 |
| RNU4-1 | 5 |
| RP11-171I2.1 | 5 |
| TTYH1 | 5 |
| KCNJ9 | 5 |
| CTC-448F2.4 | 5 |
| MUC5B | 5 |
| TMEM14E | 5 |
| GLI1 | 5 |
| TMSB4XP6 | 5 |
| SNORA73B | 5 |
| AC116366.5 | 5 |
| RP11-247I13.3 | 5 |
| RP11-96D1.9 | 5 |
| RP11-644F5.15 | 5 |
| AL627171.2 | 5 |
| TRAJ23 | 5 |
| HINT1P1 | 5 |
| RP5-1139B12.4 | 5 |
| CTB-47B8.1 | 5 |
| BEX5 | 5 |
| RP11-229M1.2 | 5 |
| RP11-123J14.2 | 5 |
| RP1-89D4.1 | 5 |
| SNORD17 | 5 |
| OR1F2P | 5 |
| RP1-28C20.1 | 5 |
| RP11-138A9.2 | 5 |
| CTC-246B18.10 | 5 |
| RPL35AP21 | 5 |
| FAM65C | 5 |
| FAM189A1 | 5 |
| CHRNB2 | 5 |
| RP11-104O19.2 | 5 |
| U73166.2 | 5 |
| RNU1-59P | 5 |
| CC2D2A | 5 |
| ZNF460 | 5 |
| RPPH1 | 5 |
| CYP2T2P | 5 |
| PLEKHG6 | 5 |
| SCARNA2 | 5 |
| CHRNG | 5 |
| TMEM256 | 5 |
| AC016586.1 | 5 |
| ACPT | 5 |
| SCARNA7 | 5 |
| RMRP | 5 |
| MYL6P3 | 5 |
| HIST1H1E | 5 |
| NPM1P33 | 5 |
| PDGFRL | 5 |
| AC096558.1 | 5 |
| RP1-102E24.1 | 5 |
| RP11-266L9.2 | 5 |
| RNU4-2 | 5 |
| FAM196B | 5 |
| LRRC8E | 5 |
| RP1-40G4P.1 | 5 |
| RP11-347P5.1 | 5 |
| HNRNPA3P15 | 5 |
| RP11-389O22.4 | 5 |
| CTD-3065B20.1 | 5 |
| ANKRD44-IT1 | 5 |
| RP11-127B20.2 | 5 |
| RP11-166N17.3 | 5 |
| RSL24D1P1 | 5 |
| CTB-109A12.1 | 5 |
| RP11-44M6.3 | 5 |
| RPS12P20 | 5 |
| RPL5P17 | 5 |
| RP4-591N18.2 | 5 |
| BTF3P10 | 5 |
| ITGB4 | 5 |
| RP11-138A9.1 | 5 |
| GPX2 | 5 |
| RP11-409K20.6 | 5 |
| AC021016.7 | 5 |
| PEX11G | 5 |
| CDKN2AIPNLP1 | 5 |
| RP5-1085F17.4 | 5 |
| RN7SL2 | 5 |
| MUC12 | 5 |
| TLCD1 | 5 |
| TRAJ29 | 5 |
| SNORD15B | 5 |
| ESRP2 | 5 |
| RP11-358B23.5 | 5 |
| FKBP4P1 | 5 |
| CDHR5 | 5 |
| TENC1 | 5 |
| RP4-575N6.2 | 5 |
| RP11-293G6 | 5 |
| C19orf35 | 5 |
| RP11-44N11.1 | 5 |
| RN7SL1 | 5 |
| SNORA53 | 5 |
| RP11-1023L17.2 | 5 |
| CTD-3035D6.1 | 5 |
| A2M | 5 |
| ADAMTSL4-AS1 | 5 |
| CTA-276O3.4 | 5 |
| RP5-857K21.11 | 5 |
| AC012066.1 | 5 |
| RP11-328J14.2 | 5 |
| SLC10A1 | 5 |
| TRAJ26 | 5 |
| RP11-829H16.2 | 5 |
| RP11-75C10.9 | 5 |
| RP1-102E24.6 | 5 |
| RPL31P63 | 5 |
| RP11-459A10.1 | 5 |
| RP11-571F15.3 | 5 |
| AC127904.2 | 5 |
| RP11-616K22.1 | 5 |
| SCARNA17 | 5 |
| RP1-181J22.1 | 5 |
