# Supplementary material for: CD4+ T cells from children with active juvenile idiopathic arthritis show altered chromatin features associated with transcriptional abnormalities
Source: Sci Rep. 2021 Feb 17;11:4011. doi: 10.1038/s41598-021-82989-5 (PMC7889855; doi:10.1038/s41598-021-82989-5)
Supplement: Supplementary file 7 — Supplementary Table 5. [file 41598_2021_82989_MOESM7_ESM.docx]

**Table S5**

GO term enrichment for the five clusters of differentially expressed genes identified in Figure 1B.

| **GO Term** | **Description** | **P-value** | **Cluster** |
| --- | --- | --- | --- |
| GO:0006958 | Complement activation, classical pathway | 6.80E-08 | 1 |
| GO:0006956 | Complement activation | 1.31E-07 |  |
| GO:0006412 | Translation | 1.58E-07 |  |
| GO:0072376 | Protein activation cascade | 3.16E-07 |  |
| GO:0043043 | Peptide biosynthetic process | 3.22E-07 |  |
| GO:0000395 | mRNA 5'-splice site recognition | 3.71E-07 |  |
| GO:0022900 | Electron transport chain | 1.39E-06 |  |
| GO:0006518 | Peptide metabolic process | 9.08E-06 |  |
| GO:0006959 | Humoral immune response | 9.51E-06 |  |
| GO:0022904 | Respiratory electron transport chain | 1.18E-05 |  |
|  |  |  |  |
| GO:0007186 | G protein-coupled receptor signaling pathway | 8.19E-06 | 2 |
| GO:0048583 | Regulation of response to stimulus | 2.78E-05 |  |
| GO:0030334 | Regulation of cell migration | 6.92E-05 |  |
| GO:0051895 | Negative regulation of focal adhesion assembly | 1.00E-04 |  |
| GO:2000145 | Regulation of cell motility | 1.25E-04 |  |
| GO:1903392 | Negative regulation of adherens junction organization | 1.85E-04 |  |
| GO:0040012 | Regulation of locomotion | 2.51E-04 |  |
| GO:0051270 | Regulation of cellular component movement | 2.62E-04 |  |
| GO:0051893 | Regulation of focal adhesion assembly | 2.70E-04 |  |
| GO:0090109 | Regulation of cell-substrate junction assembly | 2.70E-04 |  |
|  |  |  |  |
| GO:0006614 | SRP-dependent cotranslational protein targeting to membrane | 2.63E-35 | 3 |
| GO:0006613 | Cotranslational protein targeting to membrane | 1.11E-34 |  |
| GO:0045047 | Protein targeting to ER | 1.55E-33 |  |
| GO:0072599 | Establishment of protein localization to endoplasmic reticulum | 3.22E-33 |  |
| GO:0019083 | Viral transcription | 1.63E-32 |  |
| GO:0000184 | Nuclear-transcribed mRNA catabolic process, nonsense-mediated decay | 3.16E-32 |  |
| GO:0070972 | Protein localization to endoplasmic reticulum | 3.92E-32 |  |
| GO:0006413 | Translational initiation | 9.59E-31 |  |
| GO:0006612 | Protein targeting to membrane | 1.57E-29 |  |
| GO:0006412 | Translation | 9.12E-29 |  |
|  |  |  |  |
| GO:0002376 | Immune system process | 3.75E-16 | 4 |
| GO:0006955 | Immune response | 1.19E-11 |  |
| GO:0001775 | Cell activation | 2.17E-10 |  |
| GO:0002250 | Adaptive immune response | 7.99E-10 |  |
| GO:0045321 | Leukocyte activation | 1.54E-09 |  |
| GO:0050896 | Response to stimulus | 2.19E-09 |  |
| GO:0002503 | Peptide antigen assembly with MHC class II protein complex | 1.33E-08 |  |
| GO:0007165 | Signal transduction | 2.83E-08 |  |
| GO:0002252 | Immune effector process | 1.03E-07 |  |
| GO:0002682 | Regulation of immune system process | 1.18E-07 |  |
|  |  |  |  |
| GO:0034723 | DNA replication-dependent nucleosome organization | 2.06E-04 | 5 |
| GO:0006335 | DNA replication-dependent nucleosome assembly | 2.06E-04 |  |
| GO:0000183 | Chromatin silencing at rDNA | 2.86E-04 |  |
| GO:0006342 | Chromatin silencing | 8.59E-04 |  |
| GO:1905049 | Negative regulation of metallopeptidase activity | 8.72E-04 |  |
