# Supplementary material for: CD4+ T cells from children with active juvenile idiopathic arthritis show altered chromatin features associated with transcriptional abnormalities
Source: Sci Rep. 2021 Feb 17;11:4011. doi: 10.1038/s41598-021-82989-5 (PMC7889855; doi:10.1038/s41598-021-82989-5)
Supplement: Supplementary file 8 — Supplementary Table 6. [file 41598_2021_82989_MOESM8_ESM.docx]

**Table S6**

GO term enrichment for the various groups of ATAC peaks from Figure 2A

| **Description** | **FDR** | **Group** |
| --- | --- | --- |
| homophilic cell adhesion via plasma membrane adhesion molecules | 2.73E-04 | ADT + CRM |
| gamma-aminobutyric acid signaling pathway | 9.13E-03 |  |
| negative regulation of extrinsic apoptotic signaling pathway in absence of ligand | 9.75E-03 |  |
| inorganic anion transmembrane transport | 2.27E-02 |  |
| positive regulation of JAK-STAT cascade | 2.72E-02 |  |
| thorax and anterior abdomen determination | 3.05E-02 |  |
| negative regulation of relaxation of muscle | 3.05E-02 |  |
| regulation of alkaline phosphatase activity | 4.73E-02 |  |
| interleukin-7-mediated signaling pathway | 4.80E-04 |  |
| cellular response to interleukin-7 | 6.42E-04 |  |
| gamma-aminobutyric acid signaling pathway | 3.94E-02 | ADT + HC |
| body fluid secretion | 1.85E-02 |  |
| CENP-A containing nucleosome assembly | 5.10E-04 |  |
| DNA replication-independent nucleosome assembly | 2.69E-03 |  |
| DNA replication-independent nucleosome organization | 3.40E-03 |  |
| chromatin remodeling at centromere | 1.67E-03 |  |
| centromere complex assembly | 4.55E-03 |  |
| ammonium transmembrane transport | 9.03E-05 |  |
| histone exchange | 6.35E-03 |  |
| DNA replication-dependent nucleosome assembly | 2.54E-12 |  |
| gamma-aminobutyric acid signaling pathway | 3.82E-03 | HC + CRM |
| inorganic anion transmembrane transport | 1.08E-02 |  |
| positive regulation of JAK-STAT cascade | 2.48E-02 |  |
| anion transmembrane transport | 1.74E-02 |  |
| regulation of alkaline phosphatase activity | 2.84E-02 |  |
| negative regulation of extrinsic apoptotic signaling pathway in absence of ligand | 1.59E-02 |  |
| antigen processing and presentation of endogenous peptide antigen via MHC class I | 3.33E-04 |  |
| interleukin-7-mediated signaling pathway | 1.16E-04 |  |
| cellular response to interleukin-7 | 1.57E-04 |  |
| antigen processing and presentation of exogenous peptide antigen via MHC class I, TAP-independent | 1.99E-02 |  |
| gamma-aminobutyric acid signaling pathway | 3.31E-03 | ADT + HC + CRM |
| inorganic anion transmembrane transport | 2.43E-02 |  |
| positive regulation of alkaline phosphatase activity | 4.89E-02 |  |
| anion transmembrane transport | 2.81E-02 |  |
| regulation of alkaline phosphatase activity | 2.75E-02 |  |
| negative regulation of extrinsic apoptotic signaling pathway in absence of ligand | 1.47E-02 |  |
| regulation of megakaryocyte differentiation | 4.22E-06 |  |
| antigen processing and presentation of endogenous peptide antigen via MHC class I | 2.99E-04 |  |
| interleukin-7-mediated signaling pathway | 9.86E-05 |  |
| cellular response to interleukin-7 | 1.29E-04 |  |
| ammonium transmembrane transport | 3.63E-03 | ADT |
| positive regulation of response to external stimulus | 4.76E-02 |  |
| ammonium transport | 1.42E-02 |  |
| antigen processing and presentation of exogenous peptide antigen via MHC class I, TAP-independent | 4.47E-02 | CRM |
| negative regulation of gene expression, epigenetic | 1.77E-05 |  |
| negative regulation of relaxation of muscle | 4.47E-02 |  |
| regulation of gene expression, epigenetic | 3.17E-03 |  |
| antigen processing and presentation | 2.15E-02 |  |
| inorganic anion transmembrane transport | 3.87E-02 |  |
| antigen processing and presentation of exogenous antigen | 1.49E-02 |  |
| negative regulation of extrinsic apoptotic signaling pathway in absence of ligand | 4.32E-02 |  |
| interferon-gamma-mediated signaling pathway | 7.73E-03 |  |
| thorax and anterior abdomen determination | 4.47E-02 |  |
| trace-amine receptor activity | 3.07E-02 | HC |
