# Supplementary material for: CD4+ T cells from children with active juvenile idiopathic arthritis show altered chromatin features associated with transcriptional abnormalities
Source: Sci Rep. 2021 Feb 17;11:4011. doi: 10.1038/s41598-021-82989-5 (PMC7889855; doi:10.1038/s41598-021-82989-5)
Supplement: Supplementary file 9 — Supplementary Table 7. [file 41598_2021_82989_MOESM9_ESM.docx]

**Table S7**

Differentially bound CTCF sites from Figure 2B. Entries in *italics* were further characterized in Supplemental Figures 1 and 2.

| **Chromosome** | **Start** | **End** | **Log(CPM)** | **Log(fold-change)** | **P-Value** |
| --- | --- | --- | --- | --- | --- |
| chr19 | 48412416 | 48413188 | 7.500625 | -1.5231 | 1.11E-05 |
| chr8 | 125049733 | 125050571 | 7.352681 | -1.09038 | 9.86E-06 |
| *chr17* | *29297031* | *29297692* | *5.48387* | *-4.99065* | *1.54E-06* |
| *chr10* | *98549241* | *98549844* | *5.48196* | *5.519167* | *2.95E-08* |
| chr1 | 240618532 | 240618949 | 5.004954 | 1.484664 | 1.23E-05 |
| chr12 | 25113117 | 25113589 | 4.720119 | -1.19967 | 1.52E-05 |
| chr17 | 57401515 | 57401857 | 4.570775 | 6.228704 | 4.55E-29 |
| chr11 | 35163919 | 35164360 | 4.549561 | 4.234084 | 1.10E-05 |
| chr11 | 35409153 | 35409523 | 4.006438 | 4.94806 | 2.10E-06 |
| chr20 | 51054820 | 51055278 | 3.999501 | -3.74815 | 9.57E-07 |
| chr14 | 77466765 | 77467154 | 3.159566 | -2.23086 | 2.12E-05 |
| chr12 | 133006095 | 133006564 | 3.138412 | 4.472428 | 6.56E-06 |
| chr1 | 249238374 | 249239135 | 3.071542 | 3.198109 | 3.93E-06 |
| chr19 | 33210455 | 33210740 | 3.01333 | 3.255775 | 1.08E-05 |
| chr21 | 30243812 | 30244063 | 2.959687 | 4.293635 | 3.63E-06 |
| chr2 | 98641763 | 98642304 | 2.894842 | 3.117872 | 1.52E-07 |
| chr17 | 6659065 | 6659499 | 2.843898 | -2.39969 | 1.63E-06 |
| chr1 | 144859829 | 144860046 | 2.826194 | 2.188316 | 5.35E-06 |
| chr12 | 84891660 | 84891938 | 2.805286 | 2.372102 | 1.82E-05 |
| chr17 | 20569960 | 20570461 | 2.795766 | -2.22617 | 1.42E-05 |
| chr4 | 130670650 | 130671100 | 2.739895 | -2.59916 | 2.34E-06 |
| chr5 | 87486338 | 87486598 | 2.733747 | 3.598423 | 3.01E-06 |
| chr1 | 64955890 | 64956168 | 2.716688 | 2.887749 | 3.67E-07 |
| chr12 | 132699441 | 132699716 | 2.526912 | 2.751399 | 1.40E-07 |
| chrX | 112157095 | 112157412 | 2.302638 | 2.471822 | 7.51E-06 |
| chr20 | 46163806 | 46164085 | 2.256689 | 2.373261 | 5.96E-06 |
| chr6 | 156204591 | 156204783 | 2.181708 | 2.984329 | 5.66E-06 |
| chr3 | 27670932 | 27671129 | 2.092766 | 2.521057 | 1.68E-05 |
| chr5 | 102398230 | 102398560 | 1.92659 | 3.183585 | 2.12E-05 |
| chr12 | 127510128 | 127510387 | 1.786453 | 3.027158 | 8.34E-06 |
| chr22 | 37683717 | 37683936 | 1.663587 | 3.190686 | 1.38E-05 |
