# Supplementary material for: CD4+ T cells from children with active juvenile idiopathic arthritis show altered chromatin features associated with transcriptional abnormalities
Source: Sci Rep. 2021 Feb 17;11:4011. doi: 10.1038/s41598-021-82989-5 (PMC7889855; doi:10.1038/s41598-021-82989-5)
Supplement: Supplementary file 10 — Supplementary Legends. [file 41598_2021_82989_MOESM10_ESM.pdf]

Supplemental Figure Legends

CD4+ T cells From Children With Active Juvenile Idiopathic Arthritis Show Altered Chromatin Features Associated With Transcriptional Abnormalities

Evan Tarbell,* [1,3,5] Kaiyu Jiang* [2], Teresa R. Hennon[2], Lucy Holmes[2], Sonja Williams[2], Yao Fu,[4] Patrick M. Gaffney,[4] Tao Liu[1,3,6], James N. Jarvis[2,3] ##

[1] Department of Biochemistry, University at Buffalo Jacobs School of Medicine and Biomedical Sciences, Buffalo, NY, USA
[2] Department of Pediatrics, University at Buffalo Jacobs School of Medicine and Biomedical Sciences, Buffalo, NY, USA
[3] Genetics, Genomics, & Bioinformatics Program, University at Buffalo Jacobs School of Medicine and Biomedical Sciences, Buffalo, NY, USA
[4] Genes and Human Disease Research Program, Oklahoma Medical Research Foundation, Oklahoma City, OK, USA
[5] Enhanced Pharmacodynamics LLC, Buffalo, NY, USA
[6] Department of Biostatistics and Bioinformatics, Roswell Park Comprehensive Cancer Center, Buffalo, NY, USA

Fig. S1. Active JIA shows Gain of CTCF Binding at PIK3AP1-LCOR Locus

A) Screenshot showing a gain of CTCF binding event on chromosome 10, in the PIK3AP1-LCOR locus. B) Individual CTCF binding profiles at the locus, labeled by group and Sanger sequencing confirmed genotype (See Methods). C) Expression of differentially expressed genes, PIK3AP1 and LCOR, connected to Gain of Binding site across conditions.

Fig. S2. Active JIA shows Loss of CTCF Binding at RNF135 Locus

A) Screenshot showing a loss of CTCF binding event on chromosome 17 near the RNF135 promoter. B) Individual CTCF binding profiles at the locus, labeled by group and Sanger sequencing confirmed genotype (See Methods). C) Expression of differentially expressed gene, RAB11FIP4, connected to Loss of Binding site across conditions.
